# Gradient-graphene-enabled directional photothermal regulation for self-aligned laser transfer printing

**DOI:** 10.1038/s41377-025-02170-9

**Published:** 2026-01-12

**Authors:** Mengxin Gai, Jing Bian, Furong Chen, Lei Liu, Yu Luo, Yuxing Ma, Xincheng Huang, Hong Xiao, YongAn Huang

**Affiliations:** 1 State Key Laboratory of Intelligent Manufacturing Equipment and Technology, Huazhong University of Science and Technology, Wuhan, China; 2 Flexible Electronics Research Center, Huazhong University of Science and Technology, Wuhan, China; 3 School of Microelectronics (School of Integrated Circuits), Nanjing University of Science and Technology, Nanjing, China

**Keywords:** Laser material processing, Inorganic LEDs

## Abstract

Laser-assisted transfer printing has gained attention for integrating microdevices on unusual substrates. However, conventional technologies exhibit limited fault tolerance during laser-matter interactions, reducing transfer accuracy due to unavoidable irradiation deviations. We report a self-aligned laser transfer (SALT) that enables high-precision, programmable assembly of microchips without precise laser-to-die alignment. A thermal conductivity gradient carbon (TCGC), with an upper graphene layer and lower amorphous carbon layer, is embedded in the stamp via excimer laser self-limited carbonization of polyimide. The TCGC converts asymmetric light input into uniform heat output under non-uniform/misaligned infrared laser irradiation, whereas the upper graphene layer absorbs heat from the lower amorphous carbon and rapidly conducts heat laterally, ensuring uniform heat distribution of the underlying adhesive layer. This guarantees synchronous chip release at all adhesive sites, mitigating transfer deviations. Additionally, periodically arranged, grayscale-controlled TCGC can be fabricated by modulating excimer laser parameters during carbonization, thereby enabling selective microchip release without pre-planned scanning paths. SALT achieves excellent size compatibility ( < 100 micrometers) and high tolerance for irradiation deviations (transfer accuracy <5 micrometers). Demonstrations of RGB micro-LED display highlight its self-aligned and batch-selective capabilities.

## Abstract (web summary)

Self-aligned laser transfer technique achieves high-precision microchip assembly without precise alignment, enabled by gradient-graphene stamp that converts asymmetric light input into uniform heat output, effectively mitigating laser irradiation deviations.

## Graphical abstract

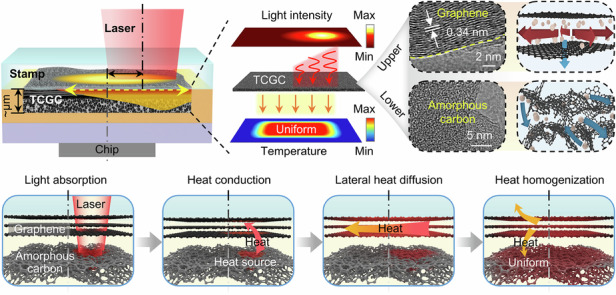

## Introduction

The integration of massive arrays of miniaturized electronics onto unusual substrates has enabled the development of the most advanced electronic systems, including micro-light-emitting diode (MicroLED) displays^1,2^, flexible optoelectronics^3,4^, biosensor arrays for health monitoring and diagnosis^5,6^, and tunable electromagnetic meta-surfaces^7,8^. The fabrication of these innovative systems relies heavily on the heterogeneous integration of micro-electronic components (such as MicroLED chips^9,10^, micro-sensors^6^, diodes chips^11–14^) onto universal receivers with high assembly efficiency (up to ~100 million·h^−^^1^^15^) and high transfer accuracy (less than ~5 μm^16^). As a versatile heterogeneous integration technique, micro transfer printing (μTP) facilitates the assembly of diverse microchips into desired layouts using an adhesive stamp. The key for μTP lies in the high-efficient and reliable modulation of the stamp-chip interfacial adhesion, transitioning from a strong state for pick-up to a weak state for accurate printing. Significant efforts have been made to develop stamps with tunable adhesion based on various control mechanisms, including the stamp peeling rate^17,18^, micro-vacuum force^19^, electrostatic force^20,21^, and fluidic self-assembly^22,23^. However, these contact transfer methods still face critical challenges, such as the limited switchable adhesion capability and poor selectivity with chip-level addressability.

Owing to its rapid and selective infinite adhesion switching capability with massively parallel processing, laser-assisted non-contact μTP shows promising prospects for industrial production^24–26^. During the laser transfer, a laser beam passes through a transparent substrate and irradiates the laser-sensitive layer, causing chemical or physical reactions at the interface that enable microchips to overcome stamp adhesion. Based on the mechanism of laser-matter interaction, this process can be categorized into two types. (1) Ultraviolet (UV) laser ablation: the laser sensitive layer absorbs UV laser to generate gas^27,28^/blister thrust^29^, detaching the device. This ablation mode exhibits excellent response time ( < 1 μs^30^); however, the chemical reaction is irreversible (preventing stamp reuse), and complex dynamics of blister expansion makes transfer precision uncontrollable^29,31^. (2) Photothermal-induced transfer: the interfacial laser-sensitive material absorbs and converts the laser (i.e., visible^32^/infrared laser^33^) into heat, leading to the release of the chip from the stamp via thermally induced adhesive reduction mechanisms (e.g., thermal mismatch^34^, shape change^9,10,35^ or pneumatic actuation^36,37^). Despite the notable advances in laser-induced reversible adhesives (e.g., shape memory polymer^10,38,39^, liquid metal^40^, and hydrogel^41^), none of them can achieve high transfer accuracy in rapid processing manners (using array lasers^16^ or galvanometer-based scanning^9^). The fundamental problem is that current laser transfer techniques lack fault tolerance during the laser-matter interactions. This leads to non-uniform heat distribution at the stamp-chip interface due to irradiation deviations that are challenging to eliminate in practical applications^16,42,43^, thereby inducing asymmetric chip peeling behavior and unpredictable flight trajectories. Recent efforts have introduced self-aligned strategies using magnetic^44^ or fluidic aids^22,23^ to reduce chip offset errors, but these approaches increase costs and manufacturing complexity. Instead of intentionally moving the chip on the receiving substrate, a more feasible misalignment tolerance enhancement strategy is to directionally control the energy transfer path during laser-induced chip delamination (i.e., photothermal conversion, heat transfer, and thermally induced delamination), so that the chip transfer path can be independent of irradiation deviations. This self-alignment strategy strongly relies on the directional photothermal regulation during laser-matter interactions, which is crucial for achieving high reliability but has not yet been realized with existing laser transfer methods.

Here, we report a self-aligned laser transfer (SALT) based on directional photothermal regulation strategies that enables high-precision, programmable transfer of microchips without the need of precise laser-to-die alignment. The key innovation lies in the introduction of a special photothermal conversion material, i.e., thermal conductivity gradient carbon (TCGC). The TCGC can be prepared using a UV excimer laser to induce confined, self-limited carbonization of polyimide (PI), which naturally creates a gradient distribution of graphitization degree, with graphene (Gr) layer at the top and amorphous carbon (AC) layer at the bottom. The unique gradient structure facilitates anisotropic and non-uniform spatial thermal conductivity distribution, thereby controlling the intensity of heat conduction in different directions. Systematically experimental and numerical studies have revealed the self-aligned mechanism, wherein the TCGC enables simultaneous laser absorption and directional heat conduction to non-ideal irradiated regions under non-uniform/misaligned infrared laser irradiation. This efficient thermal homogenization ensures the synchronous release of a chip across all adhesive positions on the stamp, thereby mitigating the impact of irradiation deviations on the chip transfer path. Additionally, the periodically arranged, grayscale-controlled TCGC can selectively release microchips without pre-planned scanning paths, offering distinct advantages in chip throughput for batch selective transfer and high-tolerance to irradiation deviation in the densely arranged chip arrays. The SALT has enabled the heterogeneous integration and selective transfer of diverse micro-objects with varying shapes, sizes, and patterns onto various challenging surfaces, demonstrating reversible adhesion switchability of ~650, rapid response time ( ~ 30 ms), excellent size compatibility (from 100 μm to 1 mm), and high tolerance for irradiation deviations (transfer accuracy < 5 μm under a 30% beam offset). Moreover, the successful integration of microchips onto three-dimensional substrates demonstrates the potential of SALT for advancing curved electronics. Demonstrations involving multiple transfer printings of RGB MicroLED chips from different donor wafers highlight SALT’s self-aligned and batch-selective capabilities, which are crucial for efficient full-color MicroLED display assembly.

## Results

### Concept of TCGC-assisted self-aligned laser transfer printing

**Fig. 1:**
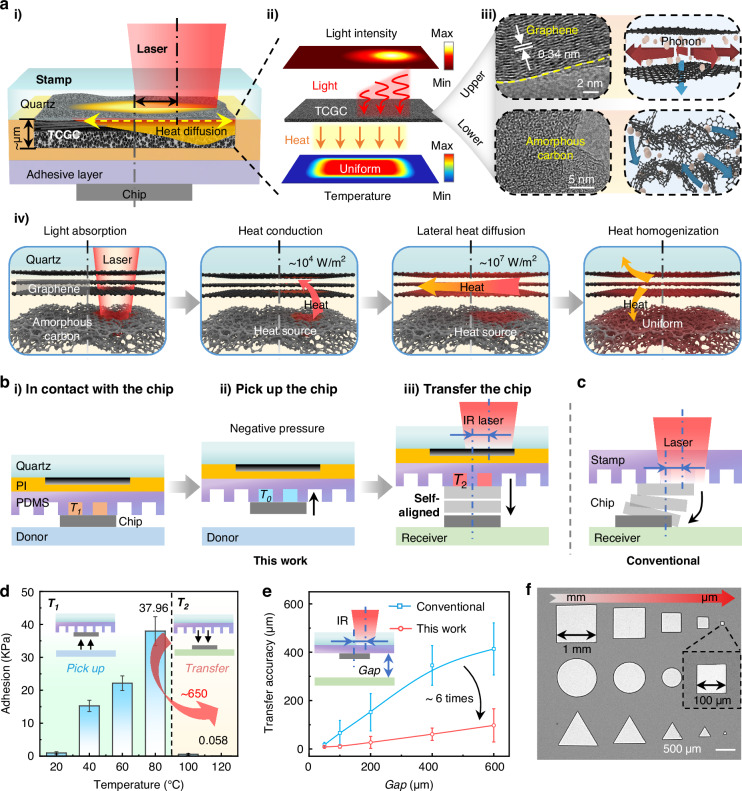
Concept and experimental results of TCGC-assisted self-aligned laser transfer printing. **a** Schematic illustration of the stamp with TCGC: (i) Schematic of the self-aligned mechanism of TCGC. (ii) Conversion of an asymmetric light intensity input to an even heat output by TCGC. (iii) Composition of TCGC: the upper layer is graphene with ordered atomic arrangement and high phonon transport efficiency; the lower layer is amorphous carbon with disordered atomic structure and low phonon transport efficiency. (iv) Schematic of thermal homogenization by light absorption and directional heat conduction through TCGC. **b** Schematic illustration of the pick-up and printing process of SALT: (i) Heated stamp is in contact with the chip. (ii) The stamp picks up the chip from the donor substrate using negative pressure. (iii) IR laser irradiation enables chip transfer from the stamp. **c** Schematic illustration of chip transfer errors caused by irradiation deviations in conventional laser transfer techniques. **d** Adhesion strength test results at different temperature states. **e** Comparison of chip transfer accuracy under IR laser offset irradiation, conventional approaches (without TCGC) and this work. **f** SEM images of different patterned chips (triangles, circles and squares) transferred by SALT

Previous laser-assisted transfer techniques required each chip to be carefully aligned before laser irradiation (i.e., point-by-point transfer^37–39,41,45^). However, even in such a process, perfect alignment through optical means (e.g., visual positioning, with an error of ~1 µm^46^) cannot be realized due to factors such as visual alignment errors^42^, asymmetric chip geometry^47^, and aberrations of beam profile^16^. Moreover, this operation mode is not applicable when extremely high transfer efficiency is demanded, which typically requires high-speed scanning (by a galvanometer mirror^9^) or arrayed laser spots (through beam splitting^48^) to massively improve the transfer efficiency. As illustrated in Fig. 1a-i and a-ii, during high-speed scanning, irradiation deviations (non-uniform or misaligned irradiation on the target chip^41,49^) are difficult to avoid (see Figure S1a), which may be caused by various factors, such as platform vibration^43^, laser oblique incidence^50^, and thermal drift^51^. The irradiation deviations give rise to an uneven temperature distribution and asymmetric crack propagation at the stamp-chip interface, resulting in an unpredictable chip transfer path that significantly reduces the transfer accuracy, as schematically illustrated in Fig. 1c and Figure S2.

The SALT comprises two principal steps, that is, self-homogenized photothermal-conversion and thermal controlled chip release. In the photothermal-conversion process, a specially fabricated carbon layer with µm-thickness is employed to convert the infrared (IR) laser into heat (Fig. 1a-ii). Different from conventional photothermal materials (e.g., carbon black^33^, metal^36,39^, black dye^49^, and chip itself^37,38^), our carbon-based photothermal layer (i.e., TCGC) has a gradient change in thermal conductivity along the thickness direction. As shown in Fig. 1a-iii, the upper layer consists of graphene (Gr) with excellent in-plane thermal conductivity^52^, while the bottom layer is amorphous carbon (AC). Essentially, the top Gr layer exhibits a lattice spacing of ~0.34 nm, indicating an ordered structure comprised of sp^2^-hybridized carbon atoms arranged in a crystal lattice, which is highly efficient for phonon transport [thermal conductivity of ~10^3 ^W·m^−^^1^·K^−^^1^)^53^]. In contrast, the poor structural regularity of amorphous carbon results in a much lower thermal conductivity [~10^−^^2 ^W·m^−^^1^·K^−^^1^^53^]. As illustrated in Fig. 1a-iv, when the TCGC undergoes under non-uniform/misaligned laser irradiation (Fig. 1a-ii) through a transparent substrate (quartz glass), both the Gr and AC layer can act as powerful light-to-heat converters (due to the sp^2^-hybridized carbon atoms). Notably, the glass substrate (acting as a heat sink) causes the highest temperature to be in the AC layer (heat source), rather than the Gr layer (Figure S3). This forms an efficient heat-dissipation design, where the heat generated by the AC layer will be largely absorbed ( ~ 80%) by the top Gr layer and rapidly diffused laterally ( ~ 10^7 ^W·m^−^^2^), enabling thermal homogenization by directional heat conduction to the non-irradiated regions. As shown in Fig. 1a-ii, the unique gradient structure of TCGC facilitates the conversion of an asymmetric light intensity input into an even heat output, thereby ensuring a uniform temperature distribution beneath the TCGC. Consequently, the thermal controlled adhesive layer obtains almost synchronous release of the chip at all adhesion sites due to the uniform temperature (otherwise, a certain adhesive region would release the chip earlier, leading to transfer errors, as shown in Figure S2d), exhibiting the self-alignment capability without sacrificing the response time of laser-induced chip transfer.

The fabrication of a high-quality TCGC with robust self-aligned capability is essential. As shown in Figure S4a, a pre-designed pattern of a thin polyimide (PI) film on a transparent substrate (i.e., quartz) can be one-step transformed into TCGC by UV-laser interfacial carbonization. Unlike conventional surface laser-induced-carbonization, the interface irradiation prevents the escape of carbonization products, which will spontaneously transform into TCGC through hundreds of irradiations^54^, whose thickness, matter distribution, and photothermal-conversion ability can be controlled by adjusting the irradiation number. The thermal controlled adhesive layer made of polydimethylsiloxane (PDMS) with microcavities is then directly assembled onto the TCGC layer to form the stamp (Figure S4b). Figure 1b illustrates the TCGC-assisted self-aligned laser transfer printing. During the pick-up process, the stamp is in contact with the chip and aligned with the TCGC (Fig. 1b-i). The stamp is initially heated to *T*_1_ (above the ambient temperature *T*_0_) before contacting the chip, and then cooled back to *T*_0_ with the chip, generating negative pressure in the microcavities (Fig. 1b-ii). The chip can be picked up owing to the synergy of PDMS adhesion and physical suction of the microcavities. Figure 1d presents the thermal controlled adhesion of the stamp (the testing method is detailed in Figure S5), the adhesion increases along with *T*_1_ and reaches 37.96 kPa when *T*_1_ = 80 °C, which is comparable to the adhesion of a common flat PDMS stamp^55^, ensuring sufficient adhesion for the pick-up process. For chip release, an IR laser (wavelength of 808 nm) with power intensity above a threshold irradiates the TCGC above the target chip (Fig. 1b-iii). The heated stamp (*T*_2_) will drive the chip transfer due to the thrust force generated by the elevated gas pressure within the microcavities. The test results indicate a weak adhesion of 0.058 kPa when *T*_2_ reaches 120 °C, guaranteeing a high adhesion switching ratio ( > 650) for reliable chip release. In contrast to the conventional laser-assisted transfer techniques (irradiation deviations lead to transfer errors, as shown in Fig. 1c), the TCGC’s thermal self-homogenized capability in SALT ensures high-precision chip transfer without precise laser-to-die alignment. As shown in Fig. 1e, when the incident laser spot (400 µm in diameter) is misaligned with the target chip (600 µm × 600 µm) by an offset of 200 µm, the TCGC-assisted transfer shows a significantly higher transfer accuracy, improving by ~6 times at different gaps compared to conventional approaches (without TCGC).

The preparation of stamp is simple, scalable, and highly customizable. Optical images of a 2-inch stamp with a 27 × 27 array of TCGC are shown in Figure S6, which can be quickly fabricated through an optical mask, exhibiting excellent processing resolution. This is further evidenced by a micro-patterned TCGC with a minimum achievable size of 30 µm and a pitch of 10 µm (Figure S7), confirming its suitability for applications requiring high spatial density, such as MicroLED displays^56^. The TCGC pattern and thermal controlled adhesive microstructures can be adjusted to adapt to different sizes of transfer objects. As shown in Fig. 1f, chips with different shapes and dimensions (ranging from 100 µm to 1 mm) have been integrated onto a same substrate, demonstrating excellent size compatibility of SALT. Moreover, the combination of ultrathin TCGC and thermal controlled adhesive layer facilitates efficient utilization of laser energy through efficient light absorption, rapid thermal homogenization, and heat-induced gas expansion. Only a short irradiation time ( < 30 ms) with low laser power ( < 0.5 W) is required (Figure S8), and the typical energy density demanded for transferring a unit area of a chip is significantly lower than that reported in previous IR laser-driven transfer techniques, as quantitatively compared in Table S1. The efficient utilization of laser energy and the self-alignment of the laser-to-die render SALT highly promising for mass transfer applications that demand extremely high transfer efficiency and accuracy.

### Fabrication and performance optimization of TCGC-embedded adhesive stamps

**Fig. 2:**
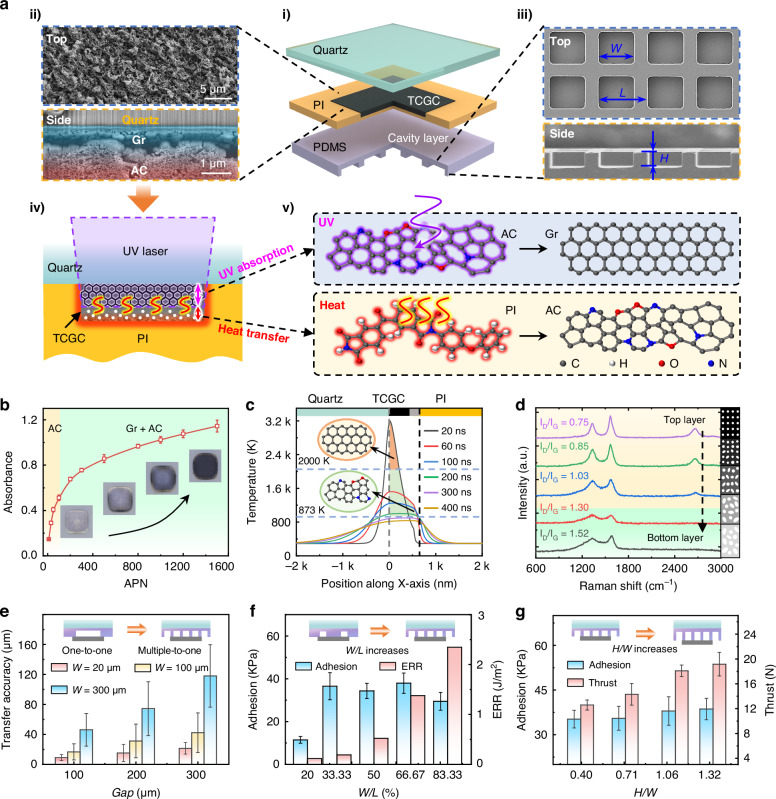
Fabrication and performance optimization of TCGC-embedded adhesive stamps. **a** Schematic illustration of a TCGC-embedded adhesive stamp: (i) Exploded view of the stamp. (ii) and (iii): SEM images of the TCGC and microcavity array. (iv) and (v): Schematic diagrams of TCGC preparation. **b** IR absorbance of carbonized products prepared using different APN of UV laser. **c** Temperature field distribution in PI layer during UV laser ablation. **d** Layer-by-layer Raman spectral analysis of TCGC. **e** Measured transfer accuracy of chips corresponding to different cavity widths at different gaps when the cavity temperature is 120 °C. **f** Effect of microcavity ratios (*W*/*L*) on the measured adhesion (cooling from 80 °C to 20 °C) and simulated interfacial crack tip ERR under a 0.5 W IR laser irradiation for 60 ms. **g** Influence of microcavity height-to-width ratios (*H*/*W*) on the measured adhesion (cooling from 80 °C to 20 °C) and thrust force (heating from 20 °C to 120 °C)

Figure 2a-i presents an exploded sectional illustration of the stamp, with fabrication details provided in Figure S9. As highlighted in Fig. 2a-ii, TCGC with a unique gradient structure (Gr-AC) is sandwiched between a quartz glass and a thin PI film. Figure 2a-iv presents a schematic diagram of the laser carbonization process for transforming interfacial PI into TCGC. Due to the presence of the internal aromatic ring, PI is a common source for laser-induced carbonization^57^, which can strongly absorb UV laser and enable breakage and reunition of carbon atoms at high temperatures. Distinct from the conventional surface carbonization, when the laser irradiates at the interface, the carbonization products are confined between the unreacted PI layer and the transparent substrate, enabling further reactions by subsequent laser pulses. Previous studies have verified that reaction products [including gas and carbonization products^16,58,59^] can gradually accumulate as the number of irradiations increases. However, when the accumulated pulse number (APN) is further increased, the laser-induced carbonization becomes self-limited once the thickness of the as-generated carbonization products is sufficient to fully absorb the UV laser. At this stage, as shown in the Fig. 2a-v, the upper layer of the as-formed AC gradually transforms into graphene (due to enhanced UV absorption and higher temperatures), while the lower PI layer could also be converted into AC via heat transfer from above^58^. This self-limited carbonization results in a naturally gradient distribution of graphitization degree.

Figure 2b presents the increase in measured IR absorbance of the carbonization products with the APN (laser wavelength of 308 nm, laser fluence of 210 mJ·cm^−^^2^), which clearly exists two distinct stages. In the first stage (i.e., APN ≤ 100), the IR absorbance rises sharply due to the increase of carbonization depth. In the subsequent stage (APN > 100), the absorbance increases slowly with the APN. This might be ascribed to the much slower increase in the newly added carbonization depth in the heat transfer-induced carbonization mode. As presented in Figure S11, the measured carbonization depth exhibits a similar growth trend, which increase rapidly initially and then slowly with the APN. This unique self-limited carbonization mode provides favorable conditions for the formation of TCGC. Figure 2c presents a typical temperature distribution at the glass-PI interface during the self-limited carbonization process. During a single laser irradiation (20 ns), the upper as-formed carbon layer reaches temperatures exceeding 2000 K, sufficient to transform it into graphene. Within several hundreds of nanoseconds after laser irradiation, the heat transfers from the carbonized region to the non-carbonized region, maintaining temperatures high enough to induce carbonization reactions in adjacent PI layers. Essentially, the UV absorption of as-formed products and subsequent downward heat transfer, combined with the stepwise carbonization process from AC to Gr^58^, enable the formation of TCGC. Therefore, it is feasible to generate TCGC with tunable absorbance and material distribution merely by continuous high-fluence UV laser irradiations. Figure 2d presents the variation of the material graded distributions along the thickness through layer-by-layer Raman spectral analysis of TCGC (prepared by the UV laser with an energy of 210 mJ·cm^−^^2^ and 1000 irradiations). The results indicate that the 2D peaks of graphene are present only in the top three layers, and there is an increase in the I_D_/I_G_ [an indicator of structural disorder^54^] from 0.75 at the top layer to 1.52 at the bottom layer (see Figure S12 for details). Concurrently, Figure S13 presents the Raman spectroscopy analysis for the cross-sectional TCGC sample, which are consistent with the above findings. Thus, the prepared TCGC is a kind of functional graded materials, that is, their constituents show a continuous change (from crystalline features to amorphous crystalline forms), inducing a gradient decrease in their thermal conductivity along the thickness. Notably, the structural integrity of the TCGC layer following repeated use is essential for maintaining its light-absorption properties and overall stability. Finite element analysis (FEA) reveals that the TCGC layer could attain a maximum temperature of 300 °C under IR laser irradiation (0.5-3 W), which is well below the thermal degradation threshold of 600 °C^60^ for carbon-based materials (Figure S14). Accordingly, the TCGC layer was subjected to repeated thermal cycling (50–300 °C) and IR laser irradiation (0.5–3 W), followed by absorbance measurements and surface observations. As demonstrated in Figures S15 and S16, no significant changes in the optical properties or surface morphology were observed, thereby confirming the structural stability and durability of TCGC under cyclic utilization.

During the laser carbonization process, gas products are also generated, which can escape from the interface after ~12 h, allowing the carbonized PI film to remain flat and facilitating the subsequent integration of the PDMS adhesion layer. The structure and the geometric parameters (Fig. 2a-iii) of the microcavity array at the bottom of the PDMS layer have been optimized. Firstly, to maximize the self-alignment capabilities, a multiple-to-one design (multiple cavities correspond to one chip) is employed. Compared with the one-to-one design (each cavity corresponds to a single chip), the multiple-to-one design can further mitigate the risk of asynchronous release of chip caused by the uneven temperature distribution. As illustrated in Fig. 2e, a series of transfer precision comparisons were conducted (chip size: 600 µm × 600 µm × 100 µm), using stamps with different cavity sizes. The statistic results indicate that a smaller cavity width (*W*) improves chip transfer accuracy. Secondly, the cavity ratio (*W*/*L*) in the stamp is optimized. As the *W*/*L* increases, the gas expansion within the cavities due to the rise in temperature becomes more pronounced, which is reflected in the simulated interfacial crack tip energy release rate (ERR) under 0.5 W IR laser irradiation for 60 ms (Fig. 2f). However, a high cavity ratio is unfavorable to the picking-up process. Experimental results presented in Fig. 2f have demonstrated that the stamp adhesion initially rises and then falls with the cavity ratio. The stamp adhesion is collectively provided by the adhesion of the PDMS layer and the negative pressure of the cavity. Therefore, increasing the cavity ratio enhances the negative pressure effect but reduces the adhesion of PDMS. Based on these findings, a suitable cavity ratio (*W*/*L* = 2/3) is selected, resulting in a satisfactory adhesion of 37.96 kPa. Finally, the height-to-width ratio (*H*/*W*) of the cavities is further optimized to realize an enhanced temperature-controlled adhesion switchability. As shown in Fig. 2g, as the *H*/*W* increases, the stamp adhesion remains stable, while the thrust force (i.e., the chip release capability of the stamp; see Figure S17 for the testing procedure) gradually increases, attributed to the enlarged gas volume within the cavities. Consequently, the optimal microcavity dimensions are chosen as *W* = 20 µm, *W*/*L* = 66.67%, and *H*/*W* = 1.32. The fabricated stamps exhibit well thermally controlled adhesion switching capability. To further evaluate the durability of these dimension-optimized stamps following repeated use, adhesion strength tests over multiple thermal cycles have been conducted, along with morphological characterization before and after repeated transfer. As shown in Figure S18, both the high and low adhesion strengths exhibited negligible changes following 500 thermal cycles, which indicates that the thermal controlled stamp maintains consistent and reliable adhesion performance. Moreover, after repeated chip transfer cycles, no residual contaminants or surface damage were detected on the stamp (Figure S19), demonstrating the excellent repeatability and reversibility of the SALT technique.

### Self-alignment mechanism through directional photothermal regulation

**Fig. 3:**
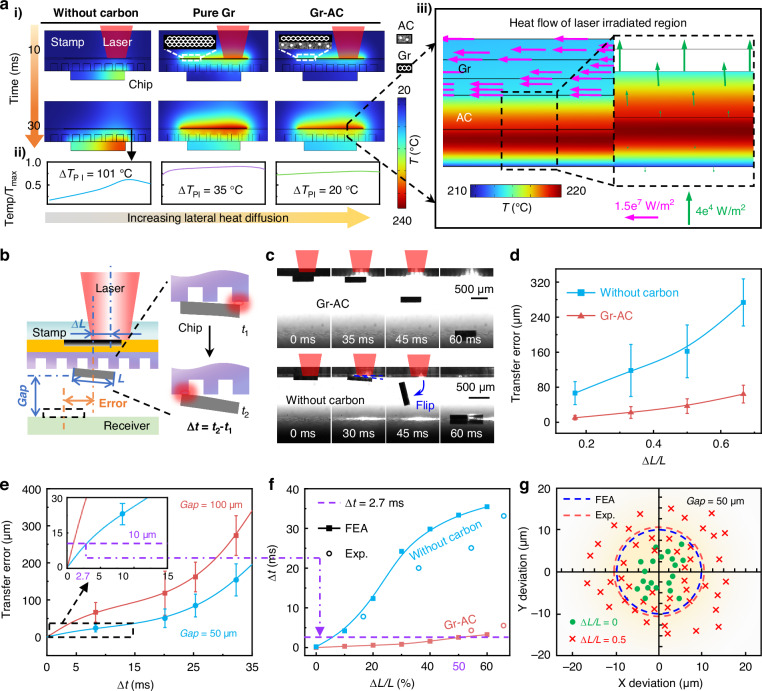
Self-alignment mechanism through directional photothermal regulation. **a** Simulated temperature field of stamps during misaligned IR laser irradiation for three different photothermal conversion layers: without carbon (by chip absorption), pure Gr, Gr-AC. (i) Temperature distribution at 10 ms and 30 ms. (ii) Relative temperature distribution along the horizontal coordinate at the bottom of PI layer under 30-ms IR irradiation. (iii) Heat flow distribution in the laser irradiated region of Gr-AC structure at 30 ms. **b** Schematic diagram of the chip transfer process with laser offset irradiation. **c** Snapshots of delamination of chips and stamps (TCGC and without carbon) under misaligned IR laser irradiation. **d** Statistical results of transfer error of chips with different laser offset degrees (***∆****L/L*). **e** Correlation between delamination time difference (*∆t*) and transfer error at different *Gap*s. **f** Simulation and experimental comparison of ***∆****t* at different laser offset degrees (*∆L/L*). **g** Statistical results of chip position deviation under laser offset irradiation at a gap of 50 µm

The fundamental principle of self-alignment lies in the simultaneous IR laser absorption and heat homogenization by TCGC, thereby preventing asynchronous attainment of the release temperature ( ~ 120 °C) within the adhesive cavities. Figure 3a presents the simulated temperature distribution at the stamp-chip interface (chip size: 600 µm × 600 µm × 100 µm) under a misaligned IR laser (power: 0.5 W, diameter: 400 µm, offset distance: 200 µm) with a duration of 30 ms. The composition of the laser absorption layer significantly influences the temperature distribution. In the cases of chip absorption (without carbon, as shown in Fig. 3a-i) and common AC (Figure S20a), the maximal temperature difference at the bottom of the PI layer (∆*T*_PI_) is substantial ( > 100 °C) due to misaligned irradiation. For the AC-Gr distribution (gradient structures but in the wrong order), the reduction in temperature difference is limited (∆*T*_PI_ = 80 °C, as shown in Figure S20b). In the case of pure graphene as the photothermal conversion layer, due to its extremely high in-plain thermal conductivity, the temperature difference is reduced (∆*T*_PI_ = 35 °C). Notably, the stamp with the TCGC (Gr-AC) exhibits even more uniform temperature distribution (∆*T*_PI_ = 20 °C) compared to pure graphene. Based on the statistics of the average temperature (*T*_ave_) and the temperature difference of the cavity array (∆*T*) over time (Figure S21), TCGC demonstrates a strong ability to enable rapid heating of the entire cavity array (average temperature reaches 120 °C at an irradiation time of 26 ms) while maintaining a very low temperature difference among the cavities (∆*T* < 10 °C).

In-depth simulations have confirmed both high thermal conductivity and the gradient distribution (Gr-AC) is indispensable for efficient lateral heat homogenization. In the case of pure Gr, the absence of AC as a heat insulation layer results in considerable downward heat conduction in the irradiated area, leading to an inhomogeneous temperature distribution (Figure S22). In contrast, for Gr-AC gradient structure, despite the graphene layer being on top, the highest temperature is located inside the AC layer due to the heat dissipation effect of the quartz substrate (acting as a heat sink). Therefore, as illustrated in Fig. 3a-iii, the Gr layer (acting as thermal interface material, as shown in Figure S3c) in the irradiated region will extract a considerable amount of heat from the AC layer ( ~ 10^4 ^W·m^−^^2^) and rapidly conduct ( ~ 10^7 ^W·m^−^^2^) it laterally to the non-irradiated area (Figure S23), with only a minor portion conducted downward. This ingenious strategy of interface thermal management via intentionally directing the heat into top Gr layer through a gradient decrease in thermal conductivity maximizes the effect of thermal homogenization.

Moreover, infrared camera observations in Movie S1 provide further evidence. For the same quartz-TCGC-PI sample, significant differences in heat homogenization effects are observed when the infrared laser irradiates from the front (through the quartz substrate) versus the back (through the PI substrate), confirming the importance of the correct gradient distribution. In addition, although increasing the thickness of the PDMS adhesive layer (e.g., > 1 mm) can achieve uniform temperature in the cavity array (Figure S24), this approach markedly increases the required laser irradiation time ( > 600 ms) and energy input, which is unacceptable for high transfer efficiency. Therefore, the proposed self-alignment mechanism through TCGC does not compromise transfer efficiency. The response time for the chip peeling is comparable to the previously reported laser-assisted reversible transfer process, as detailed in Table S1.

Figure 3b schematically illustrates the mechanism of chip transfer error under misaligned laser irradiation, where the uneven temperature distribution leads to asynchronous release of the chip with a time difference *Δt*. Under misaligned IR laser irradiation, the TCGC guarantees the temperature of the cavity array reaches the release threshold simultaneously, thus significantly reducing the deviation of the chip flight path. As shown in Fig. 3c, time-resolved images of the chip delamination process under misaligned laser irradiation demonstrate that the TCGC-assisted stamp can prevent significant chip displacement or flipping (see Movie S2 for details), compared to the stamp without TCGC. Statistics of the chip transfer errors under different laser offset degrees (horizontal offset distance *ΔL* normalized by chip length *L*, *ΔL*/*L*) at the gap of 100 µm have been conducted in Fig. 3d. The stamp with TCGC exhibits much low transfer errors compared to others, confirming that alignment errors could not constrain the transfer precision in SALT, whose self-alignment capability is absent in other laser transfer techniques, as demonstrated in Table S1.

Through an in-depth investigation of the relationship between the time difference Δ*t* of chip asynchronous delamination and the transfer error, the self-alignment ability can be quantitatively predicted. As shown in Figure S25, asynchronous delamination can lead to the generation of a horizontal velocity *v*_x_ of the chip, which is the primary cause of transfer error. Based a simplified physical model, the horizontal velocity of the chip can be estimated as:

1 $${v}_{{\rm{x}}}=\frac{{\mathrm{9g}}^{2}}{16L}\Delta {t}^{3}$$

(see Note S1 for details), where *g* represents the gravitational acceleration and *L* is the chip size. High-speed camera observations further confirm that, under a fixed gap, the transfer error of the chip is positively correlated with the time difference *Δt*, as shown in Fig. 3e. Subsequently, the relationship between the *Δt* and the laser offset degree *ΔL*/*L* has been calculated by FEA simulations as shown in Fig. 3f, with computational results agreeing well with experiments. By integrating the two relationships, the chip transfer error under a given gap distance and laser offset degree can be predicted. For example, for a target transfer error of 10 µm, the estimated acceptable delamination time difference is *Δt* = 2.7 ms (see inset of Fig. 3e), under a gap of 50 µm. Accordingly, the acceptable maximum laser offset degree is 50% for TCGC (Fig. 3f). As a contrast, the acceptable laser offset degree is only 5.7% for the non-carbon case. Figure 3g presents the chip transfer error derived from a large number of experiments (*Gap* = 50 µm, *ΔL*/*L* = 50%). The statistical result of average transfer error is 11.1 µm, closely matching the predicted precision (i.e., 10 µm). Besides, for non-offset irradiation (*ΔL*/*L* = 0%), the process exhibits a very high accuracy of 4.6 µm, significantly superior to the previous laser non-contact transfer process^29,55,61–63^, as compared in Table S1. Moreover, when the offset degree of the laser spot is 30%, the transfer accuracy remains below 5 µm according to the prediction model. It is important to note that to reduce the difficulty for time-resolved observations, a large chip was chosen for batch experiments. This self-alignment performance should be even more beneficial for small chips. FEA simulations of a small-size chip (100 µm × 100 µm × 20 µm) under misaligned IR laser irradiation (Figure S26) confirm that the TCGC enables a significantly more uniform temperature distribution within the stamp (Δ*T* < 10 °C), in contrast to the stamp without carbon (Δ*T* > 100 °C). Furthermore, experimental measurements of a commercial laser scanning system equipped with galvanometer mirrors quantified its inherent irradiation deviation at ~8 µm (Figure S1b), which corresponds to 27% (i.e., the laser offset degree *∆L/L*) relative to the dimensions of a conventional MicroLED chip (30 µm × 15 µm)^56^. Consequently, these results confirm that the laser offset degree of 20–30% is commonplace in practice, a range that falls well within the tolerance limits of the SALT technique.

The applicability of the SALT technique was further demonstrated through transfer printing onto three-dimensional (3D) substrates, including cylindrical (3 cm diameter) and spherical (5 cm diameter) surfaces (Figure S27). For such low-curvature substrates (where the ratio of the substrate curvature radius *R* to the chip length *L* is *R/L* > 10), planar stamps are adequate for transferring predefined chip arrays. The self-alignment mechanism of SALT ensures the chip drops along a vertical trajectory, enabling its adaptation to variations in the gap between the stamp and the 3D substrate (see Figure S28). When the incident laser spot is misaligned with the target chip (400 µm × 400 µm) by an offset of 120 µm (*∆L/L* = 30%), the conventional transfer approach (without TCGC) exhibits significantly larger transfer errors compared to the TCGC-assisted approach. These results clearly exhibit the self-alignment capability of SALT and highlight its potential for curved electronic integration^64,65^.

### Selective transfer via periodically grayscale-controlled TCGC

**Fig. 4:**
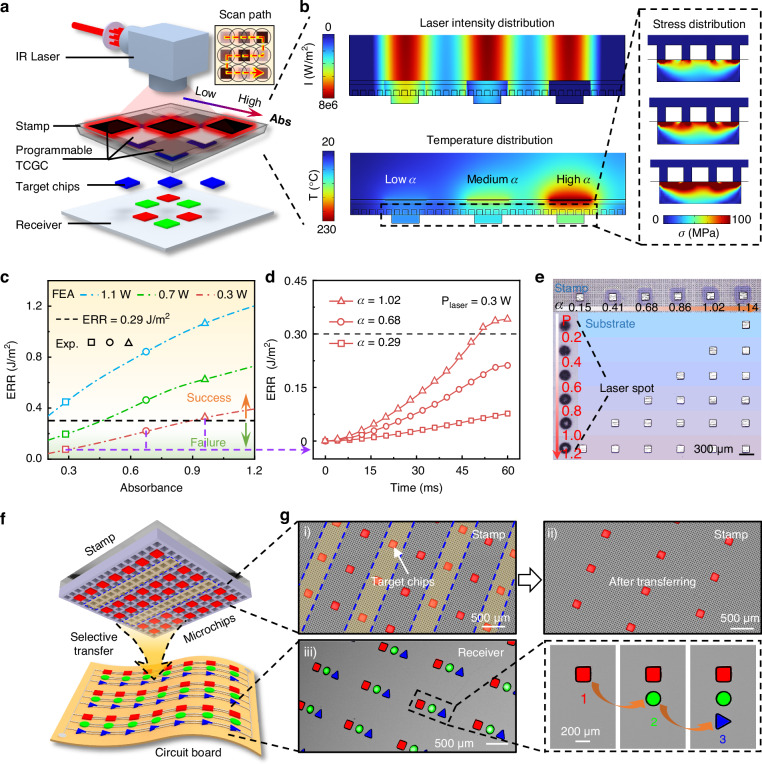
Batch selective transfer via periodically grayscale-controlled TCGC. **a** Schematic diagram of selective transfer of chips via overall IR laser scanning. **b** Laser energy, temperature and stress distributions of a stamp with different absorbance levels (high, medium and low) of TCGC calculated by FEA, under the same power of IR laser irradiation. **c** Relationship between absorbance of TCGC and the stamp-chip interfacial ERR under different power (0.3 W, 0.7 W and 1.1 W) of laser irradiation calculated by FEA. **d** Evolution of interfacial ERR with laser action time of stamps with diverse absorbance levels of TCGC under 0.3W-power IR laser irradiation. **e** Optical images of six types of selective transfer results using stamps with six different TCGC absorbance levels (0.15 to 1.14). **f** Schematic diagram of display circuits assembled by SALT. **g** SEM images of batch selective transfer of three different shapes of microchips (square, circular and triangular) using SALT. (i) Microchips are picked up by a stamp. (ii) The remaining microchips on the stamp after overall IR laser scanning. (iii) Demonstration of integrating three different shapes of chips onto the same PDMS receiver. The right image illustrates the multiple transfer printing process of microchips

Mass transfer with high selectivity and controllability is essential for multiple transfer printing of massive RGB μLED chips into desired layouts. Through selective control the release of microchips, arrays of microchips can be aligned with the customized pitch on the receiver carrier. Periodically arranged TCGC layers with programmable light absorption (i.e., gray scale) can be fabricated by adjusting excimer laser parameters during carbonization. Figure 4a schematically illustrates the overall concept of the batch selective transfer via periodically grayscale-controlled TCGC. An IR laser scans all chips, but only those beneath specific TCGC layers can be released. This eliminates the need for traditional point-by-point laser scanning paths^45^, which can complicate path planning for non-periodic patterns and reduce scanning efficiency due to frequent starts/stops between chip sites. Furthermore, the chip throughput could be significantly enhanced by utilizing a larger laser spot (see Note S2) or flash lamp lift-off technology^66^. Figure 4b shows the working principle of the batch selective transfer through FEA simulations. When three types of TCGC with different IR absorption capabilities are subjected to the same laser irradiation (power of 0.5 W), the TCGC with the highest absorption capacity reaches the release condition first, enabling selective transfer. Thermal camera observations under IR lamp irradiation (Figure S29) confirm significant temperature differences among these TCGC layers, demonstrating their distinct photothermal conversion efficiencies.

Under a specific intensity of IR laser irradiation (determined by power and irradiation time), the heating rate of the cavity array on the stamp surface primarily depends on the IR absorptance of the TCGC. Hence, there exists a critical absorptance enabling the cavity array can just reach the condition for chip release. Figure 4c presents the ERR at the stamp-chip interface under various absorbances and powers of IR laser (all for 60 ms irradiation) through FEA simulations. The critical ERR^67^ is assumed as 0.29 J·m^−^^2^ (see Note S3 for details), and there is a significant difference in the critical absorbance required for different laser powers. For high-power lasers (e.g., 1.1 W), a low absorbance (0.3) is sufficient to achieve chip transfer, whereas for low-power lasers (0.3 W), only a high absorbance (0.9) can enable chip transfer. A series of experimental results (hollow points, shown in Fig. 4c) are in good agreement with the predicted critical absorbance for different laser powers. The essential reason for batch selectivity is that the TCGC layer must ensure that the cavity array reaches the critical temperature (namely ERR) within the limited irradiation time; otherwise, the chip will not be detached from the stamp. For example, as shown in Fig. 4d, under low-power laser irradiation (0.3 W), only the TCGC with high absorbance (i.e., 1.02) can ensure the ERR reaches the release threshold. Consequently, through programmable control the absorbance of the TCGC (up to 1.14, as shown in Fig. 2b), combined with precise regulation of laser power, diverse combinations of selective transfer can be realized. Figure 4e presents six distinct transfer outcomes, which are obtained by combining various absorptances (0.15 ~ 1.14) and laser powers, demonstrating a strong capability for selective transfer printing.

The versatility of batch selective transfer was validated by the multiple transfer printing of microchip arrays with various shapes (square, circular, and triangular, analogy to RGB chips) into desired arrangement. As illustrated in Fig. 4f, the controllability of transfer arrangement for diverse chips could be utilized for RGB full-color layout. Figure 4g presents batch selective transfer printing of square-shaped microchips, where only a portion of chips have been transferred, altering the original pitch in the receiver substrates. By conducting the batch selective transfer printing three times, a horizontal arrangement of three different-shaped microchip arrays on the receiver can be achieved. Additional demonstrations in Figure S32 show that various chip arrangements can be selectively transfer-printed onto the receiver substrate with negligible misalignment. Furthermore, the selective release based on programmable laser absorption could also enhance the process redundancy, particularly crucial for densely packed microchip arrays (e.g., MicroLED for ~μm pitch^56^). In conventional selective transfer techniques, irradiation deviations during high-speed scanning would lead to the undesired printing of adjacent chips^49,68,69^ (Figure S33a). Conversely, even if mis-irradiation hits adjacent chips due to alignment deviations, no mis-transfer will occur in the SALT process (Figure S33b), as the incident laser energy is insufficient to reach the release temperature of the non-target chip.

### Programmable transfer of microchips for flexible full-color display

**Fig. 5:**
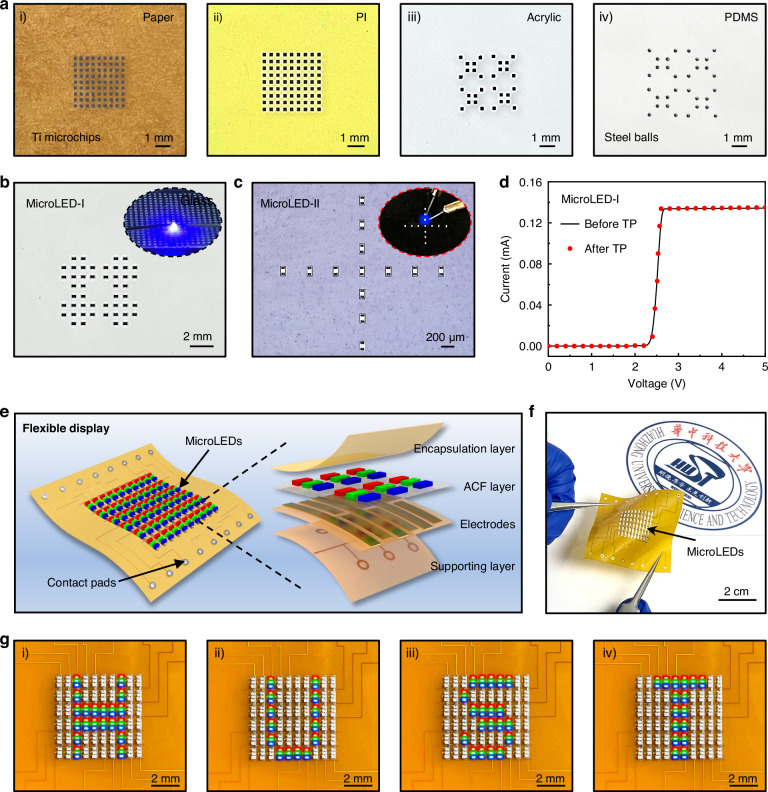
Programmable transfer of microchips for flexible full-color display. **a** Optical images of programmable transfer of titanium chips (200 µm × 200 × 30 µm) and steel balls (200 µm in diameter) on paper, PI, acrylic board and PDMS substrates. **b**, **c** Optical images of selective transfer of 400 µm × 200 µm × 30 µm MicroLEDs (**b**) 160 µm × 80 µm × 20 µm MicroLEDs (**c**). **d** Electrical properties of 400 µm × 200 µm × 30 µm MicroLEDs before and after the transfer process. **e** Exploded schematic diagram of the flexible full-color display. **f** Optical image of the MicroLEDs (400 µm × 200 µm × 30 µm) display. **g** Optical images of the flexible full-color display showing the letters of ‘HUST’

The comprehensive capability of SALT has been demonstrated by selectively transferring microchip arrays with various materials and dimensions onto diverse substrates. Figure 5a shows titanium chips (200 µm × 200 µm × 30 µm) and steel balls (200 µm in diameter) are selectively transfer-printed onto various non-adhesive/adhesive surfaces (i.e., paper, PI, acrylic board, and PDMS) with negligible misalignment. Programmable transfer process of microchips is schematically illustrated in Figure S34 (see Figure S35 for their fabrication details). The microchips are picked up from the donor by a thermal release tape and subsequently released onto the stamp. The IR laser scans the whole stamp, but only microchips beneath specific TCGC layers with predefined patterns are selectively transferred to the receiver. In addition, Figure S36 demonstrates the success transfer of transparent glass chips (600 µm × 600 µm × 100 µm), confirming the compatibility with non-absorptive microchips. In addition, the size limitations of the transferred object have been systematically investigated, the chips of various sizes were successfully transferred by SALT, as shown in Figure S37. Based on a 20 µm microcavity adhesive layer fabricated via current lithography processes, SALT enables the transfer of very small chips with a size of only ~60 µm (Figure S37a). In contrast, for large-chip transfer, a stamp featuring a 3 × 3 array of 6 mm TCGC layer was prepared, as presented in Figure S37b. By defocusing the IR laser to enlarge the spot size, 5-mm chips were successfully transferred under a 3 W laser irradiation. These results indicate the great dimensional compatibility and scalability of SALT, highlighting its potential for handling a broad spectrum of microelectronic components.

The precise and programmable transfer of MicroLEDs is essential for full-color display layout. With the aid of the self-aligned and batch selective transfer printing, flexible MicroLED displays can be integrated efficiently. Figure 5b, c demonstrate the heterogeneous integration of two kinds of MicroLEDs (400 µm × 200 µm × 30 µm and 160 µm × 80 µm × 20 µm) on the PDMS substrate. As shown in Fig. 5d and Figure S38, there was negligible change in the electrical and optical characteristics of the MicroLEDs before and after SALT process. Figure 5e presents a cross-sectional schematic illustration of flexible MicroLED displays fabricated by SALT, which comprises an encapsulation PI layer, RGB MicroLED chips (400 µm × 200 µm × 30 µm), an anisotropic conductive film (ACF) layer, and a PI support layer with electrode interconnects. Here, 18 × 10 RGB MicroLEDs were transfer-printed on an ACF-laminated PI film, followed by a thermo-compressive bonding process (see the details of the fabrication process in Figure S39). Notably, MicroLEDs with a specific color are first picked up integrally from the preparation substrate by the stamp and then selectively transfer through the periodically arranged TCGC, rearranging them into a sparse distribution through three rounds of selective transfer (the transfer process is shown in Figure S40). Thus, the desired RGB layout can be efficiently assembled through multiple selective transfer printing steps. Figure 5f exhibits the fabricated flexible MicroLED display. The display enables individual pixel control through the field-programmable gate array (FPGA), governed by a microcontroller featuring 6-bit scanning and 8-bit data outputs to visualize various alphanumeric characters at high frequencies (specific details are provided in Figures S41 and S42). As illustrated in Fig. 5g, the FPGA-driven device successfully displays the letters “HUST”. Despite these successful demonstrations, the transfer efficiency using a single-laser beam cannot meet the ultrahigh efficiency requirement. Potential improvements include exploring array lasers through beam splitting^48^ by a spatial light modulator (e.g., 10 × 10, 100 chips per shot) for simultaneous multichip processing for faster chip processing could help increase the overall transfer efficiency (possible efficiency > 9000 s^−^^1^, see Note S4 for details). In addition, further optimization of the thermal controlled adhesive layer is needed to enhance reliability for smaller chips.

## Discussion

In summary, a self-aligned laser transfer technique has been developed to achieve high-precision, programmable assembly of microchips onto universal substrates without requiring precise laser-to-die alignment. The key innovation is the use of TCGC, featuring high-heat-conductivity Gr at the top and low-heat-conductivity AC at the bottom. Systematic experimental and simulation studies have revealed the formation mechanism and self-homogenized photothermal conversion process of TCGC. The self-limiting effect of excimer laser confined carbonization causes the as-carbonized products to transform into graphene, which conducts heat downward to induce the carbonization of the lower layer. This gradient carbonized structure naturally forms an efficient heat-homogenizing structure. Under misaligned IR laser irradiation, the upper Gr layer absorbs most heat of the lower AC layer and homogenizes it via rapid lateral conduction, enabling synchronous chip release at all thermal controlled adhesion sites of the stamp, thereby mitigating transfer deviations. Moreover, the periodically arranged, grayscale-controlled TCGC with programmable IR absorption facilitates selective release of the chip even without pre-planned scanning paths. Heterogeneous integration has been achieved by transfer printing micro-objects with diverse shapes and dimensions onto various challenging surfaces, demonstrating reliable adhesion switchability ( ~ 650 times), outstanding size scalability (from 100 μm to 1 mm), and high tolerance for irradiation deviations (transfer accuracy < 5 μm at a laser offset degree of 30%). Demonstrations in programmable transfer printing of RGB MicroLED chips for flexible display illustrate the self-aligned and batch-selective capabilities of SALT, highlighting its immense potential for developing advanced electronic systems.

Despite these advances, several challenges remain to be addressed in the future. First, the SALT’s scalability across different chip sizes requires further development. The transfer of ultrasmall chips ( < 10 µm) could be enabled by employing smaller cavities (via nanoimprint lithography^70^) or alternative adhesive materials (e.g., shape memory polymers^71^). Conversely, in the case of larger chip transfer ( > 20 mm), multiple-spot parallel processing^72^ represents an ideal solution. Second, to further improve transfer accuracy, the parallelism and the gap spacing between chip and receiver must be precise controlled^38,49^. Third, to further expand the applicability of SALT into curved electronics, the introduction of flexible substrates (e.g., ultra-thin glass^73^) into the TCGC-embedded stamp would enhance the conformal capabilities during the transfer process, facilitating the accurate transfer of predefined patterns onto complex, 3D surfaces. Last, to achieve high-throughput assembly of microchips, parallel laser systems (array lasers^16^ or galvanometer-based scanning^9^) could facilitate large-scale and high-output manufacturing.

## Materials and Methods

### Adhesive strength testing

Adhesion strength testing is performed using a Material Testing Systems (model 5944, Instron) shown in Figure S5. During the test, the stamp is first heated to *T*_1_ on a hot plate, followed by applying a 20 N pre-load from the silicon wafer via the press head. After the temperature returns to *T*_0_ (ambient temperature, i.e., 20 °C), the stamp is pulled at a specified speed of 1 mm·s^−^^1^, generating the adhesion force-displacement curve, as shown in Figure S5b. The maximum tensile force (F_max_) is determined from the curve, and the adhesion strength is calculated based on the contact area of the silicon wafer.

### Thrust force testing

For the thrust force test, the stamp and silicon chip are maintained at the ambient temperature (20 °C) before contact. Upon contact, the entire setup is heated to 120 °C using a hot plate. The thrust force is recorded during the temperature rises, resulting in the force-displacement curve shown in Figure S17.

### Fabrication of the stamp

The fabrication process of stamp is schematically illustrated in Figure S9. (i) At first, a non-photosensitive PI precursor (Beijing Biome Technology Co. Ltd., ZKPI-305) is spin-coated onto a quartz glass substrate at 3000 rpm for 40 s, followed by pre-curing on a hot plate at 120 °C for 90 s. After three layers of spin-coating and curing, the PI layer is heated at 220 °C for four hours to achieve a thickness of 15 µm. (ii) Then, a shaped UV laser (wavelength of 308 nm, laser influence of 210 mJ·cm^−^^2^) generated by a laser-transfer platform (iGreatTransfer, see Figure S43) irradiates the PI film through the quartz substrate, forming the TCGC at the PI-glass interface. (iii) The PI layer becomes flat after the gas products escape from the quartz-PI interface. (iv~vii) The PDMS with microcavity array is prepared using a mold created from photoresist. SU8-2025 photoresist (MicroChem, USA) is firstly spin-coated onto a Si substrate at 4000 rpm for 30 s, followed by soft baking (65 °C for 3 min, 95 °C for 6 min), exposure to UV light (365 nm, 10 s), and post-exposure baking (65 °C for 1 min, 95 °C for 5 min). The patterned photoresist mold is then developed using a sequence of isopropyl alcohol (Sigma Aldrich, USA) and propylene glycol methyl ether acetate (MicroChem, USA) for 15 s each until no further white precipitate is observed. Finally, the mold is hard-baked at 135 °C for 20 min. Next, PDMS (DOW Corning Sylgard 184; 10:1 ratio of monomer to cross-linking agent) is spin-coated onto the mold at 2000 rpm for 60 s, followed by curing at 90 °C for 2 h resulting in a thermal controlled adhesive layer with microcavity arrays. (viii) The carbonized PI layer, which has been spin-coated with PDMS at 6000 rpm for 30 s, is bonded with the thermal adhesive layer after plasma treatment using a plasma cleaner (Harrick Scientific, USA). (ix~x) The stamp is formed by hot-pressing the layers together at 90 °C for 2 h.

## Supplementary information

Supporting information for Gradient-graphene-enabled Directional Photothermal Regulation for Self-aligned Laser Transfer Printing

Thermal imaging video of the quartz-TCGC-PI sample irradiated by an infrared laser from the front/back (through the quartz/PI substrate)

High-speed video of the chip transfer process by the stamp with/without TCGC under misaligned infrared laser irradiation

## Data Availability

All study data are included in the article and/or supporting information.
