# Supplementary material for: Gradient-graphene-enabled directional photothermal regulation for self-aligned laser transfer printing
Source: Light Sci Appl. 2026 Jan 12;15:62. doi: 10.1038/s41377-025-02170-9 (PMC12791146; doi:10.1038/s41377-025-02170-9)
Supplement: Supplementary file 1 — Supporting information for Gradient-graphene-enabled Directional Photothermal Regulation for Self-aligned Laser Transfer Printing [file 41377_2025_2170_MOESM1_ESM.docx]

Supporting Information for

# Gradient-graphene-enabled Directional Photothermal Regulation for

**Self-aligned Laser Transfer Printing**

Mengxin Gai^1,2^, Jing Bian^1,2,3, *^, Furong Chen^1,2^, Lei Liu^1,2^, Yu Luo^1,2^, Yuxing Ma^1,2^,

Xincheng Huang^1,2^, Hong Xiao^1,2^, YongAn Huang^1,2, *^

*^1^State Key Laboratory of Intelligent Manufacturing Equipment and Technology, Huazhong University of Science and Technology, Wuhan, 430074, China.*

*^2^Flexible Electronics Research Center, Huazhong University of Science and Technology, Wuhan, 430074, China.*

*^3^School of Microelectronics (School of Integrated Circuits), Nanjing University of Science and Technology, Nanjing 210014, China.*

*^*^**Correspondence: Jing Bian (*[*jsbianjing@njust.edu.cn*](mailto:jsbianjing@njust.edu.cn)*) or YongAn Huang (*[*yahuang@hust.edu.cn*](mailto:yahuang@hust.edu.cn)*)*

**This PDF file includes:**

**Materials and Methods:**

Self-aligned laser transfer experiment.

Layer-by-layer Raman test of TCGC.

Fabrication of microchips.

Fabrication of flexible display.

Characterization and testing details.

Multiphysics simulation.

**Note S1** Theoretical Calculations of Chip Flight Velocity under Misaligned Laser Irradiation.

**Note S2** Comparison of calculations of the chip transfer time between SALT and conventional methods based on pre-planned scanning paths for batch selective transfer.

**Note S3** Experimental and Theoretical Calculations of Critical Energy Release Rate.

**Note S4** Calculation of Chip Transfer Efficiency of SALT via Laser Beam Splitting.

**Fig. S1** Irradiation deviation of laser scanning systems.

**Fig. S2** Impact of non-uniform/misaligned laser irradiation for a stamp with conventional photothermal material.

**Fig. S3** Effect of quartz on the temperature distribution of stamps during 30-ms misaligned IR laser irradiation.

**Fig. S4** Schematic illustration of the pick-up process of the chip using the TCGC-embedded adhesive stamp.

**Fig. S5** A detail description of the adhesion test experiments.

**Fig. S6** Optical images of the TCGC-embedded adhesive stamp with microcavity array.

**Fig. S7** Optical images of TCGC with different sizes and spacings prepared by an ultraviolet excimer.

**Fig. S8** Temperature distribution of the stamp under a 0.45-W IR laser irradiation and the relationship between the power and input current of laser.

**Fig. S9** Schematic illustration of the fabrication process of the stamp.

**Fig. S10** Details of the self-aligned laser transfer experiment.

**Fig. S11** Measured carbonization depth by UV laser ablation of PI.

**Fig. S12** Details of the layer-by-layer Raman test of TCGC.

**Fig. S13** Raman spectral analysis of the cross-sectional TCGC sample.

**Fig. S12** Measured force-displacement curves of thrust force by a Material Testing System.

**Fig. S13** Cycle tests of the TCGC-embedded adhesive stamp for adhesion strength using the Material Testing System.

**Fig. S14** Temperature distribution of a TCGC-embedded stamp and a chip under IR irradiation by FEA.

**Fig. S15** Stability tests of the TCGC layer during repeated use.

**Fig. S16** Stability tests of the TCGC layer under IR laser irradiation at different powers.

**Fig. S17** Measured force-displacement curves of thrust force by a Material Testing System.

**Fig. S18** Cycle tests of the TCGC-embedded adhesive stamp for adhesion strength using the Material Testing System.

**Fig. S19** Morphological observations of the TCGC-embedded stamp before and after repeated chip transfer under a 0.5-W IR laser irradiation.

**Fig. S20** Simulated temperature field of the stamps under 30-ms misaligned laser irradiation for two different photothermal conversion layers: common AC and AC-Gr.

**Fig. S21** Temperature distribution of the stamp with five different photothermal conversion layers: without carbon (by chip absorption), common AC, AC-Gr, pure Gr and Gr-AC.

**Fig. S22** Temperature distribution of a stamp with pure Gr under misaligned laser irradiation was calculated by FEA.

**Fig. S23** Heat flow distribution in the laser non-irradiated region inside the stamp with Gr-AC structure at 30 ms.

**Fig. S24** Effect of heat homogenization for the stamp with common AC as the thickness of the PDMS layer is increased.

**Fig. S25** Asynchronous delamination of the chip from the stamp creates a horizontal velocity that reduces transfer accuracy.

**Fig. S26** Comparison of temperature fields of stamps/small-size chips (100 µm × 100 µm × 20 µm) with common AC and Gr-AC structure under 10-ms laser offset irradiation.

**Fig. S27** Comparison of conventional approach (without TCGC) and SALT for transferring titanium chips (400 µm × 400 µm × 30 µm) onto low-curvature surfaces using planar stamps under IR laser offset irradiation.

**Fig. S28** Comparison of SALT and conventional approach (without TCGC) for transferring titanium chips onto low-curvature surfaces using planar stamps under IR laser offset irradiation.

**Fig. S29** Thermal camera observations of three distinct TCGC layers under IR lamp irradiation.

**Fig. S30** Interfacial adhesion forces between the PDMS stamp and the silicon chip are tested using the Material Testing System.

**Fig. S31** The crack tip energy release rates (ERR) at the chip and stamp interface as a function of laser durations were calculated by FEA under different conditions.

**Fig. S32** Demonstrations of multiple transfer printing of different shapes (square, circular, and triangular) of microchips via SALT.

**Fig. S33** Comparison of conventional approach (pre-planned scanning paths) and SALT in the transfer of densely arranged chips (e.g., MicroLED chips) under laser offset irradiation.

**Fig. S34** Programmable transfer printing process of microchips by SALT.

**Fig. S35** Fabrication process of the microchips.

**Fig. S36** Demonstrations of different chips (silicon and glass chips) transfer-printed onto challenging non-adhesive surfaces by SALT.

**Fig. S37** The investigation of the size limit of the transferred object. a Microchips were transferred by SALT.

**Fig. S38** Electrical and optical characteristics of the MicroLEDs before and after SALT.

**Fig. S39** Fabrication process of the flexible MicroLED display.

**Fig. S40** Optical images of the transfer process of MicroLEDs printed on the FPCB for the flexible display.

**Fig. S41** A programmable MicroLED display is realized by the FPGA hardware.

**Fig. S42** Optical images of the flexible display with a programmable driver circuit.

**Fig. S43** Optical image of a laser transfer printing platform (iGreatTransfer).

**Fig. S44** Comparison of conventional approach (pre-planned scanning paths) and SALT in the batch-selective transfer of MicroLEDs (30 µm× 15 µm).

**Fig. S45** Finite element analysis of the transfer time for MicroLED under a 5-mW IR laser irradiation.

**Fig. S46** Effect of IR laser irradiation for a TCGC embedded adhesive stamp/small-size chip (100 µm × 100 µm × 20 µm).

**Table S1** Statistics of key performance metrics for different laser-driven non-contact transfer techniques.

**Table S2** Comparison of transfer time of MicroLED array across a specific area (300 µm × 300 µm) via conventional approach and SALT.

**Table S3** Important parameters used in this study.

**Movie S1** Thermal imaging video of the quartz-TCGC-PI sample irradiated by an infrared laser from the front/back (through the quartz/PI substrate).

**Movie S2** High-speed video of the chip transfer process by the stamp with/without TCGC under misaligned infrared laser irradiation.

Supporting Information Text

Materials and Methods

**Self-aligned laser transfer experiment.**

The setup for self-aligned laser transfer experiments is illustrated in Fig. S10a. A reference mark is generated by laser ablation of a photographic paper, which is then positioned under the top-view camera and its coordinates recorded. Next, the chip on the stamp is moved beneath the camera, and its coordinates are also recorded. Subsequently, the control script calculates the correct position of the microchips by subtracting the relative distance between the reference mark and the chip center from the stored coordinates of the laser beam focal point. As shown in Fig. S10b, different degrees of laser spot offset relative to the chip are achieved using a motion platform with a positioning accuracy of 0.01 µm. Upon IR laser irradiation, the chip is transfer-printed onto the receiver substrate, and the transfer errors are quantified.

**Layer-by-layer Raman test of TCGC.**

The preparation of samples for layer-by-layer Raman testing is accomplished by using a UV excimer laser (Fig. S12a). Firstly, a high-fluence UV laser (energy of 210 mJ·cm^-2^) ablates the PI film with 1000 APN. Subsequently, the carbonized PI film is peeled from the quartz substrate by a low-fluence UV laser (energy of 84 mJ·cm^-2^) with 30 APN. Adhesive tape is then applied to bond and detach carbonized products in a layer-by-layer manner, with the thickness of each layer being measured using a profilometer (DektakXT, Bruker, USA). The different TCGC layers on adhesive tapes are subsequently analyzed via Raman spectrometer excited by a 532 nm laser (RISE-CLARA, German), as shown in Figs. S12b-f. The results show a gradient distribution of the carbonized products, ranging from graphene at the top to amorphous carbon at the bottom.

**Fabrication of microchips.**

As illustrated in Fig. S35, the fabrication of microchips involves the following steps: (i) A 30 µm-thick titanium film is uniformly attached to the glass substrate. (ii) The metal film is precisely cut by a UV laser cutter (wavelength of 355 nm), and any cutting debris and contaminants on the chip surface are removed by wiping with ethanol-moistened lint-free paper. (iii) A thermal release tape (TRT) is applied to adhere to the metal film, pick up the microchips and form a neatly arranged microchip array on the tape. (iv) The tape, carrying the microchips, is brought into contact with the stamp and heated at 120 °C on a hot plate to ensure reliable transfer of the microchips to the stamp.

**Fabrication of flexible display.**

The process for the fabrication of a flexible display is shown in Fig. S39: (i) MicroLEDs are first transferred from the growth substrate to a thermal release tape (TRT) via UV laser lift-off. (ii) The stamp picks up MicroLEDs from the heated TRT. (iii) Anisotropic conductive films (ACF) are adhered to the flexible printed circuit board (FPCB), followed by heating and pressing (80 °C, 1 MPa) for 1 second using a heating press head. (iv) After aligning the stamp with the FPCB, IR laser scanning of the MicroLEDs is performed. (v) The transferred chips undergo thermo-compressive bonding (180 °C, 50 MPa) for 5 seconds to activate the conductive particles within the ACF. (vi) Finally, the device is encapsulated with a PI layer to form the flexible display.

**Characterization and testing details.**

The absorbance of the PI layer with carbon is measured using a UV-Vis spectrophotometer (1J1-0005, Hitachi, Japan). The surface and interfacial morphology of the microcavity layer is examined via Scanning Electron Microscopy (SEM, SU8020, Hitachi, Japan). Transmission Electron Microscopy (TEM, Tecnai G2 F30, FEI, Netherlands) is employed to inspect the surface morphology of the carbonized layer. Additionally, an ultra-depth three-dimensional microscope (DSX 510, OLYMPUS, Japan) and a high-speed camera (i-speed 508, UK) are utilized to capture the transfer process of the microchips. For monitoring the temperature field distribution during laser irradiation, an infrared thermal imaging camera (Fotric, USA) is used. The optical properties of the MicroLEDs are evaluated using a Fluorescence Spectrophotometer (F-7000, Hitachi, Japan), while their electrical properties are assessed using a probe station (4200-scs, Keithley, USA).

**Multiphysics simulation.**

The simulation of the temperature field and interfacial adhesion during laser transfer printing is conducted using the commercial COMSOL Multiphysics software, which integrates modules for solid heat transfer, solid mechanics, and radiation heat transfer. Specifically, the radiation heat transfer module is utilized to analyze the laser intensity distribution. The solid heat transfer module is used to investigate the internal temperature field distribution within the stamp and chip, while the solid mechanics module focused on analyzing the delamination at the stamp/chip interface and the interfacial energy release rate (ERR). The material parameters employed in this study are summarized in Table S3. The laser transfer printing process is modeled using a two-dimensional (2D) planar configuration. The ambient temperature is set at 20 °C, and the initial pressure within the cavity is assumed to be equal to the atmospheric pressure (101.3 KPa).

Note S1 Theoretical Calculations of Chip Flight Velocity under Misaligned Laser Irradiation

Since the laser spot moves in a single direction (x or y direction), a two-dimensional model was created as shown in Fig. S25. Misalignment between the laser spot and the chip's center results in asynchronous delamination from the stamp, yielding a time difference *∆t*. It is posited that, the chip will rotate around point B on the stamp owing to the gravity, thereby generating a velocity *v*. The equation for the work done by the gravitational moment can be written as:

$\int_{0}^{\theta} mghd\theta=\frac{1}{2}Iw^{2}$ (S1)

Here *m*, *g*, *I* and *w* are the mass, gravitational acceleration, moment of inertia and angular velocity of the chip, *θ* is angle of rotation of the chip. The moment of inertia *I* and angular velocity *w* of chip is:

$I=\frac{1}{3}mL^{2}$ (S2)

$w=\frac{d\theta}{dt}$ (S3)

Where *L* is the chip size. Given that *θ* is small, the sine of *θ* is approximately equal to *θ* itself. It is calculated that *θ* is:

$\theta=\frac{3g}{4L}\Delta t^{2}$ (S4)

The horizontal velocity *v*_x_ of the chip prior to the detachment from the stamp is:

$v_{x}=\frac{L}{2}w\sin\theta=\frac{9g^{2}}{16L}\Delta t^{3}$ (S5)

Note S2 Comparison of calculations of the chip transfer time between SALT and conventional methods based on pre-planned scanning paths for batch selective transfer.

As illustrated schematically in Fig. S44, MicroLED chips measuring 30 µm × 15 µm are arranged in a periodic array within an area of 300 µm × 300 µm. Finite element analysis has shown that a MicroLED chip can be transferred within 10 ms under a 5-mW IR laser irradiation (Fig. S45). For batch selective transfer, conventional methods require serial scanning of all 24 target chips, resulting in a total irradiation time of 240 ms (see Table S2 and Fig. S44). In contrast, the SALT approach employs a large flat-top laser beam (300 µm × 300 µm) shaped by optical elements (e.g., spatial light modulators ^1^). This enables complete coverage of all chips in only single irradiation, totaling 10 ms—representing a 24-fold reduction in total irradiation time of conventional approach. Furthermore, our method eliminates the need for frequent start-stop cycles during the scanning process, thereby significantly enhancing the overall transfer efficiency.

Note S3 Experimental and Theoretical Calculations of Critical Energy Release Rate

Adhesion strength of the stamp and chip was tested (preload force of 20 N, peeling speed of 1 mm·s^-1^), and the results are shown in Fig. S30. The average value of the maximum peeling force *F*_peel_ is 1.629N. The relationship between interfacial peeling force *F*_peel_ and energy release rate *γ* is given by the following equation ^2^:

$\gamma=\frac{F_{peel}^{2}}{6\pi r^{3}E^{*}}$ (S6)

Where *r* is the equivalent radius of a square silicon chip with the in-plane dimension of *a* × *a*, which is given by $r=\sqrt{\frac{a^{2}}{\pi}}$. The Young's modulus *E*^*^ is written as:

$\frac{1}{E^{*}}=\frac{3}{4}\text{（}\frac{\text{1-}\nu_{PDMS}^{2}}{E_{PDMS}}\text{+}\frac{\text{1-}\nu_{Si}^{2}}{E_{Si}}\text{）}$ (S7)

*E*_PDMS_ and *v*_PDMS_ are the Young's modulus and Poisson's ratio of PDMS, respectively. Correspondingly, *E*_Si_ and *v*_Si_ represent the Young's modulus and Poisson's ratio of silicon. Concurrently, the Young's modulus of the silicon chip is significantly higher than that of PDMS, thus resulting in $E^{*}\approx\frac{4E_{PDMS}}{3}$. Finally, the critical energy release rate at the chip and stamp interface is calculated to be 0.29 J·m^-2^.

Note S4 Calculation of Chip Transfer Efficiency of SALT via Laser Beam Splitting

For the transfer of large chip (600 µm × 600 µm × 100 µm): as shown in Fig. S8, the delamination time of the chip under 0.45-W infrared laser irradiation is 32 ms. Based on the above simulations, a single laser beam can transfer 31 chips within 1 second, which is insufficient to meet the ultra-high efficiency requirement. The multichip transfer process can be achieved by using an array of lasers, which is created by beam splitting via a spatial light modulator (SLM). After passing through the SLM, a laser beam is divided into a 10×10 array, ensuring that 100 chips can be irradiated concurrently with a single shot. In summary, self-aligned laser transfer (SALT) can achieve a chip transfer efficiency of 3100 chips per second using the technique of spot beam splitting.

In the case of small chip transfer (size of 100 µm × 100 µm × 20 µm): under a 0.3-W IR laser irradiation, the delamination time of the chip from the stamp is ~11 ms (ERR=0.29 J·m^-2^, as shown in Fig. S46). As with the above calculation process, the transfer efficiency of a small-sized chip can reach 9090 s^-1^ when a spatial light modulator (i.e., 10 × 10, 100 chips per shot) is used for laser beam splitting.


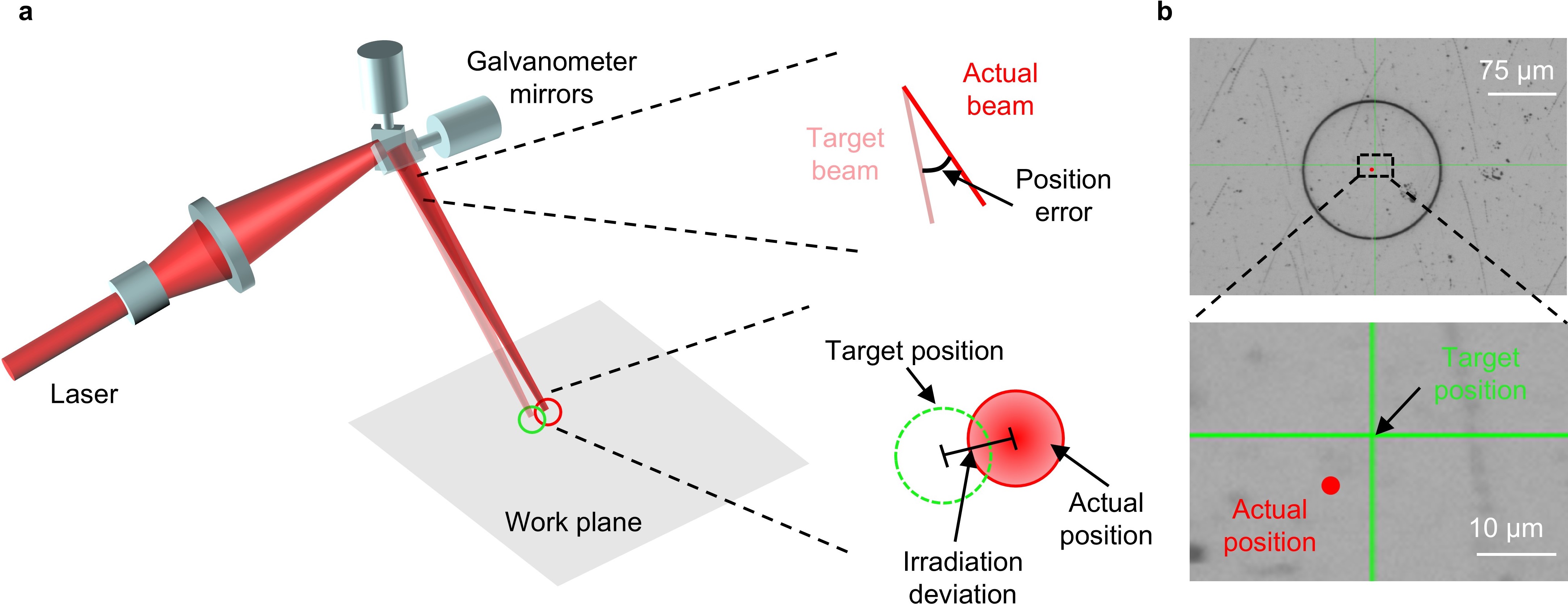


Fig. S1 Irradiation deviation of laser scanning systems. a Schematic illustration of position error of galvanometer mirrors and irradiation deviation (the offset distance between the actual spot position and the target position) in the laser scanning system. b Optical images of measured irradiation deviation (~ 8µm) in the laser scanning system.


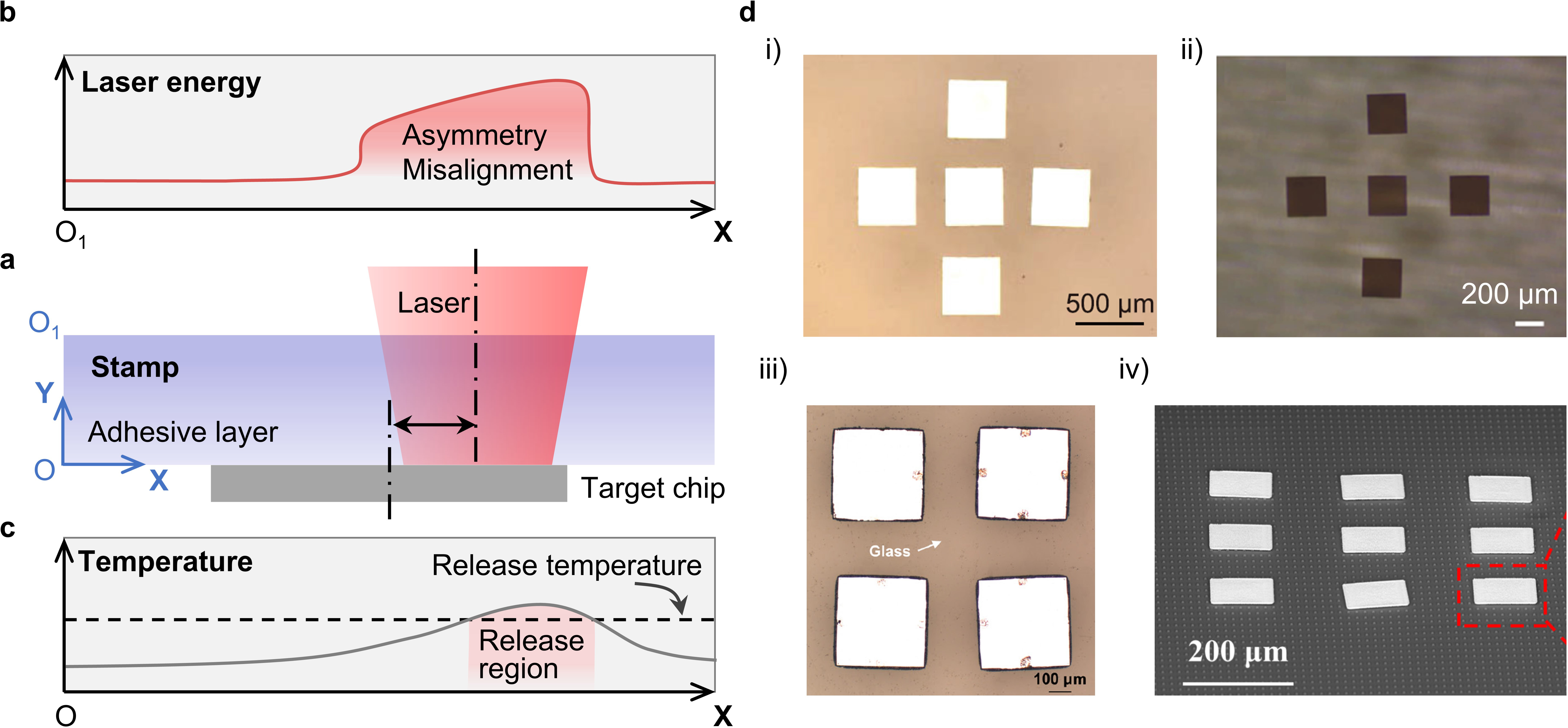


Fig. S2 Impact of non-uniform/misaligned laser irradiation for a stamp in conventional laser transfer techniques. a Schematic illustration of a stamp without TCGC under a non-uniform/misaligned laser irradiation. b Laser energy distribution irradiated on the upper layer of stamp along the x-axis direction. c Temperature profile along the x-axis direction at the bottom of the stamp (a certain adhesive region would reach the chip release temperature, i.e., release region). d Irradiation deviations lead to transfer errors ^3-6^.


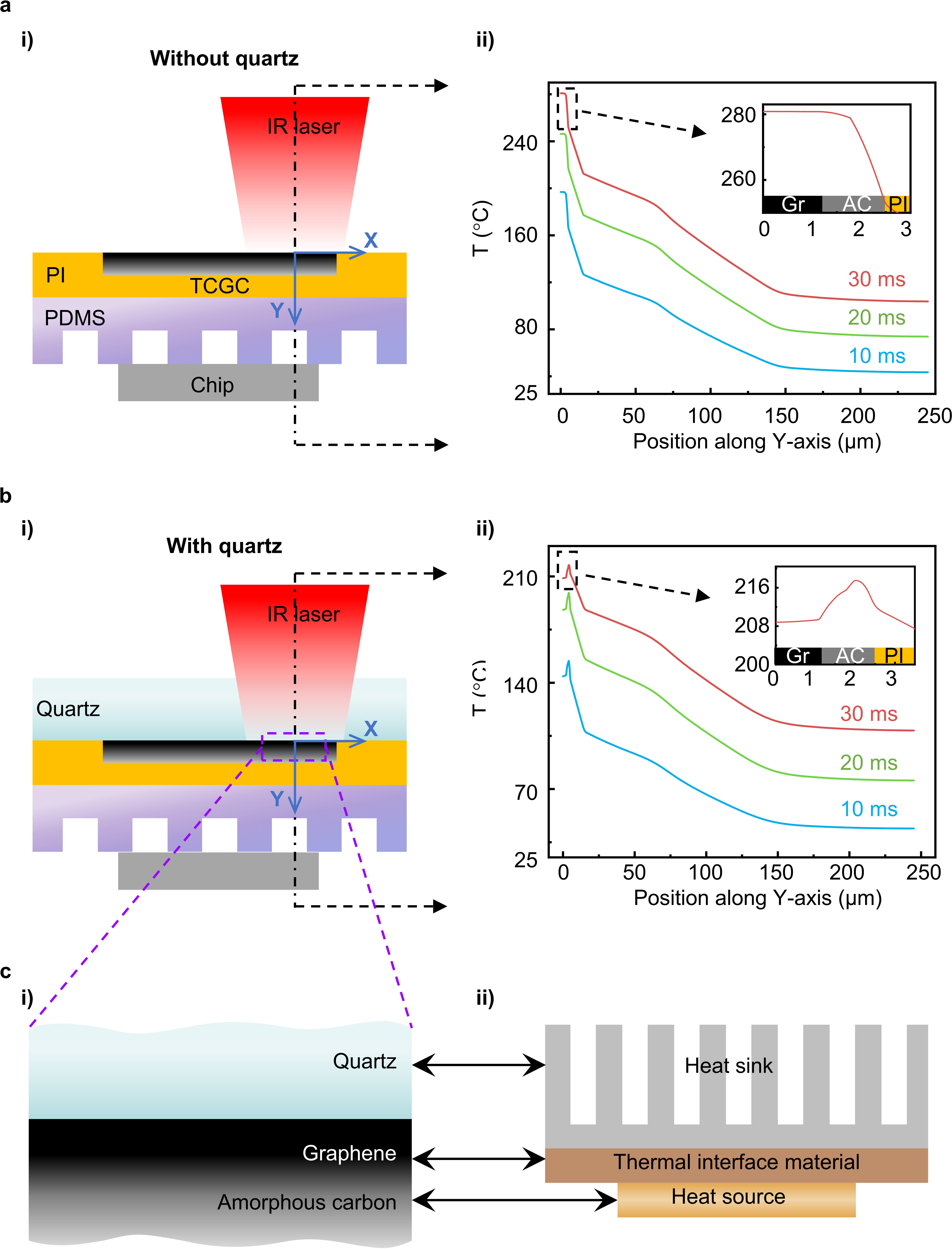


Fig. S3 Effect of quartz on the temperature distribution of stamps during 30-ms misaligned IR laser irradiation. a Schematic illustration of a stamp without quartz: (i) Schematic of the stamp without quartz under the misaligned laser irradiation. (ii) Temperature distribution along the Y-axis direction of the stamp under different IR laser durations (10 ms, 20 ms, 30 ms). The inset shows that the highest temperature is in the Gr layer. b Schematic illustration of a stamp with quartz: (i) Schematic of the stamp with quartz. (ii) Temperature distribution along the Y-axis direction of the stamp under different IR laser durations (10 ms, 20 ms, 30 ms). The inset shows that the highest temperature is located within the AC layer. c Schematic diagram of the TCGC-embedded adhesive stamp and a heat dissipation system: (i) A partial enlargement of the stamp with quartz (i.e., quartz, graphene and amorphous carbon). (ii) Schematic of the heat dissipation system, consisting of a heat source, a thermal interface material and a heat sink.


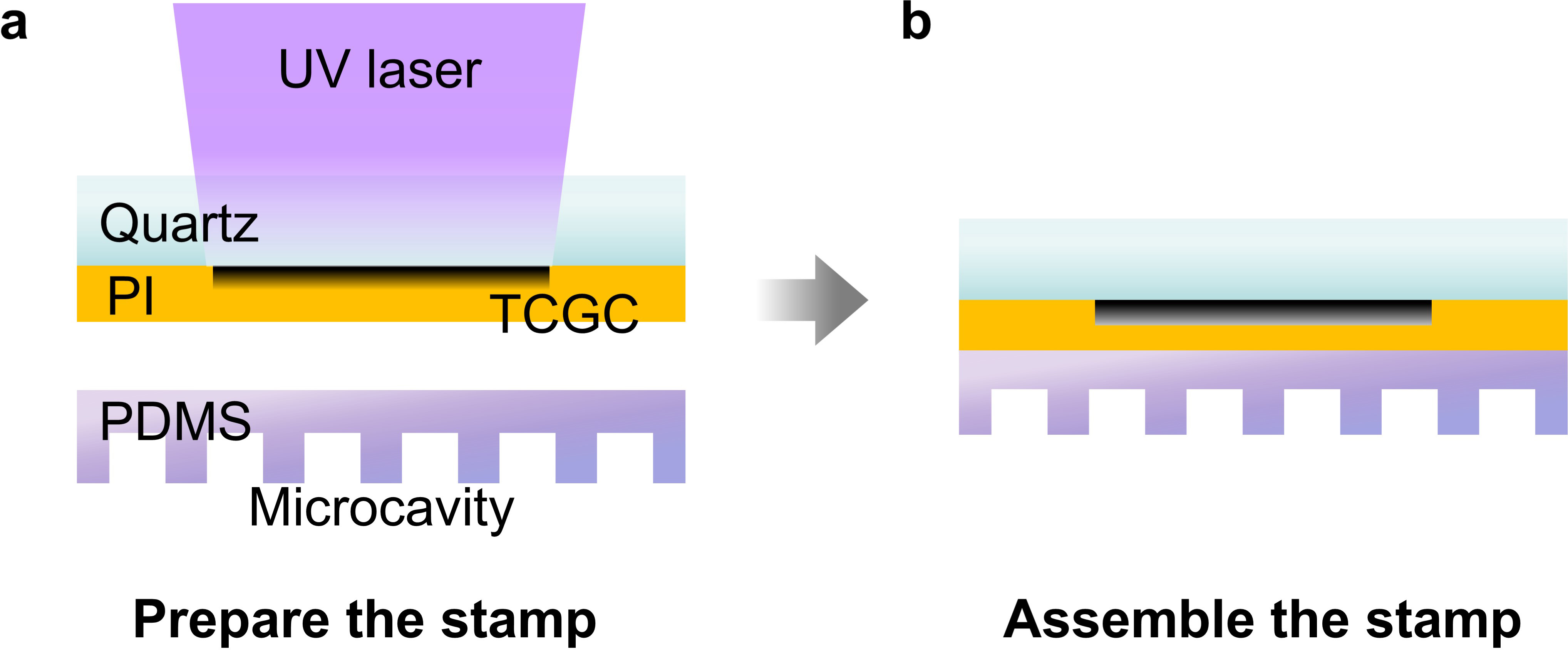


Fig. S4 Schematic illustration of the preparation of the TCGC-embedded adhesive stamp: a TCGC is generated via UV laser irradiation. b Bonding the PI layer to the PDMS adhesive layer to form the stamp.


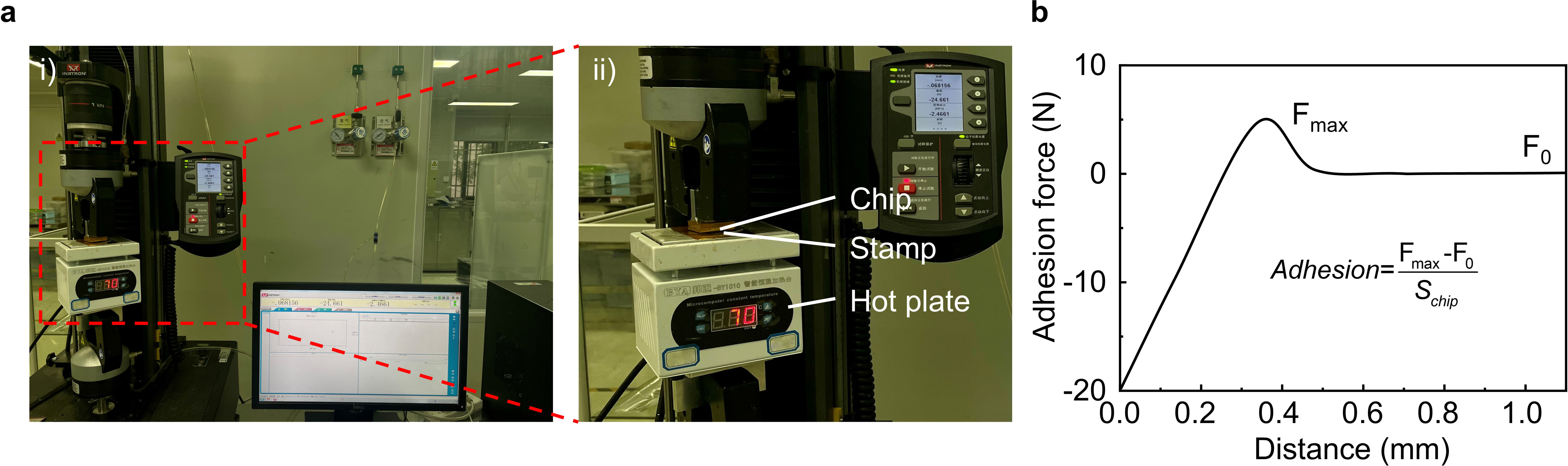


Fig. S5 A detail description of the adhesion test experiments. a Photographs of the home-made pull test equipment consisting of a Materials Testing System (Model 5944, INSTRON), a stamp, a chip and a hot plate. b Measured force-displacement curves using the testing equipment.


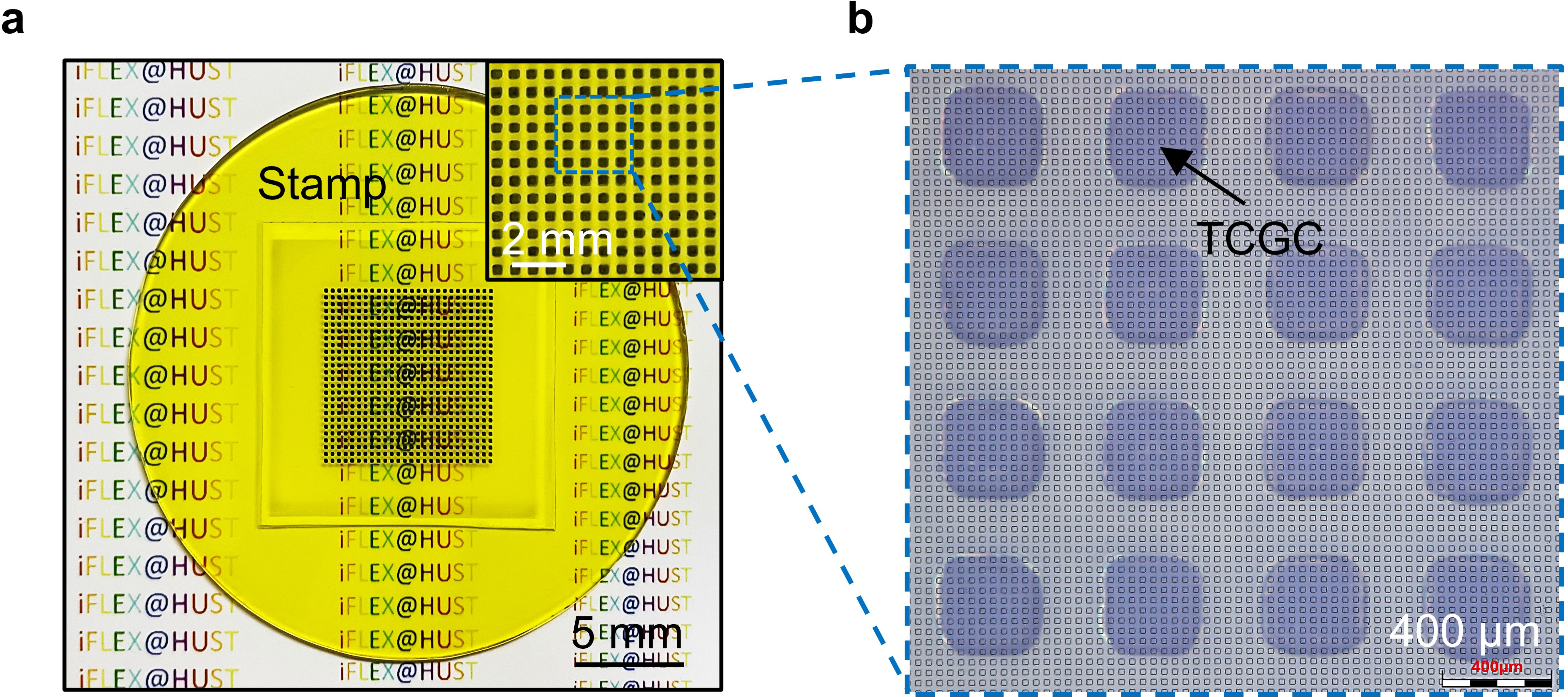


Fig. S6 Optical images of the TCGC-embedded adhesive stamp with microcavity array: a Optical image of a 2-inch stamp with a 27 × 27 array of TCGC. b The enlarged optical image of the stamp with a 20-µm microcavity array.


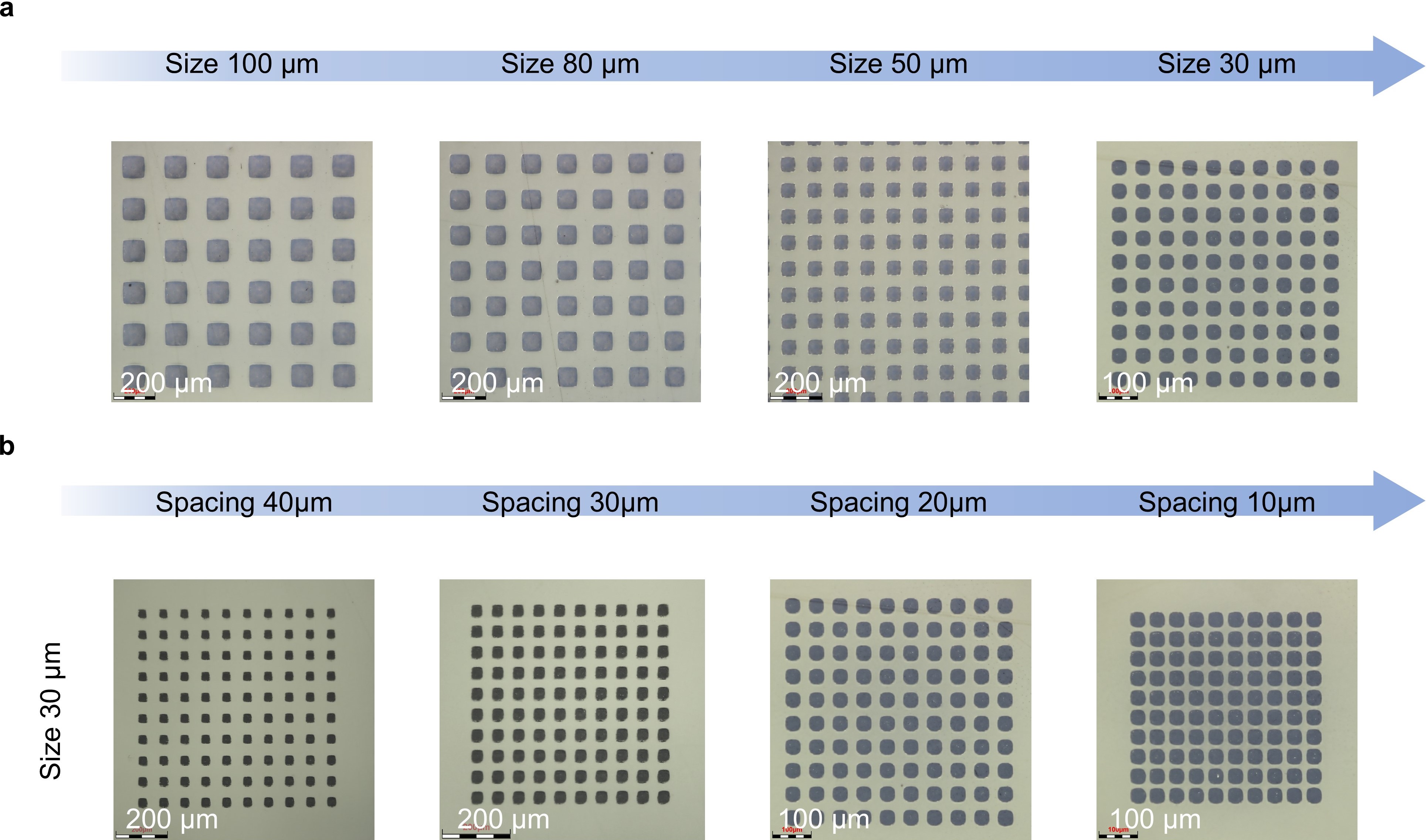


Fig. S7 Optical images of TCGC with different sizes and spacings prepared by an ultraviolet excimer. a Optical images of TCGC with different sizes (30 µm~100 µm). b Optical images TCGC sized 30 µm with different spacings (10 µm ~40 µm).


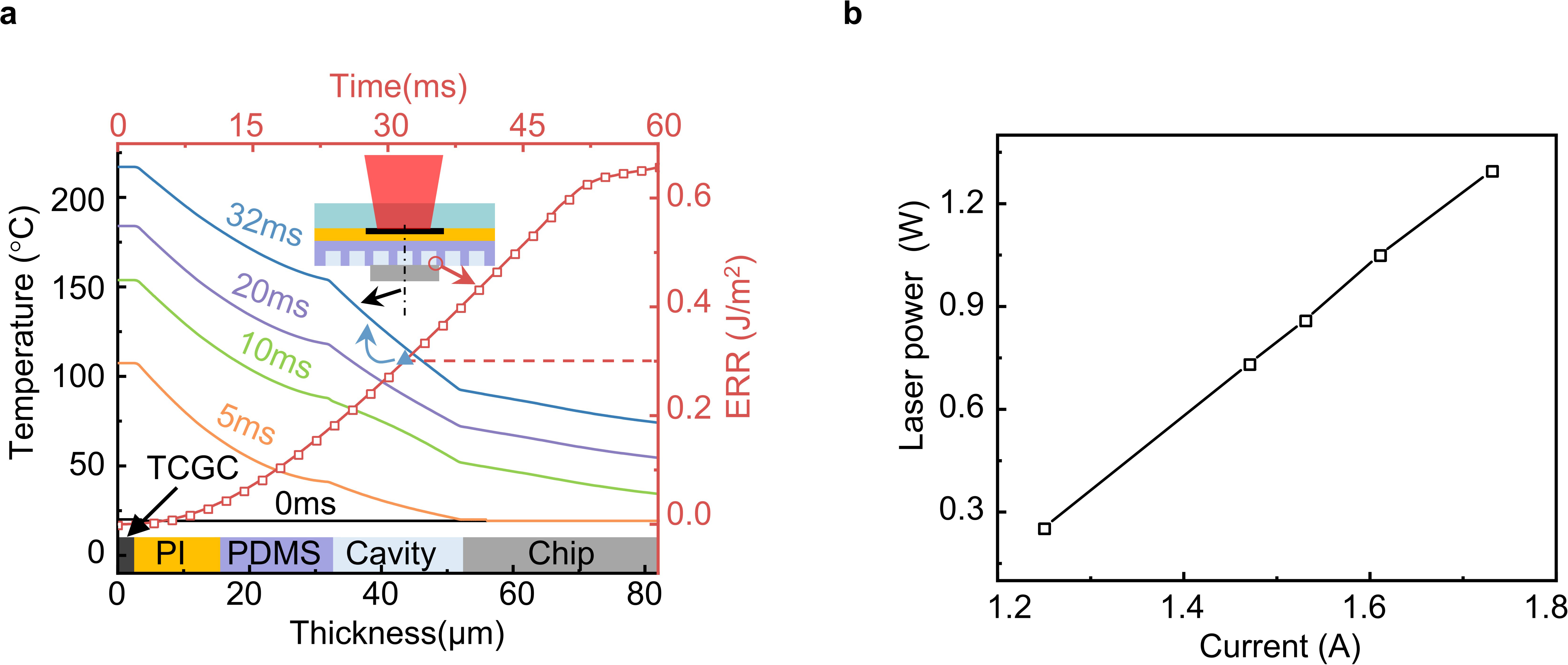


Fig. S8 Temperature distribution of the stamp under a 0.45-W IR laser irradiation and the relationship between the power and input current of laser. a Spatial distribution of the temperature of the stamp and simulated stamp-chip interfacial crack tip energy release rate (ERR) during IR laser irradiation were calculated by FEA. b The laser power depends on the input current of the laser.


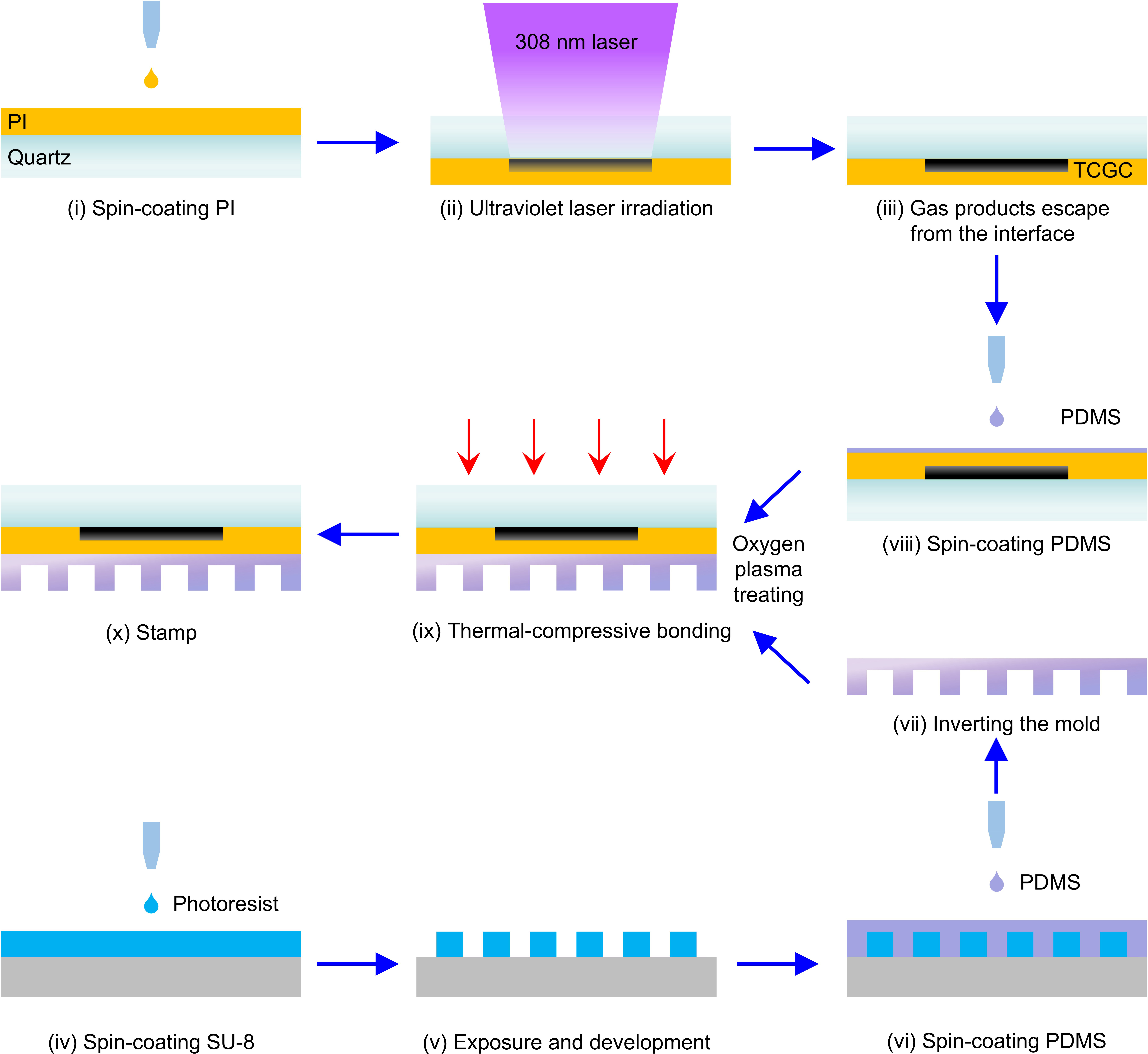


Fig. S9 Schematic illustration of the fabrication process of the stamp. (i) Spin-coating a PI layer on a quartz glass. (ii) A shaped UV laser irradiates at the glass-PI interface to form the TCGC. (iii) The PI film flattens after the gas products escape. (iv) Spin-coating SU-8 photoresist on a silicon substrate. (v) UV light exposure and development of photoresists. (vi) Spin-coating a PDMS layer on the photoresist mold. (vii) Demold the PDMS layer with microcavities. (viii) Spin-coating a PDMS layer on the carbonized PI layer. (ix) Assembly the stamp and hot pressing it. (x) The completed stamp is prepared.


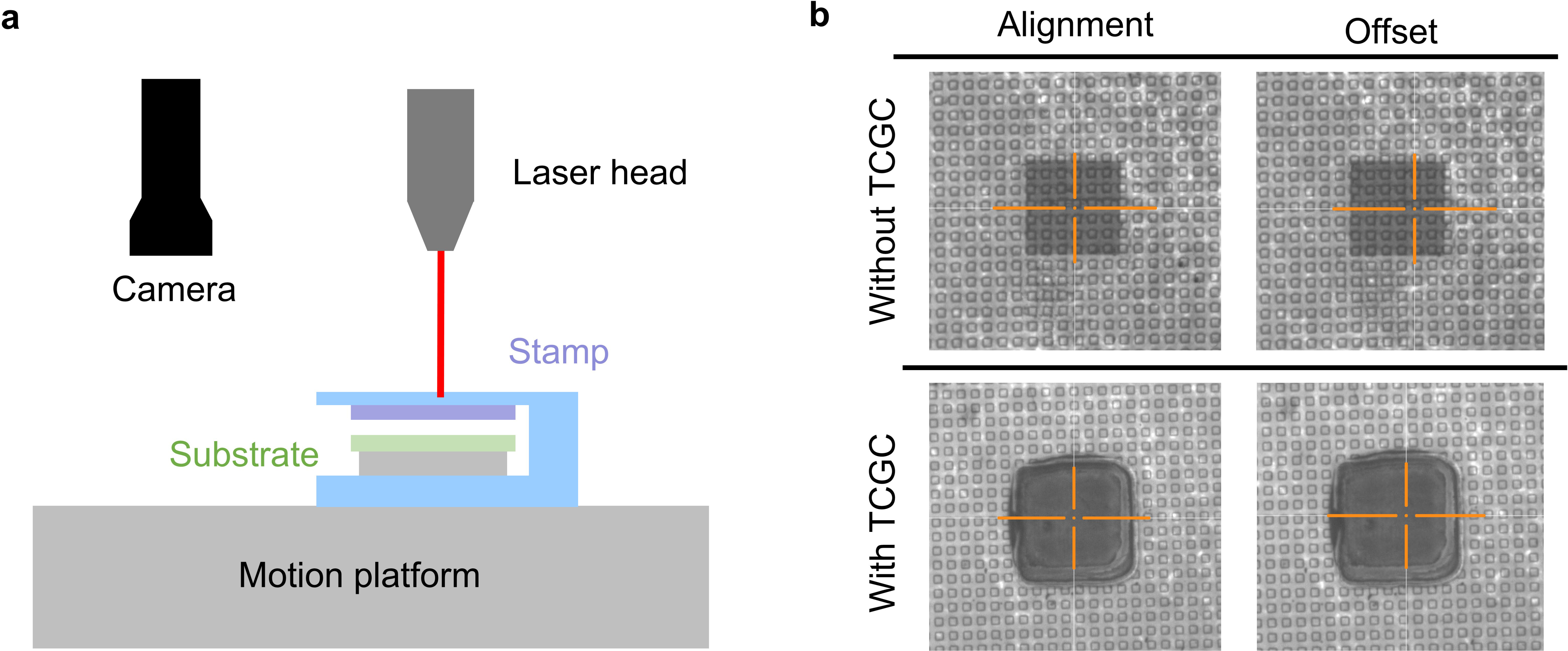


Fig. S10 Details of the self-aligned laser transfer experiment. a Schematic illustration of the self-aligned laser transfer experiment. b Optical images of the laser spot in alignment/offset with the chip (for the stamp without TCGC), TCGC (for the TCGC embedded adhesive stamp).


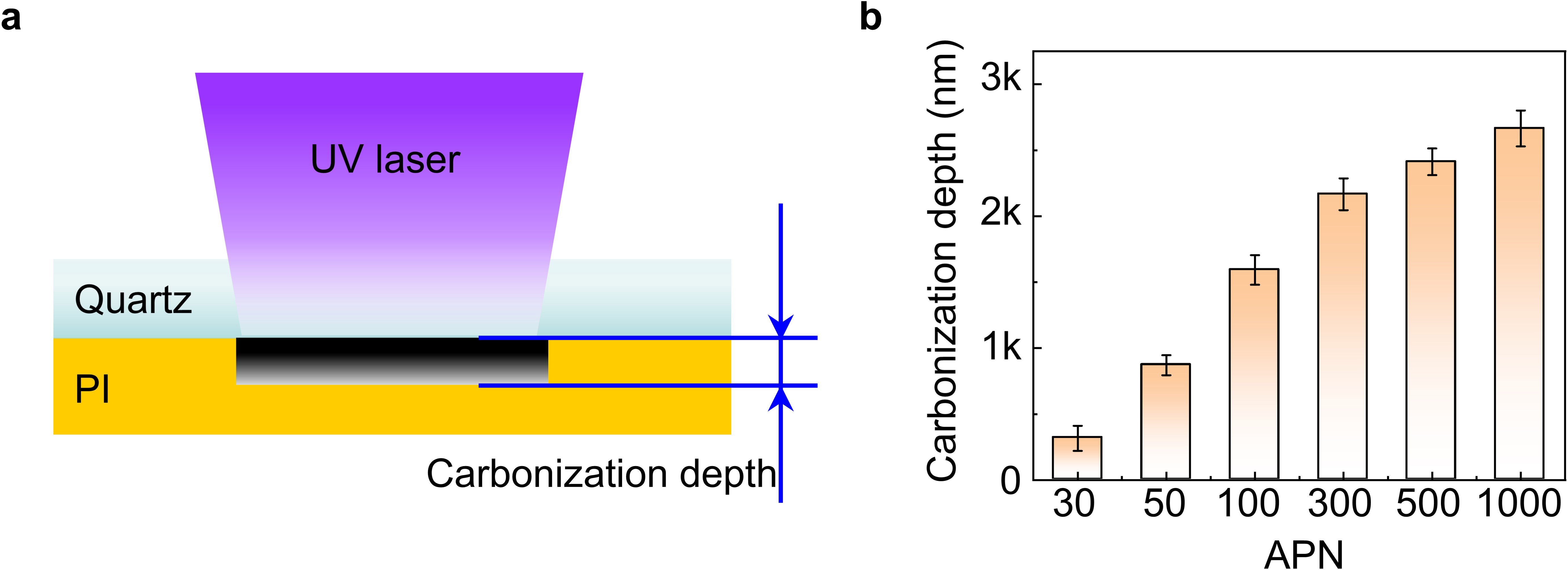


Fig. S11 Measured carbonization depth by UV laser ablation of PI. a Schematic illustration of the laser ablation of PI. b Carbonization depth of PI under different APN (30~1000) of UV laser.


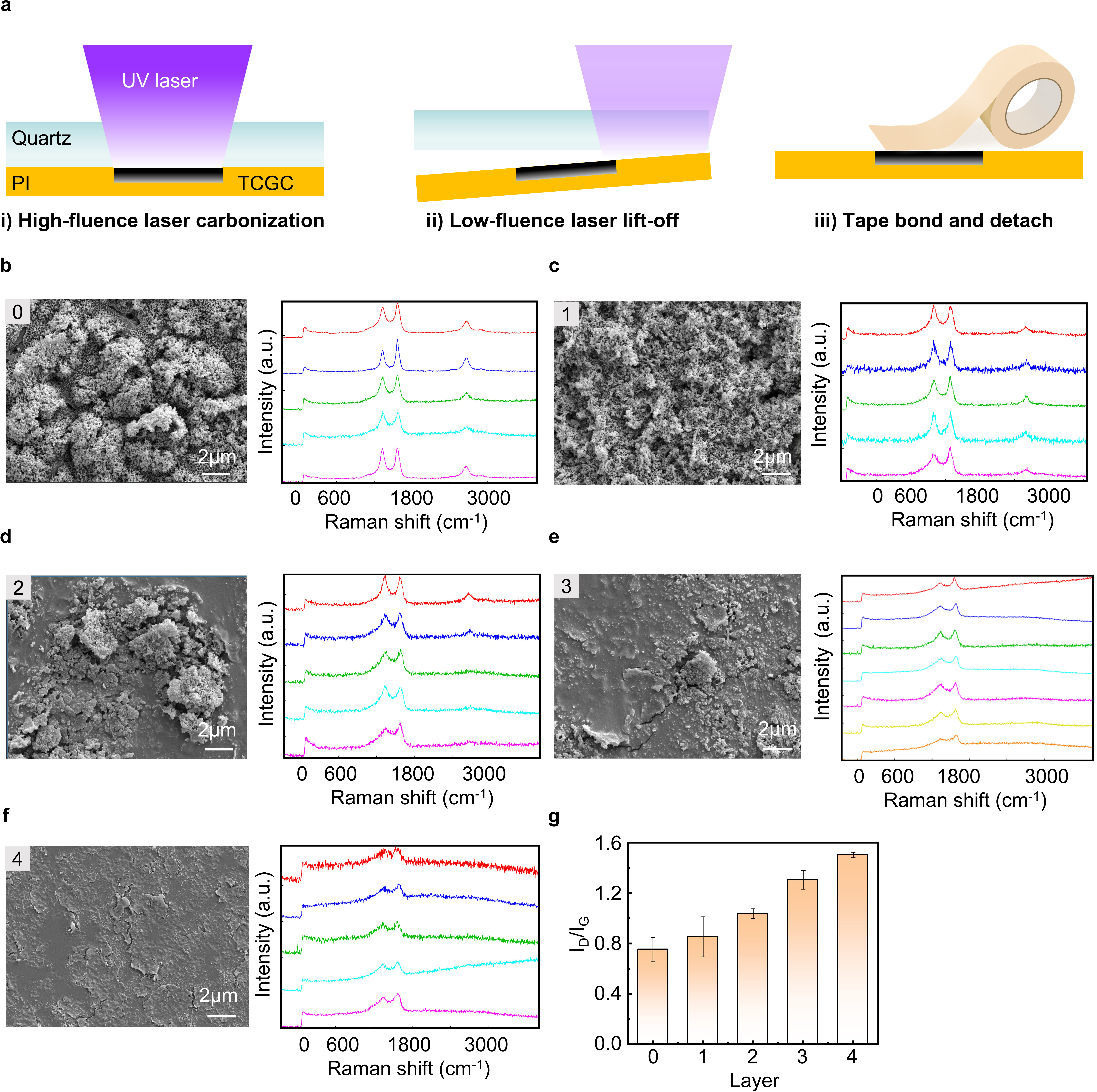


Fig. S12 Details of the layer-by-layer Raman test of TCGC. a Schematic illustration of the preparation and peeling process of the carbonized PI film using a UV laser. (i) A high-fluence UV laser (energy of 210 mJ·cm^-2^) ablates the PI film with 1000 APN. (ii) The carbonized PI film is peeled from the quartz substrate by a low-fluence UV laser (energy of 84 mJ·cm^-2^) with 30 APN. (iii) Adhesive tape is applied to bond TCGC and detach in a layer-by-layer manner. b-f SEM images and Raman test spectra of different layers of TCGC. g I_D_/I_G_ (an indicator of structural disorder) of different TCGC layers.


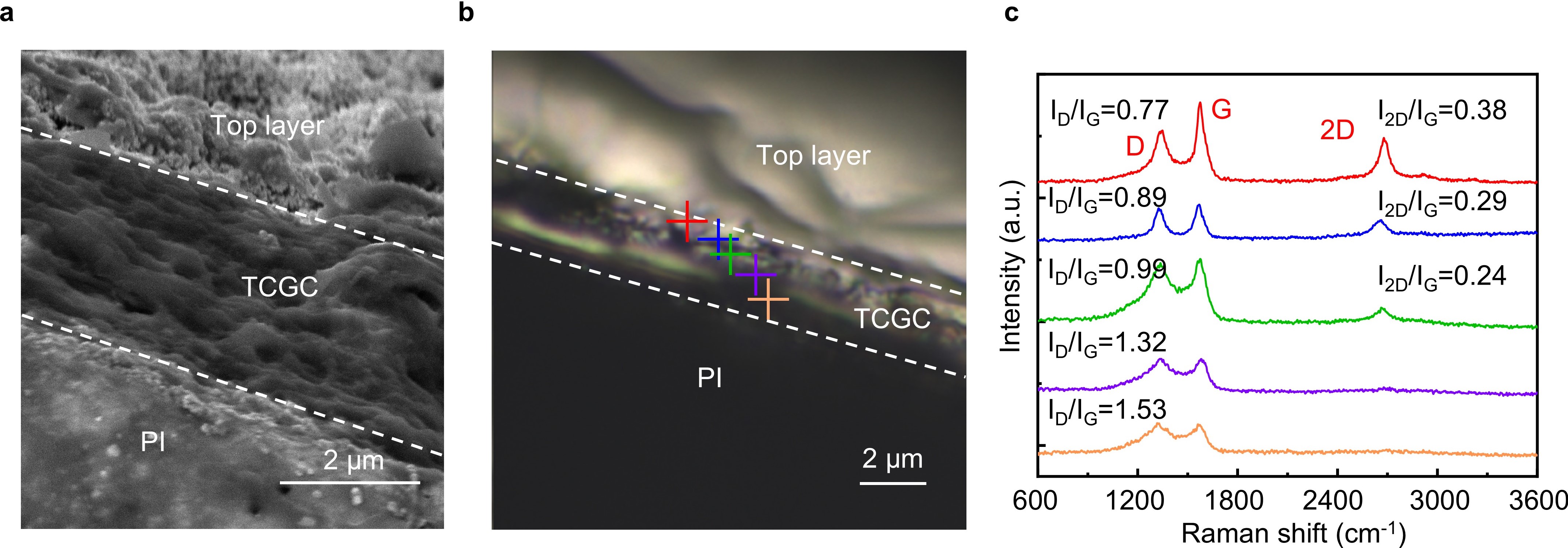


Fig. S13 Raman spectral analysis of the cross-sectional TCGC sample. a SEM image of cross-sectional TCGC sample. b Optical image of cross-sectional TCGC sample. c Raman spectral analysis of TCGC along the thickness.


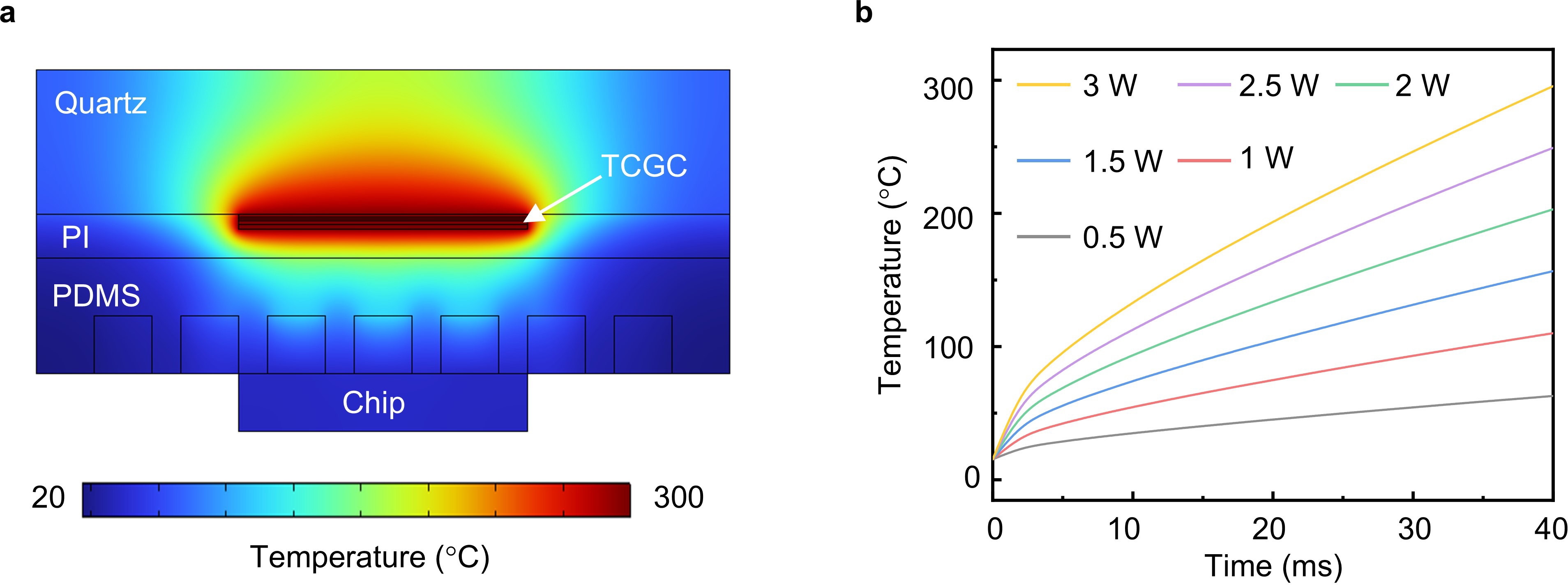


Fig. S14 Temperature distribution of a TCGC-embedded stamp and a chip under IR irradiation by FEA. a Simulated temperature field of the stamp and chip under a 3-W IR laser irradiation at 30 ms. b Maximum temperature of TCGC under laser irradiation at different power levels (0.5-3 W).


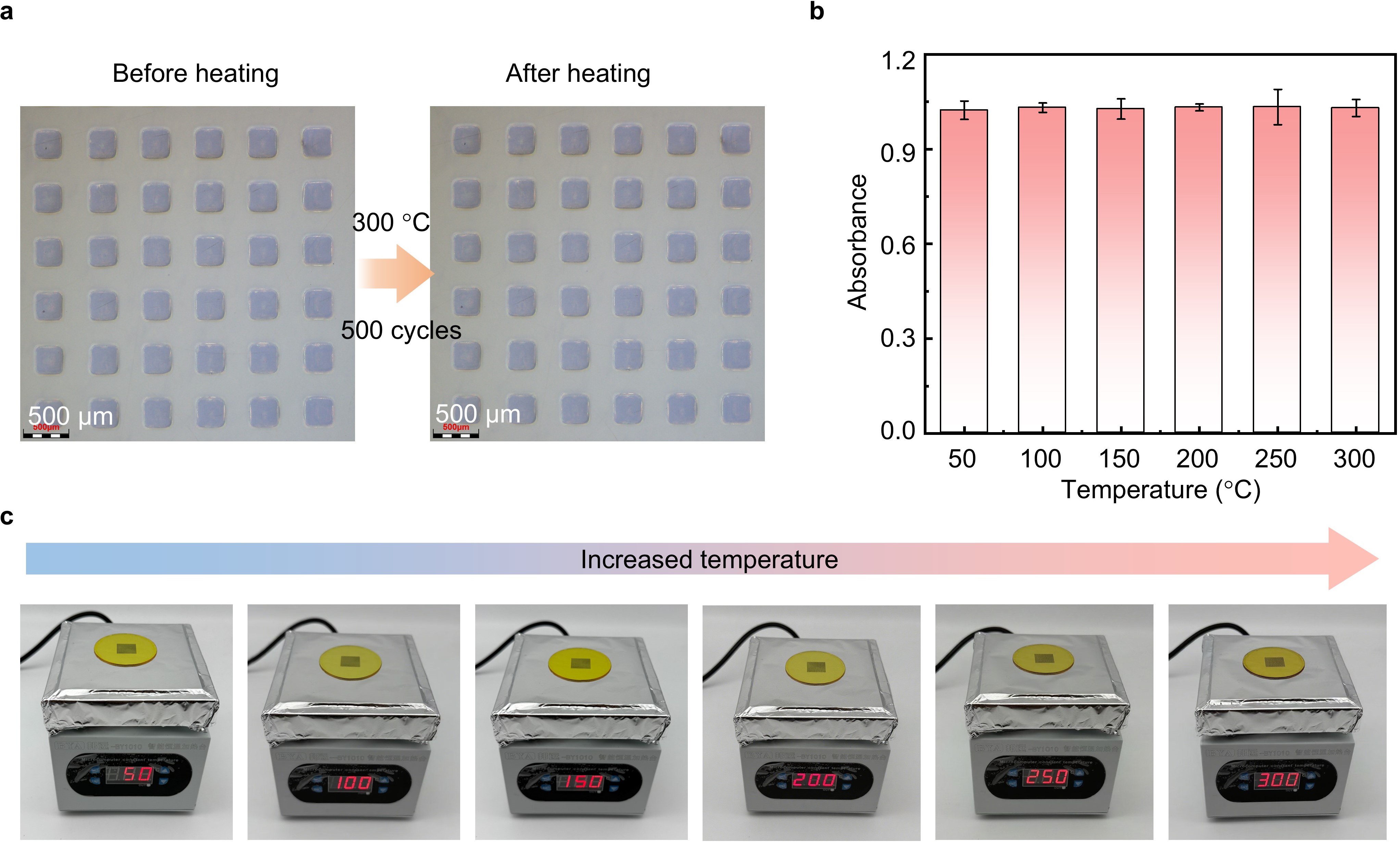


Fig. S15 Stability tests of the TCGC layer during repeated use. a Optical images of the TCGC layer within the stamp before and after heating to 300 °C. b Absorbance testing of the TCGC layer under thermal cycling at different temperatures. c Optical images of the TCGC layer at different heating temperatures.


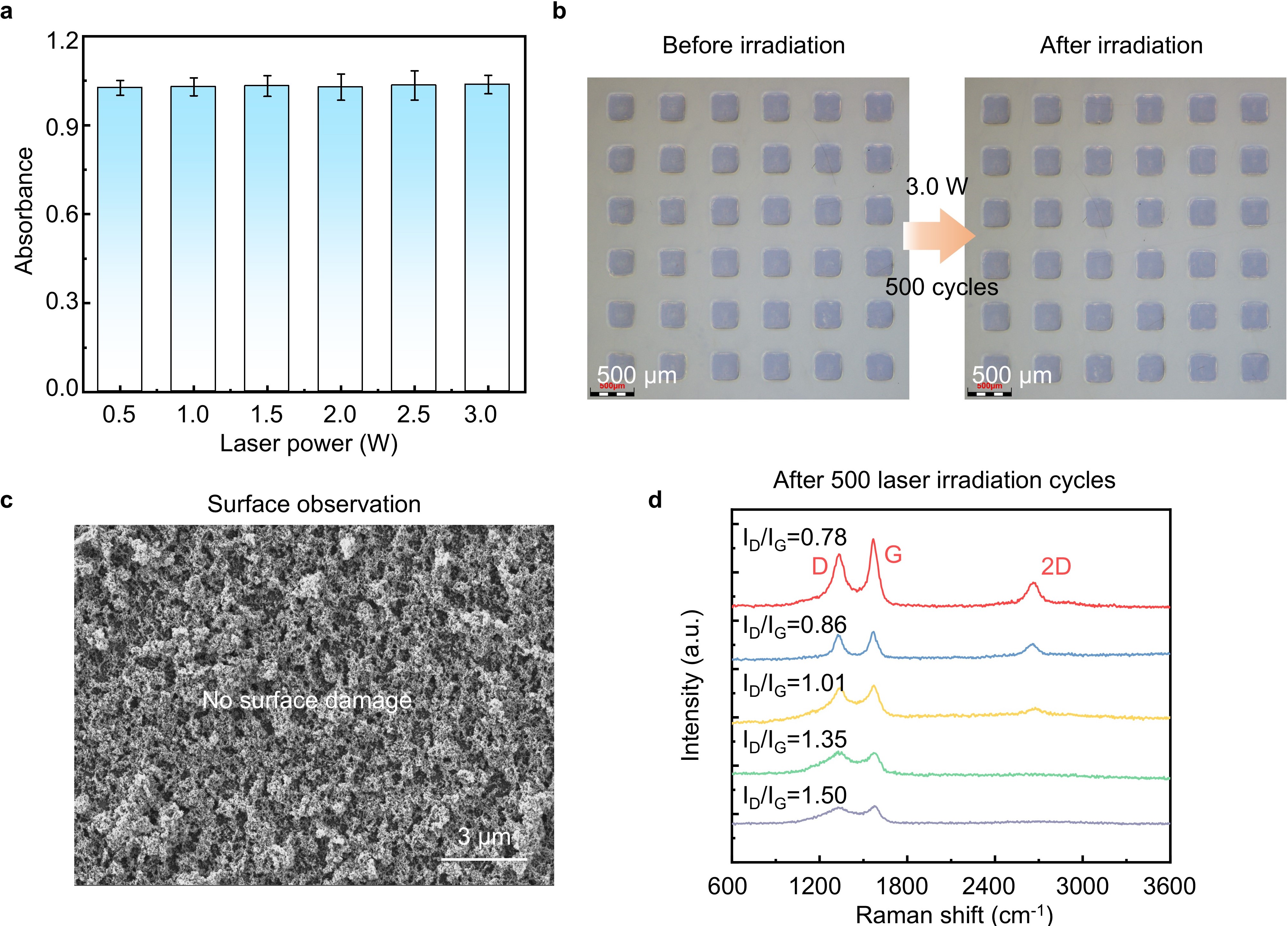


Fig. S16 Stability tests of the TCGC layer under IR laser irradiation at different powers. a Absorbance testing of the TCGC layer under IR laser irradiation at different powers (0.5-3 W). b Optical images of the TCGC layer before and after 500 cycles of 3-W IR laser irradiation. c SEM image of the top TCGC layer. d layer-by-layer Raman spectral analysis of the TCGC layer.


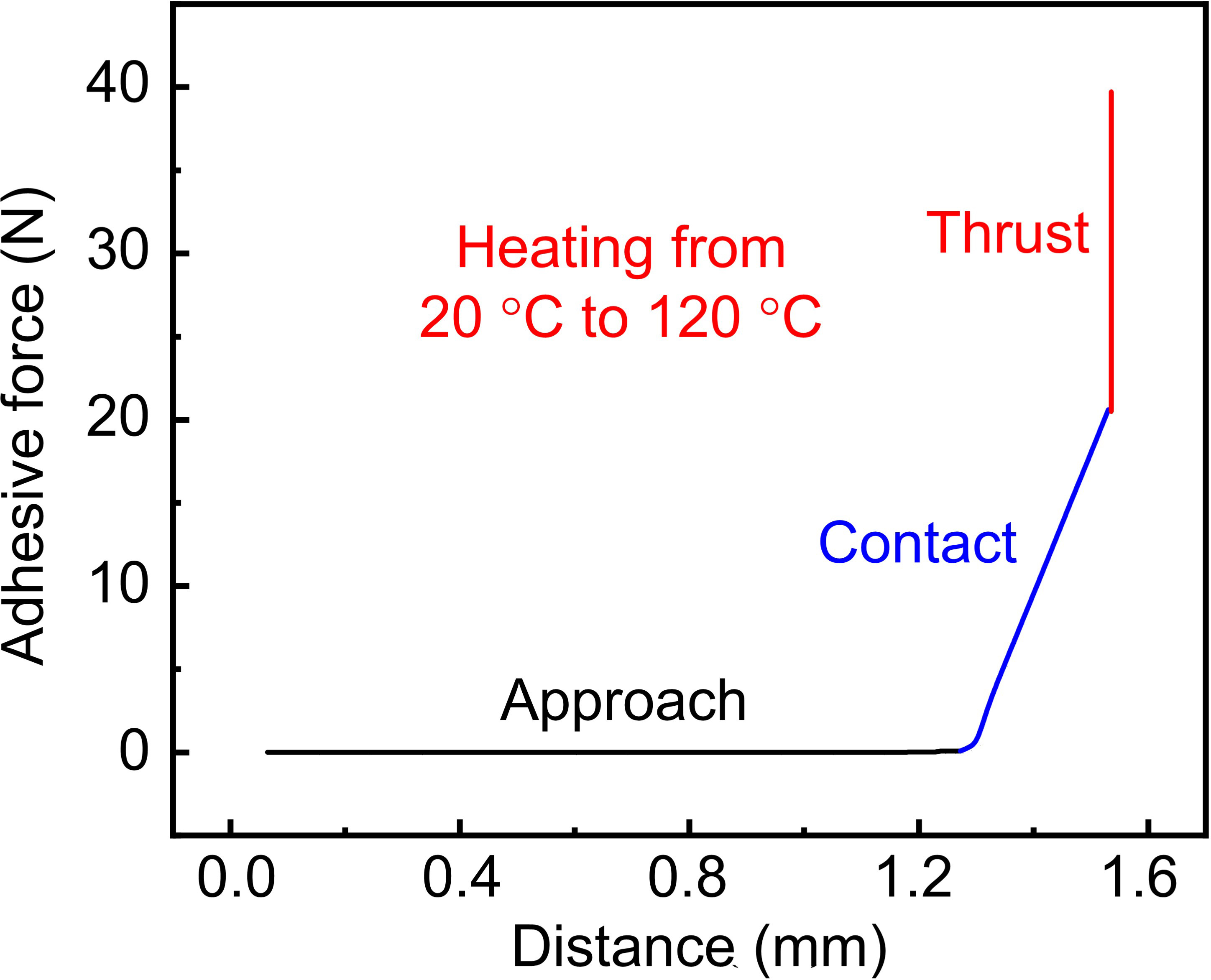


Fig. S17 Measured force-displacement curves of thrust force by a Material Testing System. To measure the thrust force, the heating of stamp from the ambient temperature (20 °C) to a higher temperature (120 °C) after the contact of chip with the stamp yields an increased load, which is the thrust force.


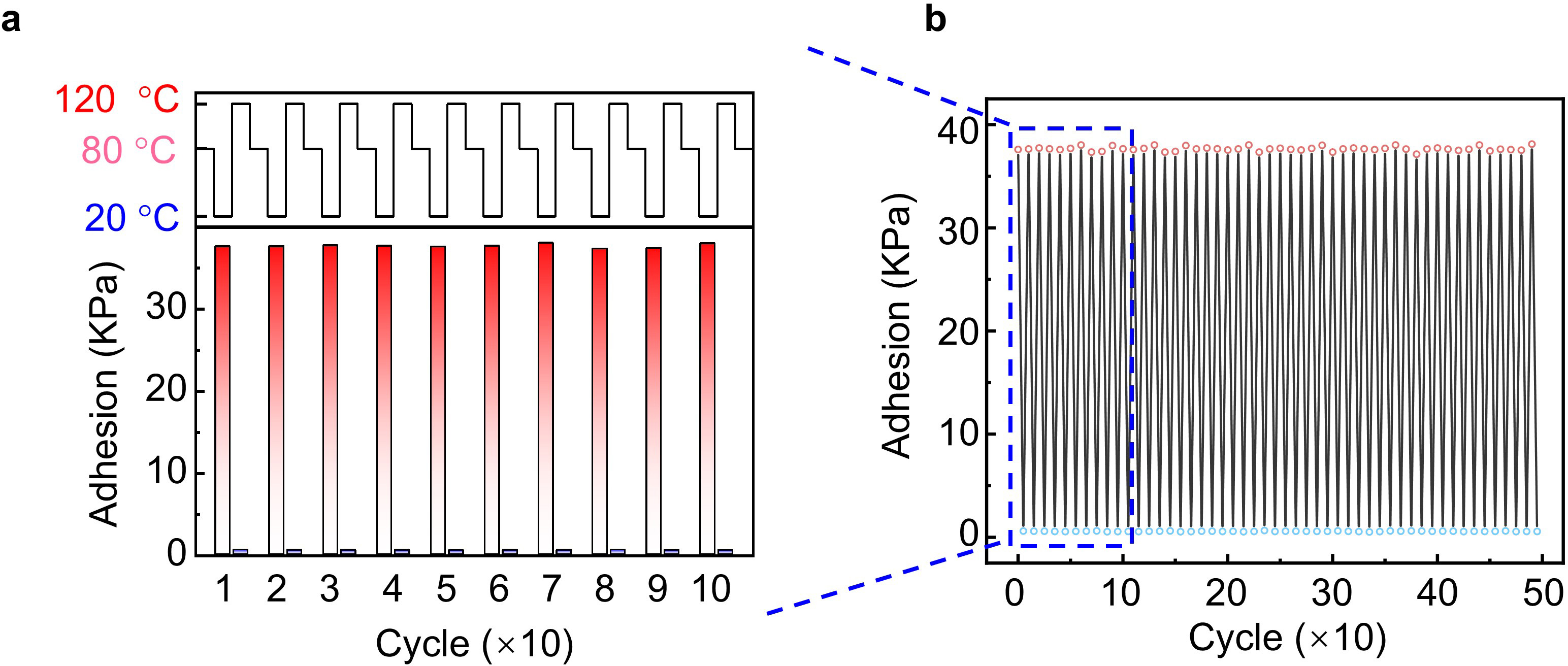


Fig. S18 Cycle tests of the TCGC-embedded adhesive stamp for adhesion strength using the Material Testing System. a 100-cycle test of the adhesive strength of the stamp during the chip pick-up and release process (i.e. heating and cooling). b 500-cycle test of the adhesive strength of the stamp during the chip pick-up and release process.


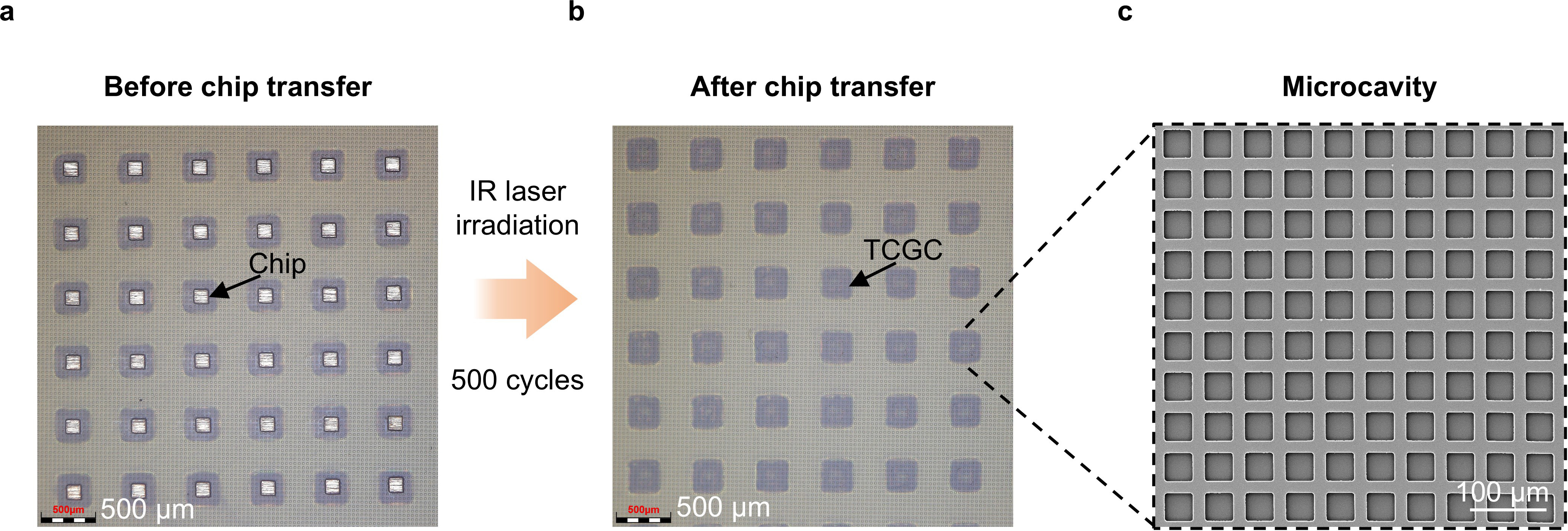


Fig. S19 Morphological observations of the TCGC-embedded stamp before and after repeated chip transfer under a 0.5-W IR laser irradiation. a Optical image of the stamp before the transfer of microchips sized of 150 µm. b Optical image of the stamp after the transfer of microchips. c SEM image of microcavity of the TCGC-embedded adhesive stamp.


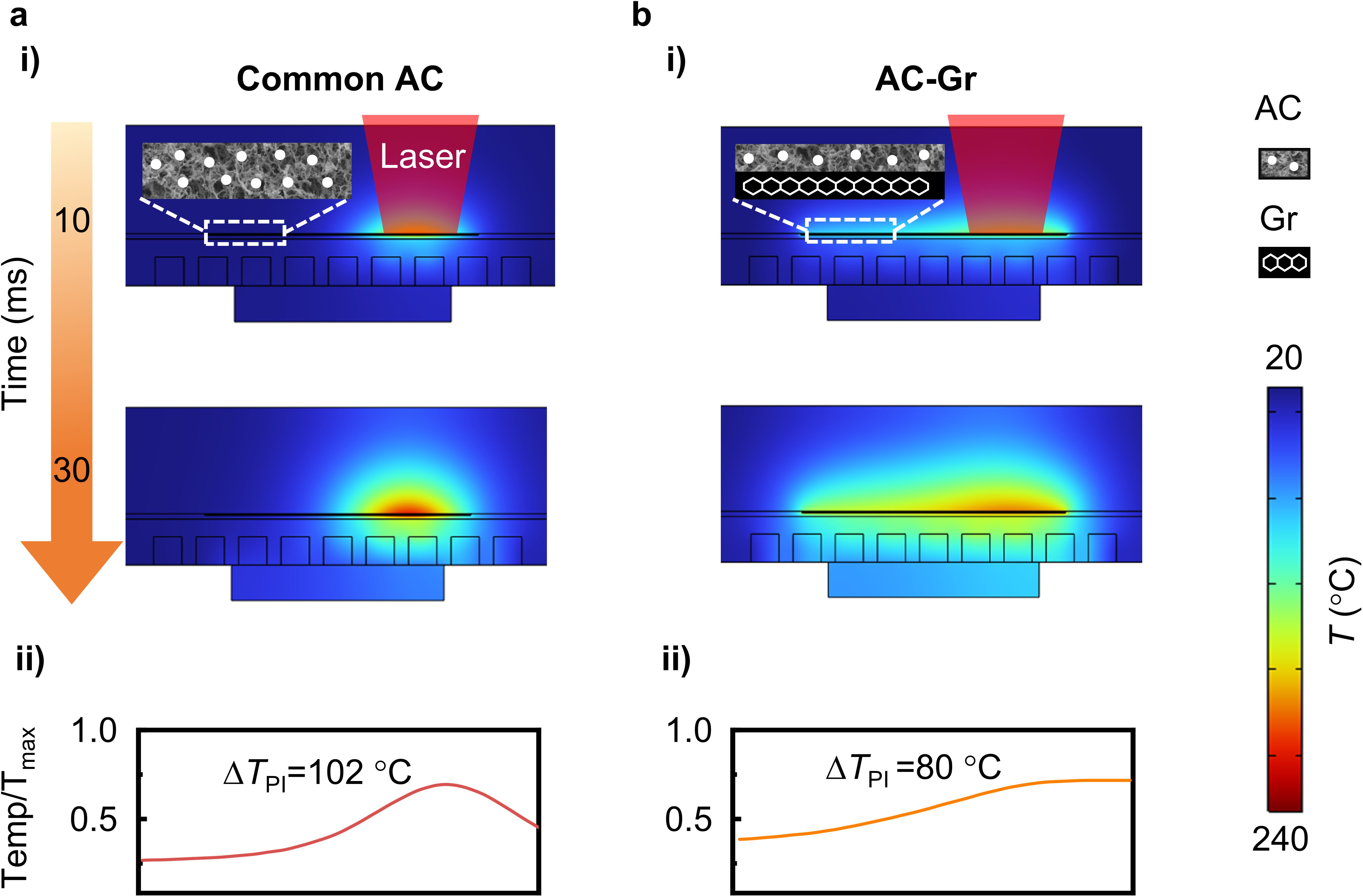


Fig. S20 Simulated temperature field of the stamps under 30-ms misaligned laser irradiation for two different photothermal conversion layers: common AC and AC-Gr. a For common AC structure: (i) Temperature distribution at 10 ms and 30 ms. (ii) Relative temperature distribution along the horizontal coordinate at the bottom of PI layer at 30 ms. b For AC-Gr structure: (i) Temperature distribution at 10 ms and 30 ms. (ii) Relative temperature distribution along the horizontal coordinate at the bottom of PI layer at 30 ms.


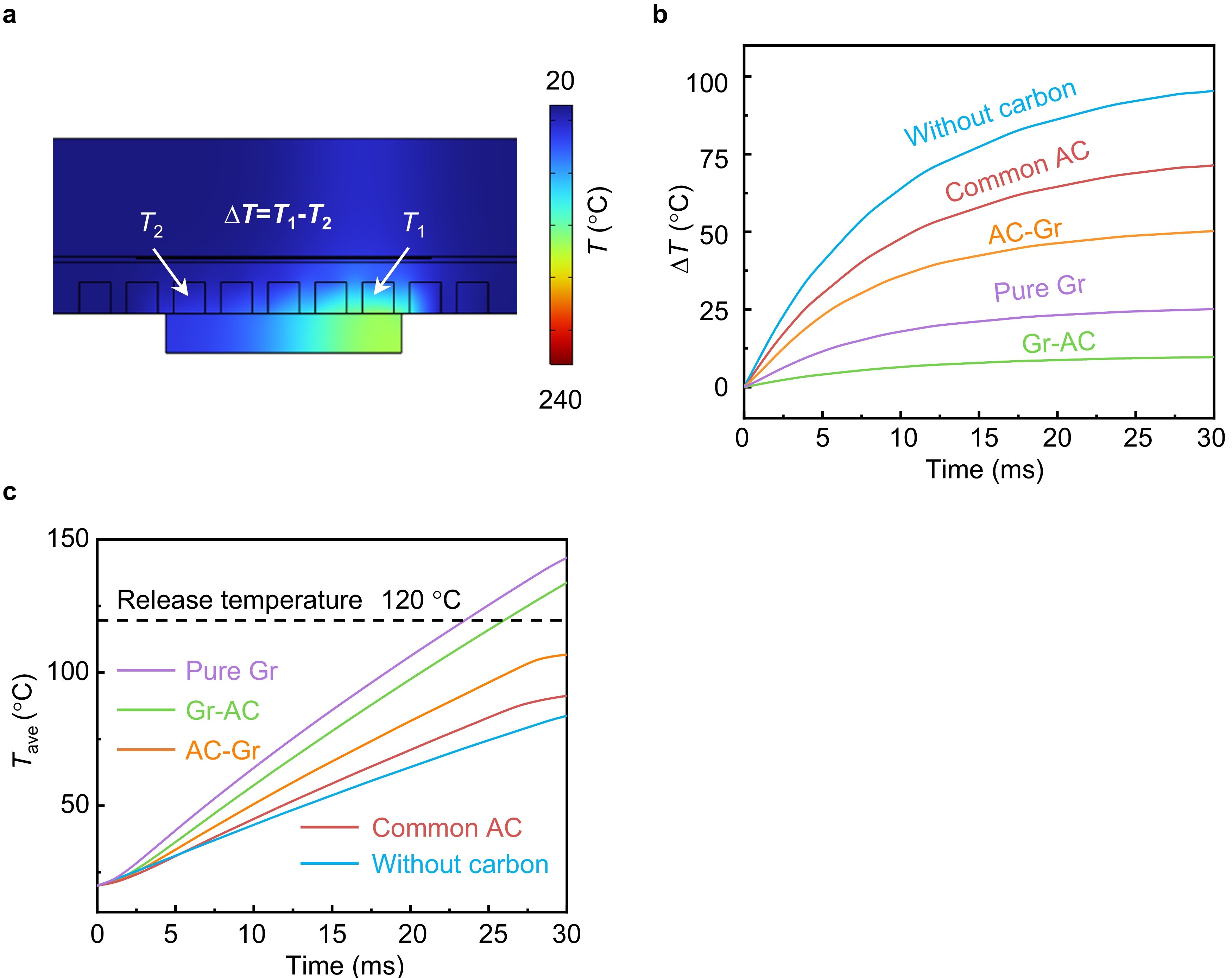


Fig. S21 Temperature distribution of the stamp with five different photothermal conversion layers: without carbon (by chip absorption), common AC, AC-Gr, pure Gr and Gr-AC. a Simulated temperature field of the stamp without carbon under the misaligned laser irradiation at 10 ms. b The temperature difference of the cavities (∆*T*) under the misaligned laser irradiation for five different photothermal conversion layers. c Average temperature of the cavities above the chip as a function of laser irradiation time.


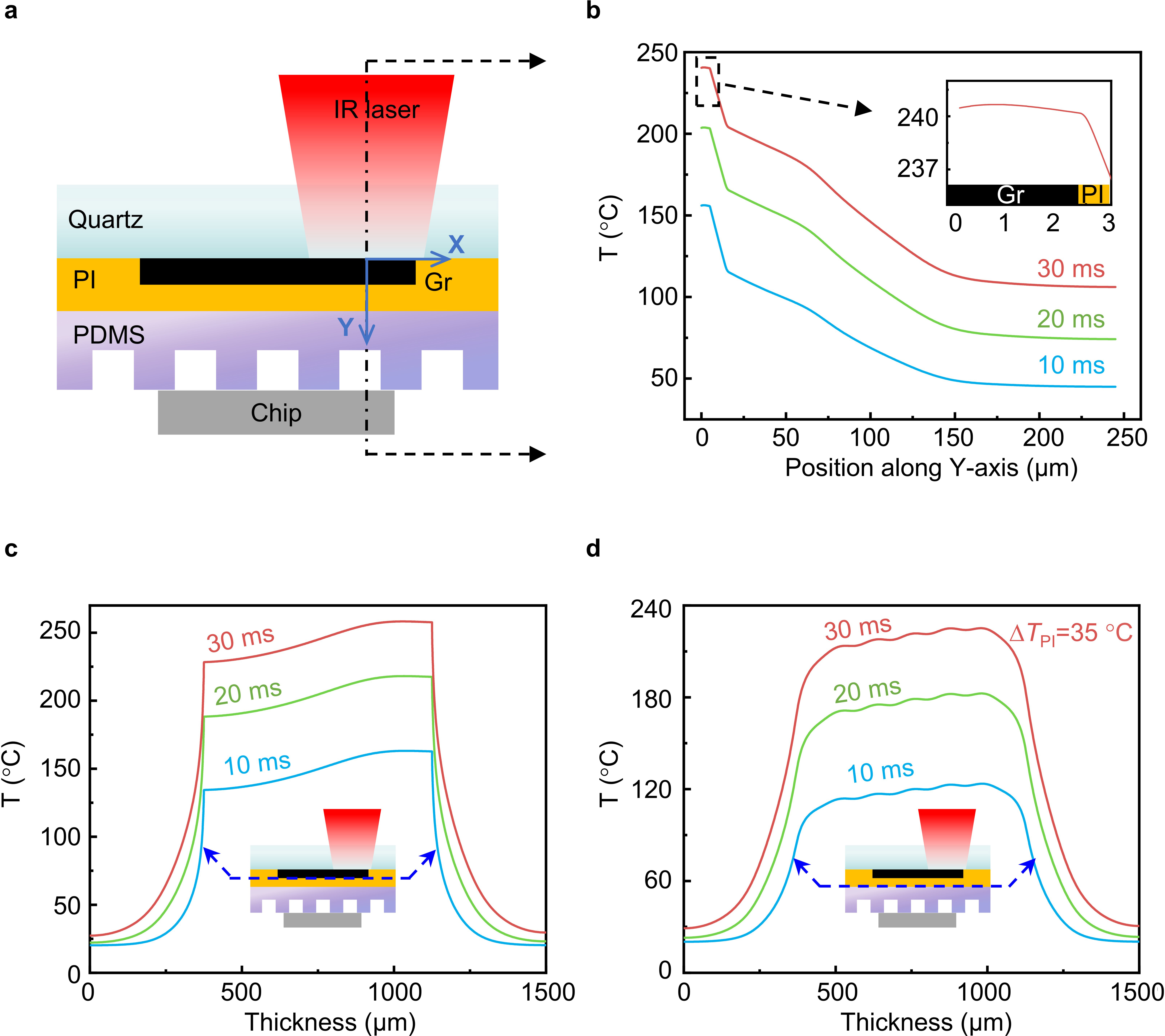


Fig. S22 Temperature distribution of a stamp with pure Gr under misaligned laser irradiation was calculated by FEA. a Schematic illustration of the stamp with pure Gr under a 30-ms misaligned laser irradiation. b Temperature distribution of the stamp with pure Gr along the Y-axis direction at 10 ms, 20 ms and 30 ms. c Temperature profile at the bottom of graphene layer inside the stamp under the misaligned laser irradiation at 10 ms, 20 ms and 30 ms. d Temperature profile at the bottom of PI layer at 10 ms, 20 ms and 30 ms (Δ*T*_PI_=35 ℃).


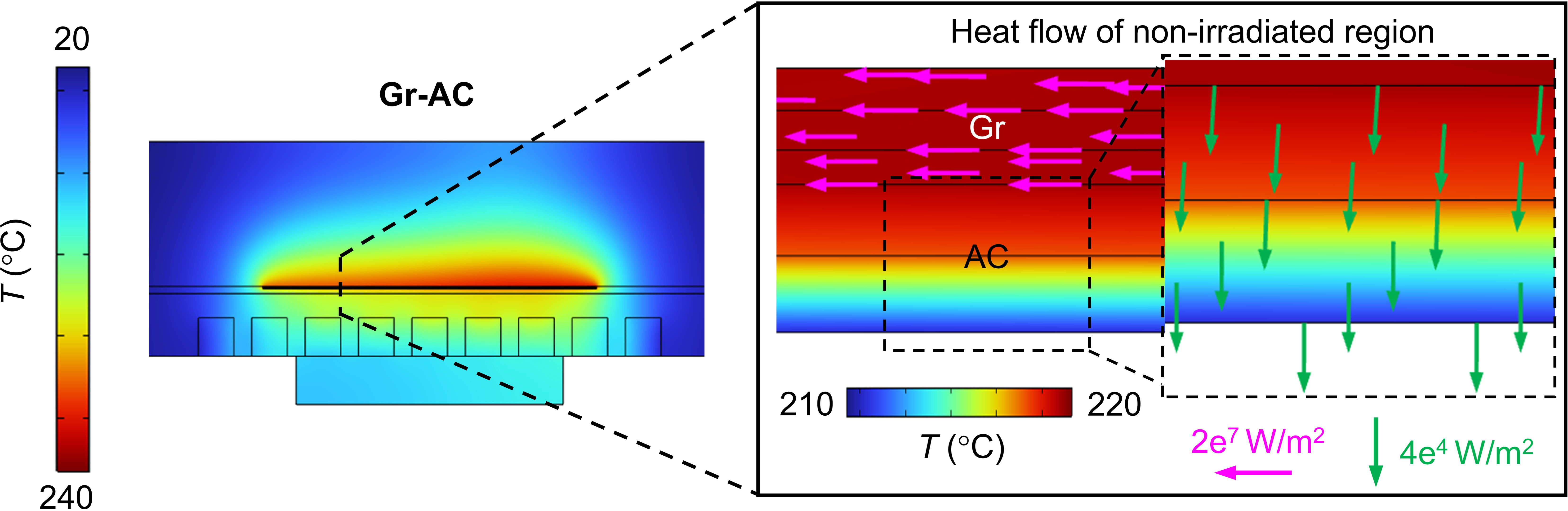


Fig. S23 Heat flow distribution in the laser non-irradiated region inside the stamp with Gr-AC structure at 30 ms. The upper graphene layer rapidly conducts heat laterally (~10^7^ W·m^-2^) to the non-irradiated region and transfers it downward to the lower AC layer (~10^4^ W·m^-2^), thereby ensuring a uniform temperature distribution at the bottom of the PI layer.


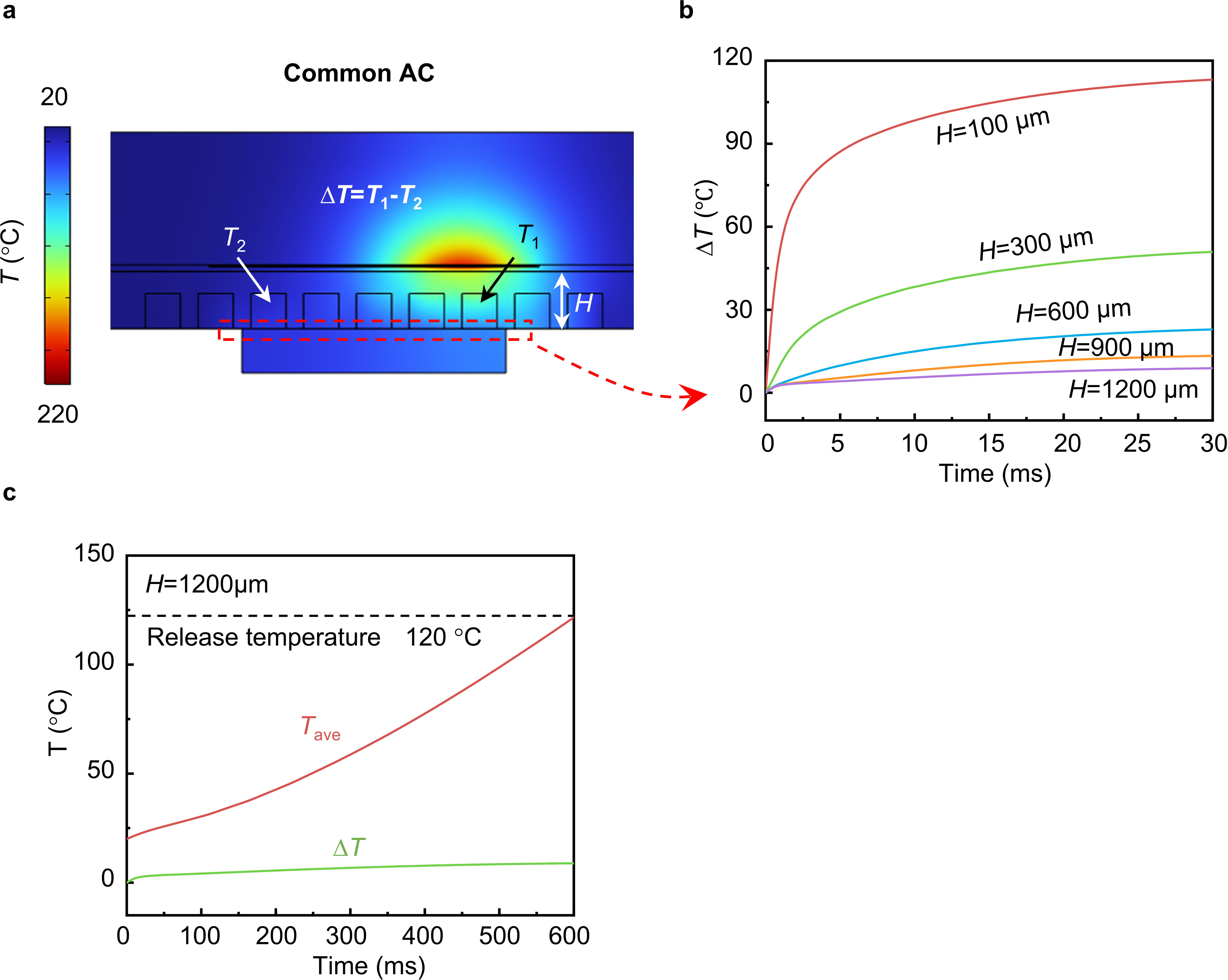


Fig. S24 Effect of heat homogenization for the stamp with common AC as the thickness of the PDMS layer is increased. a Simulated temperature field of the stamp with common AC during the misaligned laser irradiation at 30 ms. b Temperature difference of the cavities (∆*T*) for different thicknesses (100 µm ~1200 µm) of the PDMS layer under 30-ms misaligned IR laser irradiation. c Average temperature of the cavities (*T*_ave_) and temperature difference (∆*T*) of the stamp with the PDMS layer of thickness of 1200 µm under 600-ms misaligned IR laser irradiation.


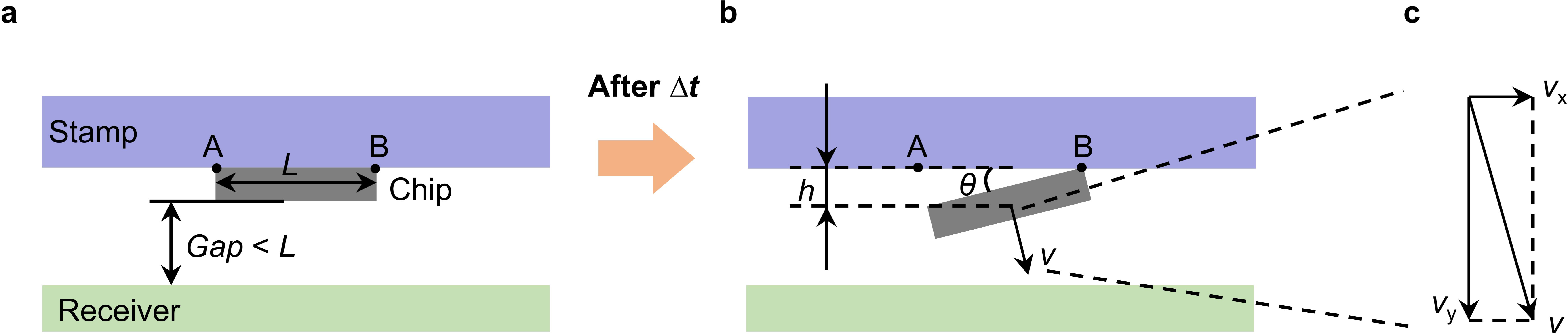


Fig. S25 Asynchronous delamination of the chip from the stamp creates a horizontal velocity that reduces transfer accuracy. a Schematic diagram of the stamp attached with the chip at the initial moment. b After Δ*t* of misaligned laser irradiation, the chip will rotate around point B on the stamp, leading to the generation of a velocity *v* of the chip. c Orthogonal decomposition of the velocity at moment Δ*t*.


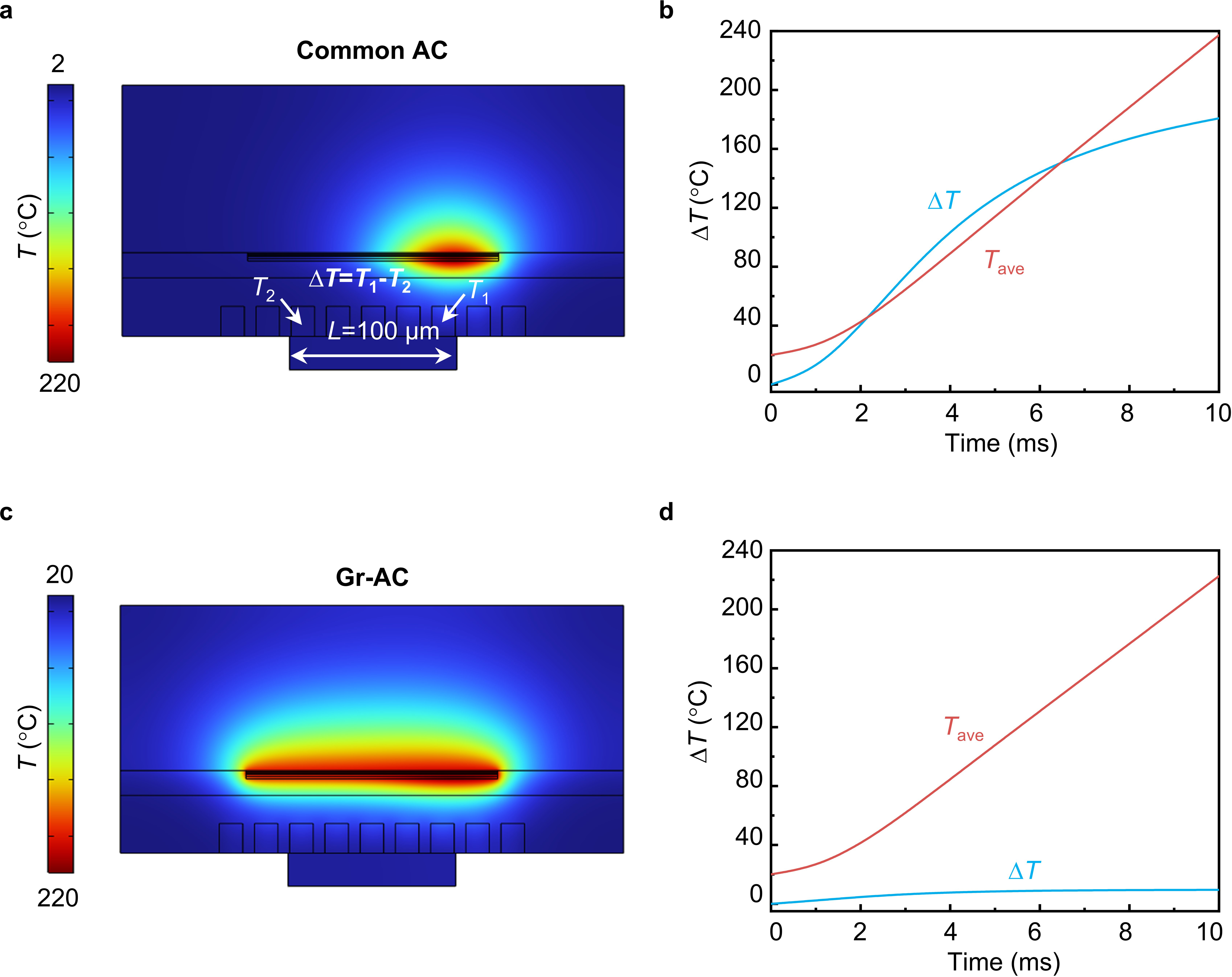


Fig. S26 Comparison of temperature fields of stamps/small-size chips (100 µm × 100 µm × 20 µm) with common AC and Gr-AC structure under 10-ms laser offset irradiation. a Simulated temperature distribution of a stamp with common AC at 10 ms. b Average temperature of the cavities (*T*_ave_) and temperature difference (∆*T*) of the stamp with common AC during the misaligned laser irradiation. c Simulated temperature distribution of a stamp with Gr-AC at 10 ms. d Average temperature of the cavities (*T*_ave_) and temperature difference (∆*T*) of the stamp with Gr-AC structure during the misaligned laser irradiation.


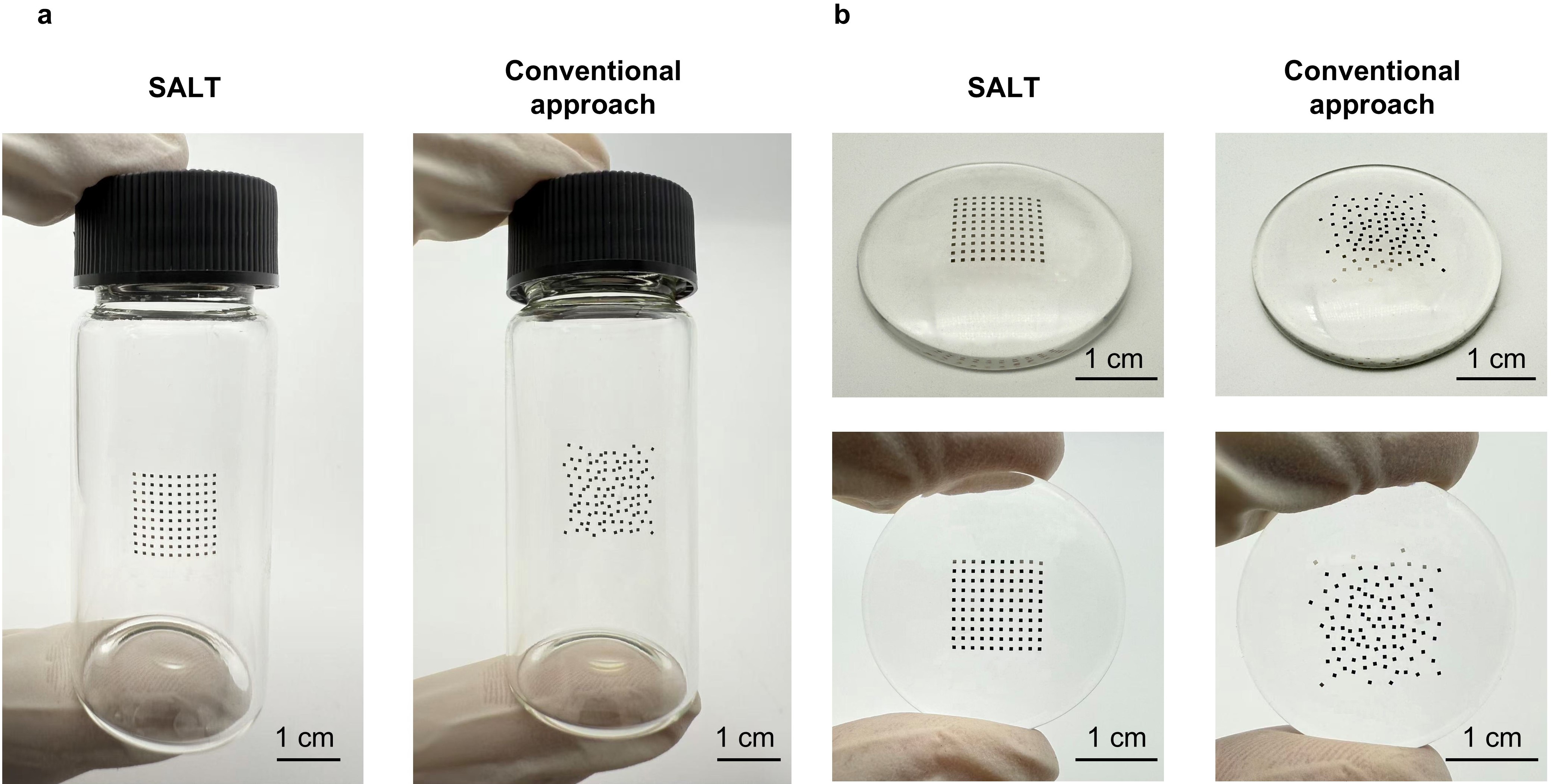


Fig. S27 Comparison of conventional approach (without TCGC) and SALT for transferring titanium chips (400 µm × 400 µm × 30 µm) onto low-curvature surfaces using planar stamps under IR laser offset irradiation. a Optical images of microchips printed onto cylindrical surfaces (3 cm in diameter). b Optical images of microchips printed onto spherical surfaces (5 cm in diameter).


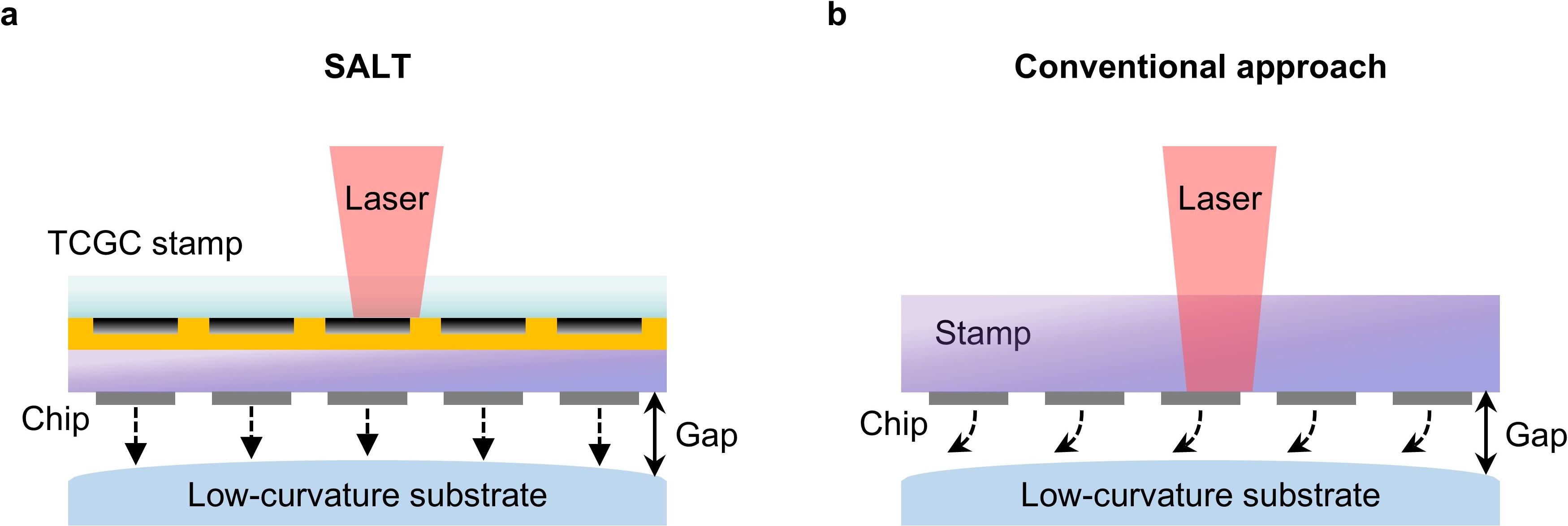


Fig. S28 Comparison of SALT and conventional approach (without TCGC) for transferring titanium chips onto low-curvature surfaces using planar stamps under IR laser offset irradiation. a Schematic illustration of transferring microchips onto low-curvature surfaces via SALT. b Schematic illustration of transferring microchips onto low-curvature surfaces via conventional approach.


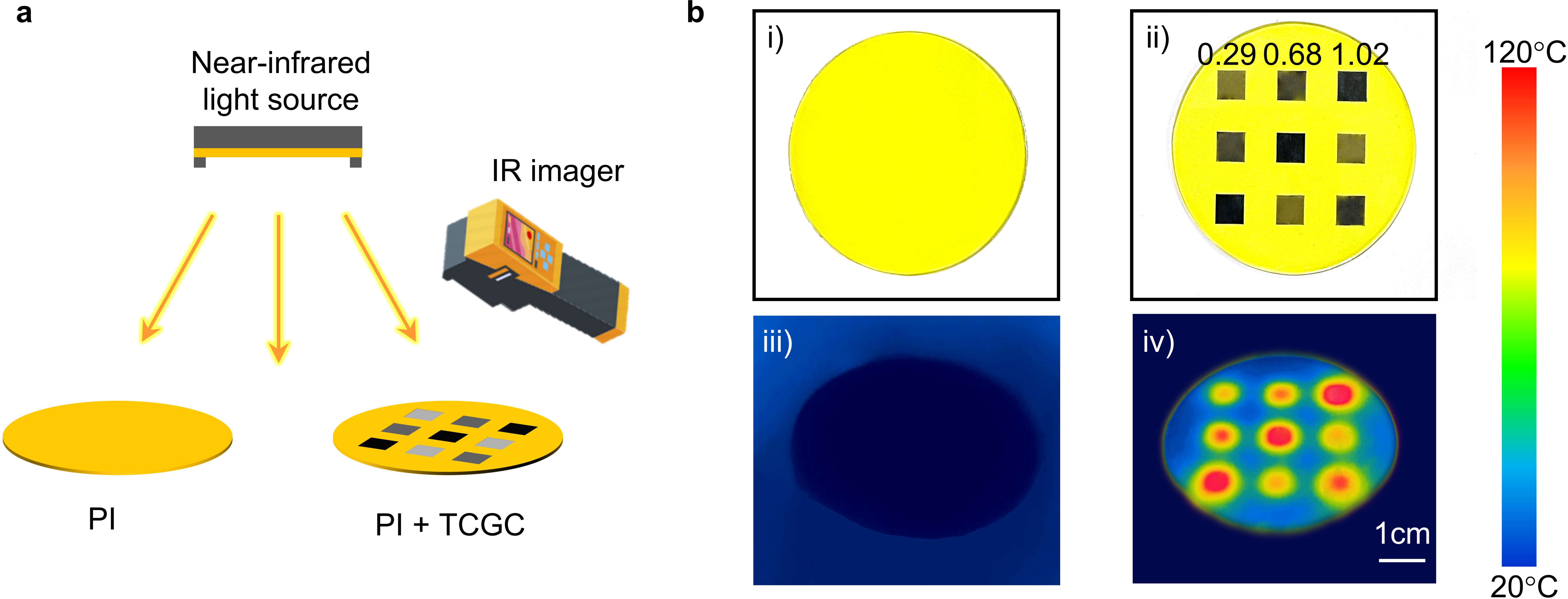


Fig. S29 Thermal camera observations of three distinct TCGC layers under IR lamp irradiation. a Schematic illustration of thermal camera observation for pure PI and PI with three distinct TCGC layers irradiated by an infrared lamp. b Optical images and infrared thermography of three distinct TCGC layers (absorbance of 0.29, 0.68, 1.02) and pure PI under IR lamp irradiation.


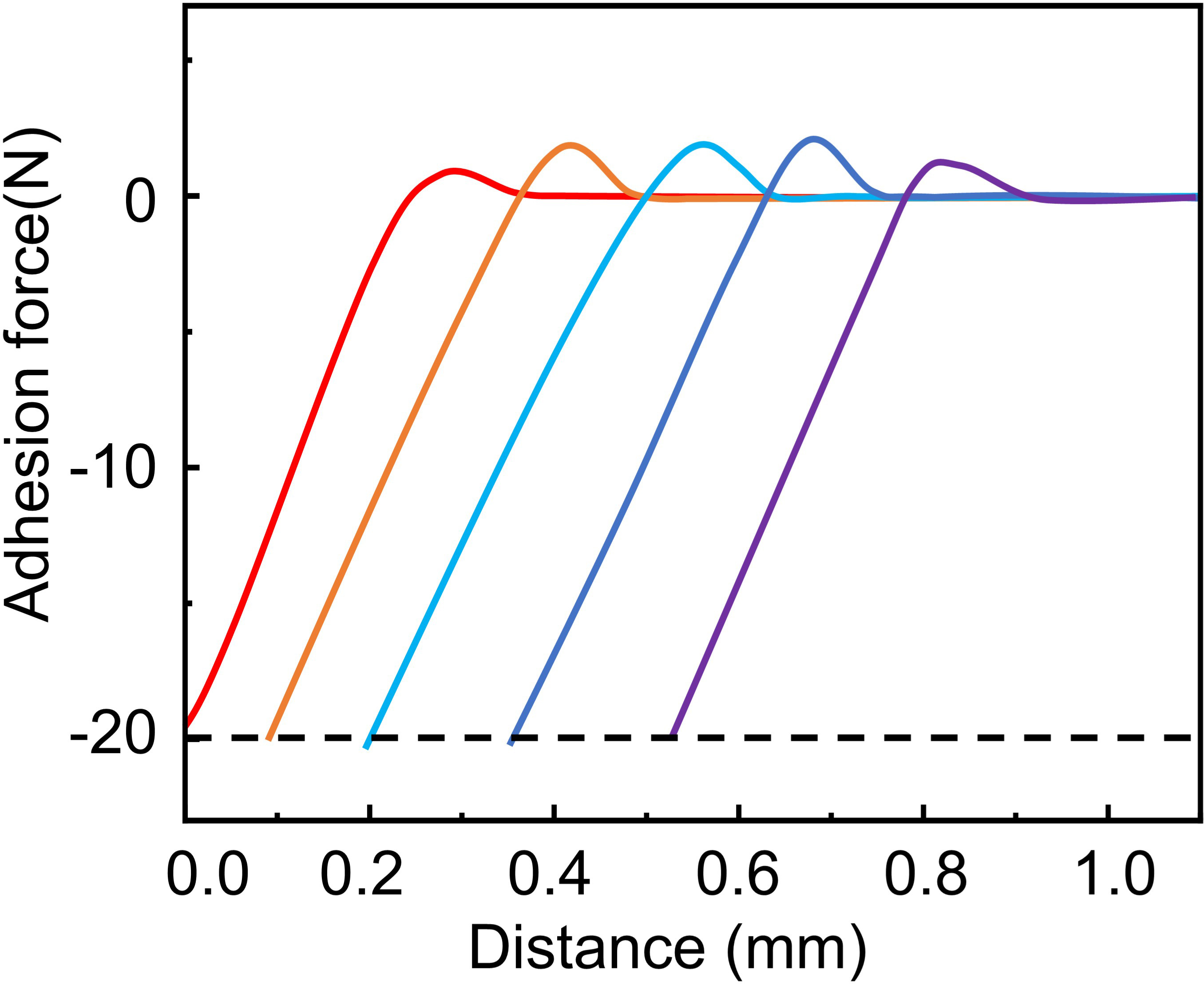


Fig. S30 Interfacial adhesion forces between the PDMS stamp and the silicon chip are tested using the Material Testing System.


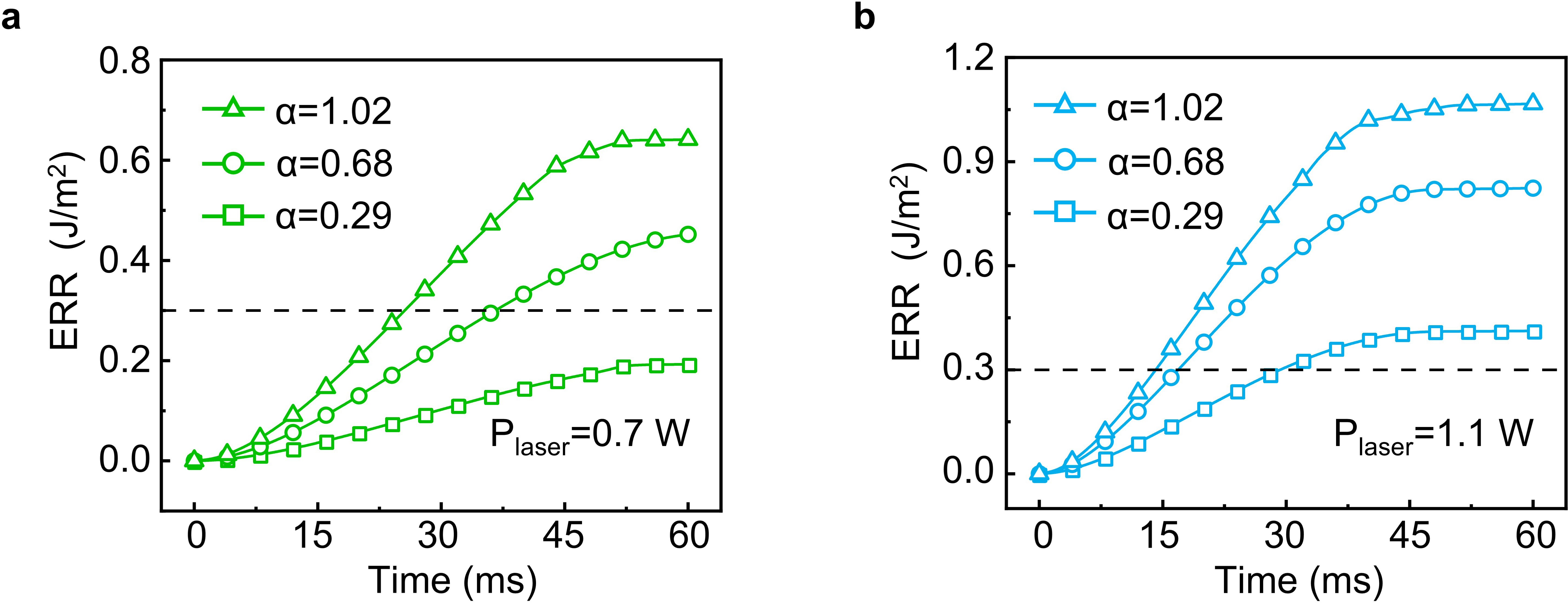


Fig. S31 The crack tip energy release rates (ERR) at the chip and stamp interface as a function of laser durations were calculated by FEA under different conditions: a at the laser power of 0.7 W and b at the laser power of 1.1 W.


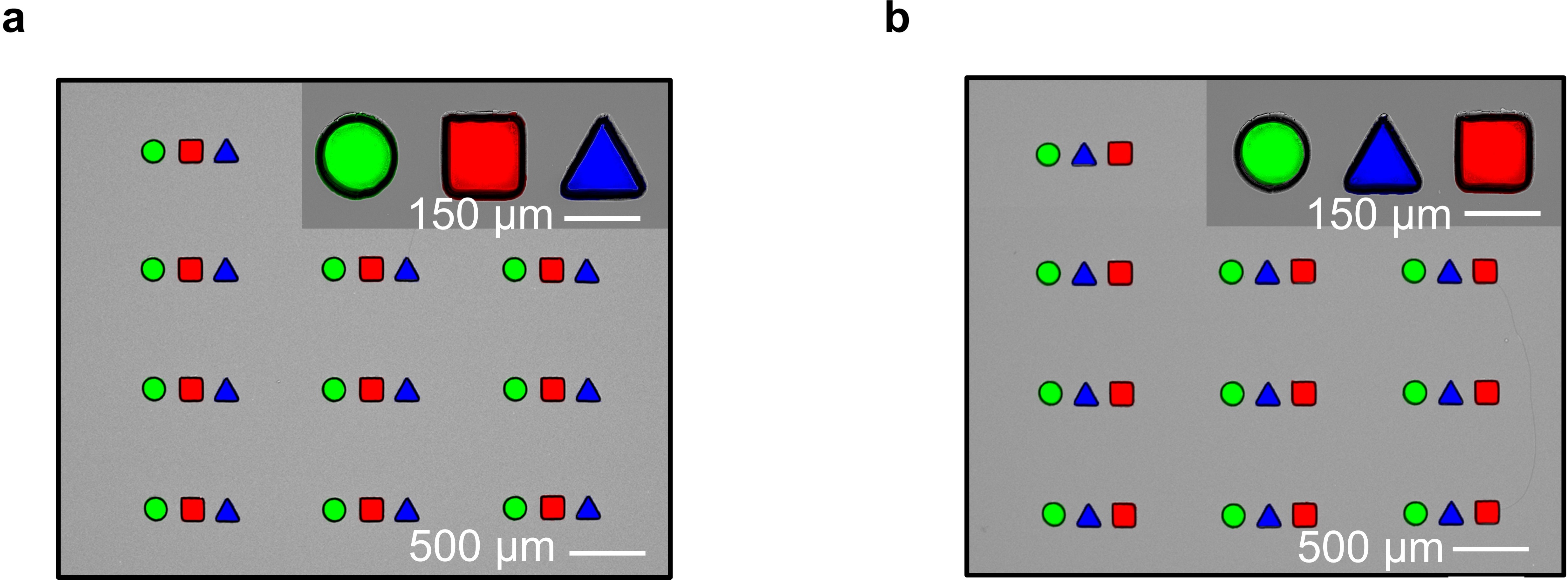


Fig. S32 Demonstrations of multiple transfer printing of different shapes (square, circular, and triangular) of microchips via SALT.


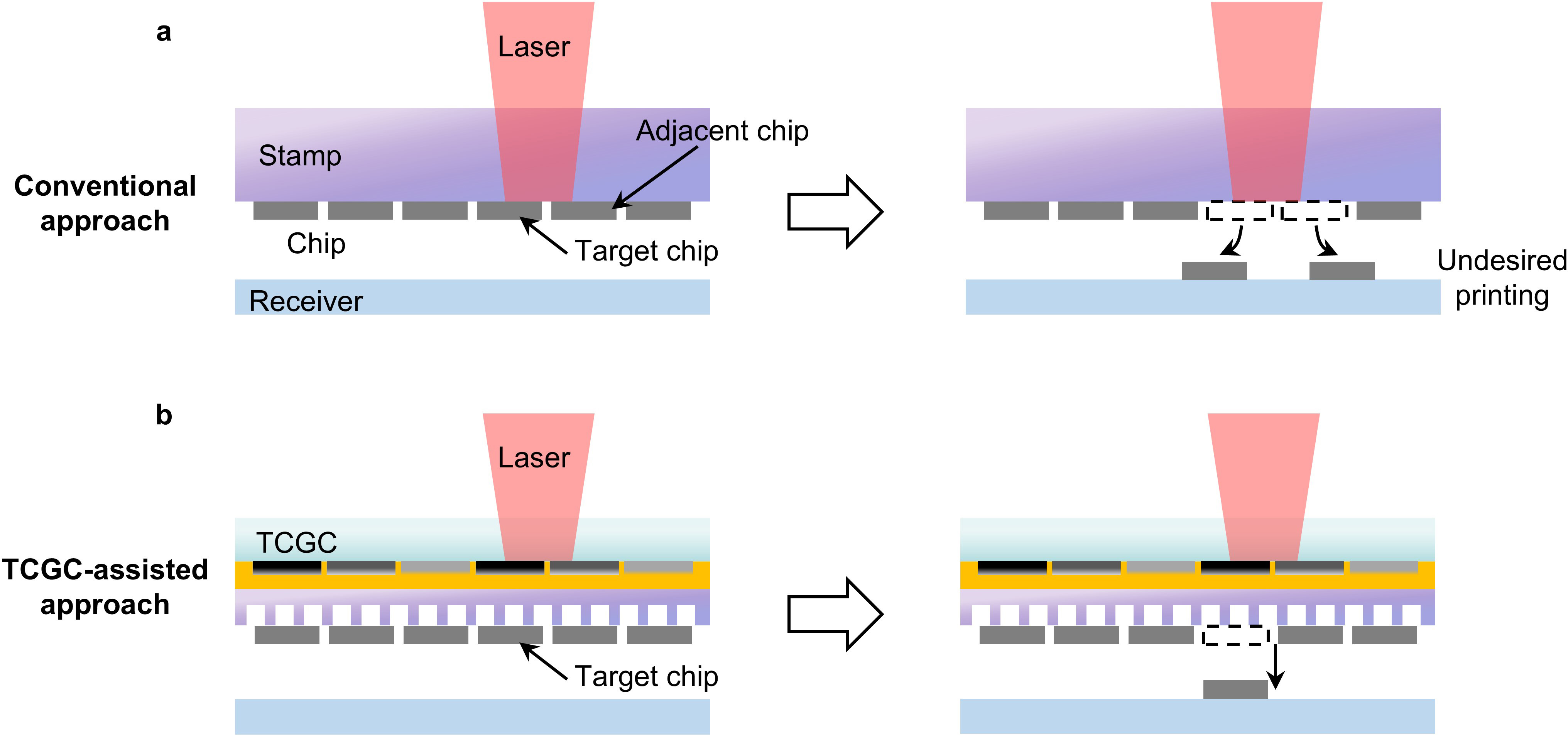


Fig. S33 Comparison of conventional approach (pre-planned scanning paths) and SALT in the transfer of densely arranged chips (e.g., MicroLED chips) under laser offset irradiation. a Schematic illustration of undesired printing of chips in conventional approach. b Schematic illustration of no mis-transfer even if mis-irradiation hits the adjacent chip in SALT.


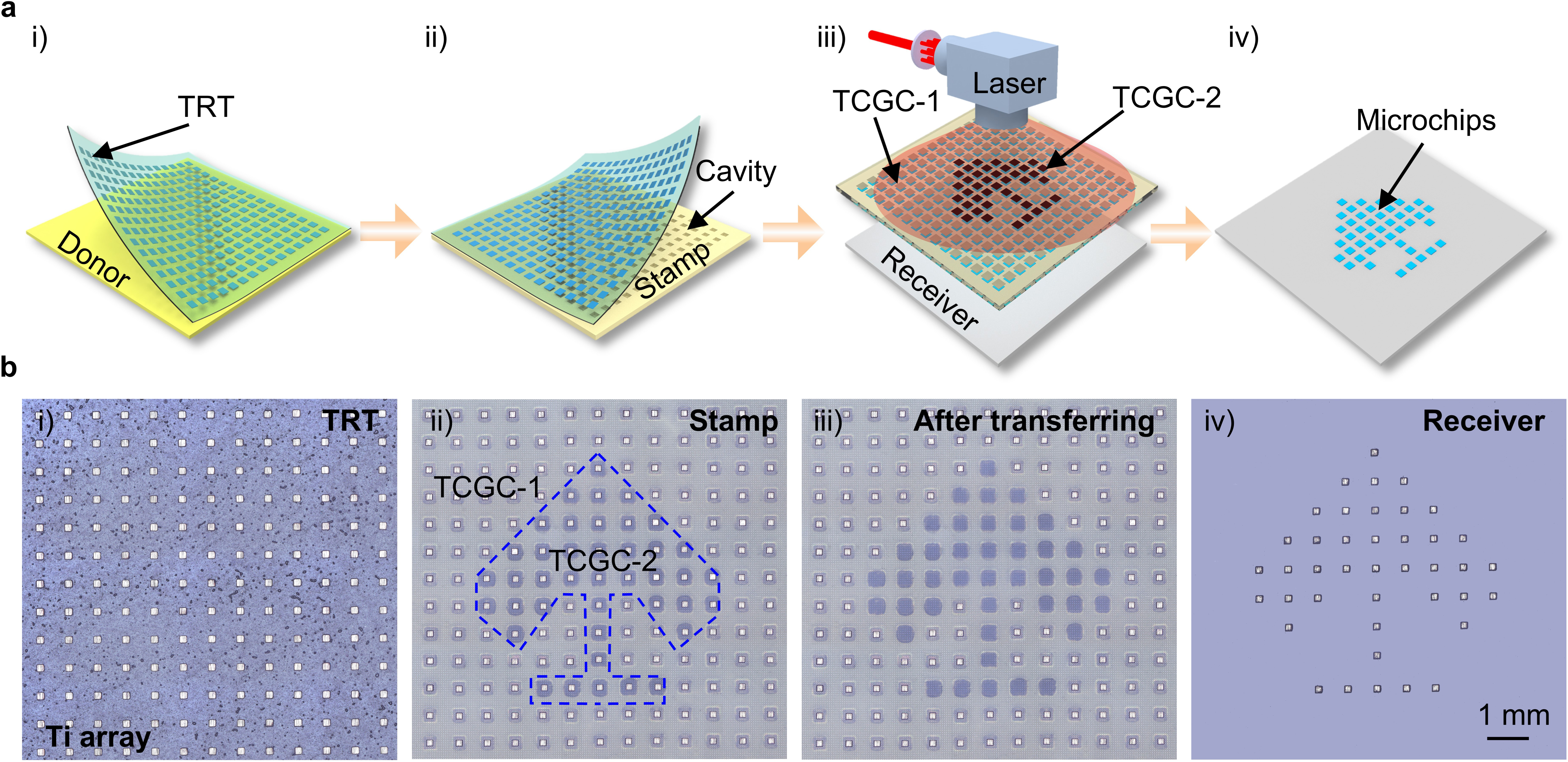


Fig. S34 Programmable transfer printing process of microchips by SALT. (*A*) Schematic illustrations of transfer printing process. a (i) A thermal release tape (TRT) picks up microchips from a donor. (ii) A stamp picks up the microchips from the heated TRT. (iii) The microchips are selectively transfer-printed onto a receiver by overall IR laser scanning. (iv) A predefined pattern of microchips (“spade” in card games) is printed on the receiver. b Optical images of a SALT process. (i) The microchip array is adhered by the TRT. (ii) The TCGC-embedded adhesive stamp picks up the microchips from the TRT. (iii) The remaining microchips on the stamp after transferring. (iv) The printed "spade" pattern on a PDMS receiver.


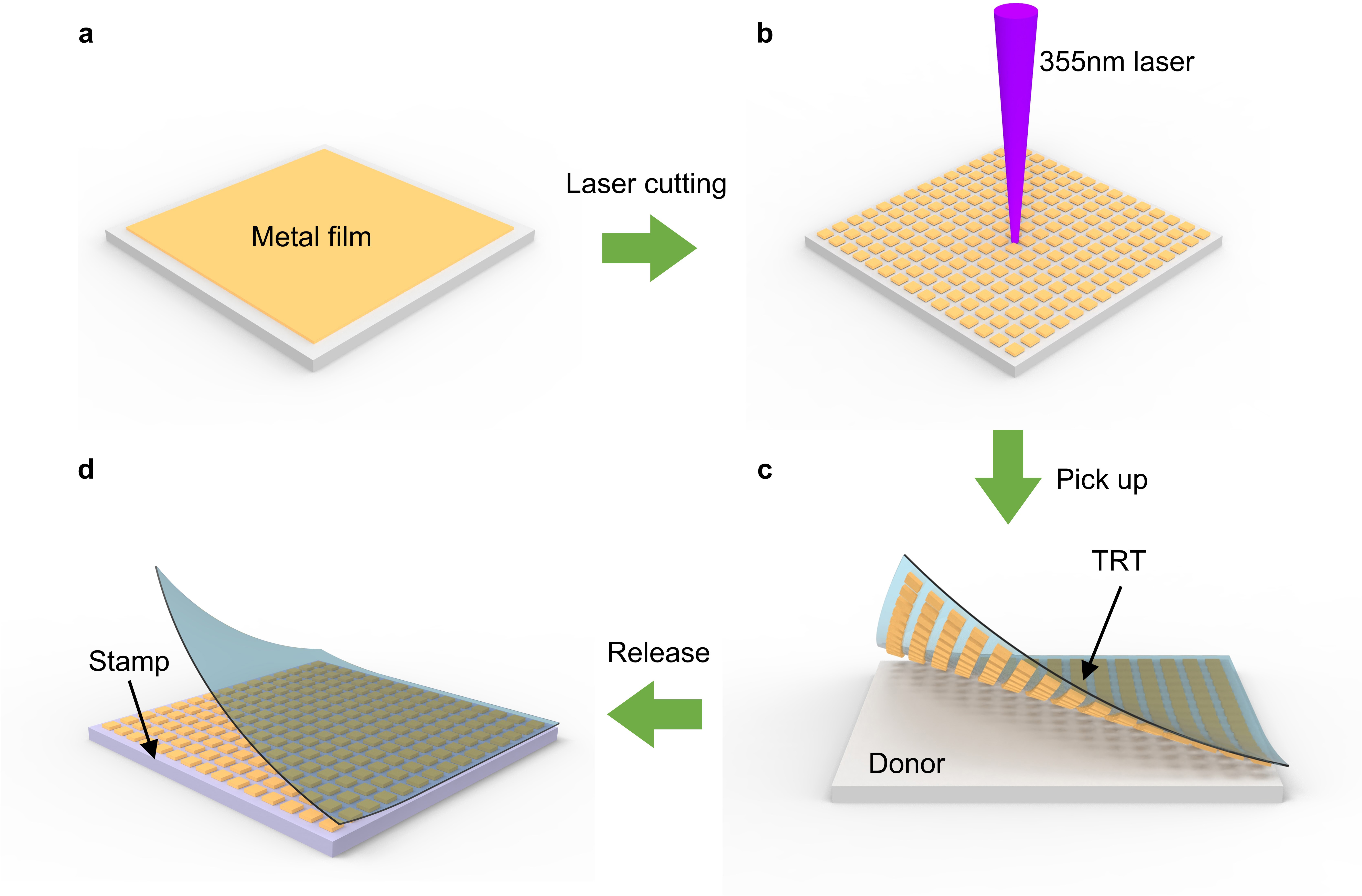


Fig. S35 Fabrication process of the microchips. a A metal film is attached to a glass substrate uniformly. b The metal film is cut by a UV laser cutter to fabricate a microchip array. c A thermal release tape (TRT) picks up the microchips from the glass. d The stamp picks up the microchips from the heated TRT.


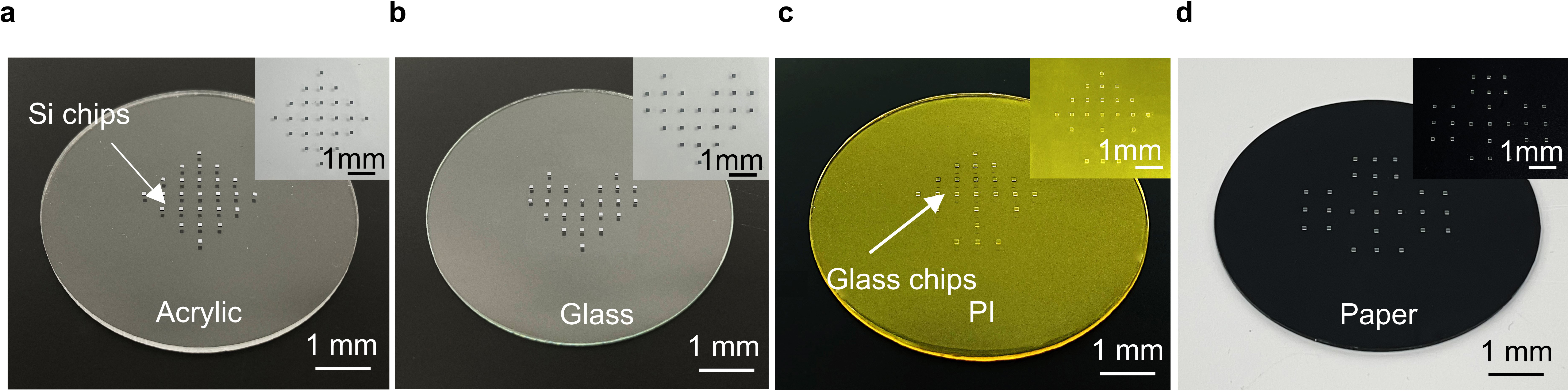


Fig. S36 Demonstrations of different chips (silicon and glass chips) transfer-printed onto challenging non-adhesive surfaces by SALT. a Acrylic board. b Glass substrate. c PI substrate. d Paper substrate.


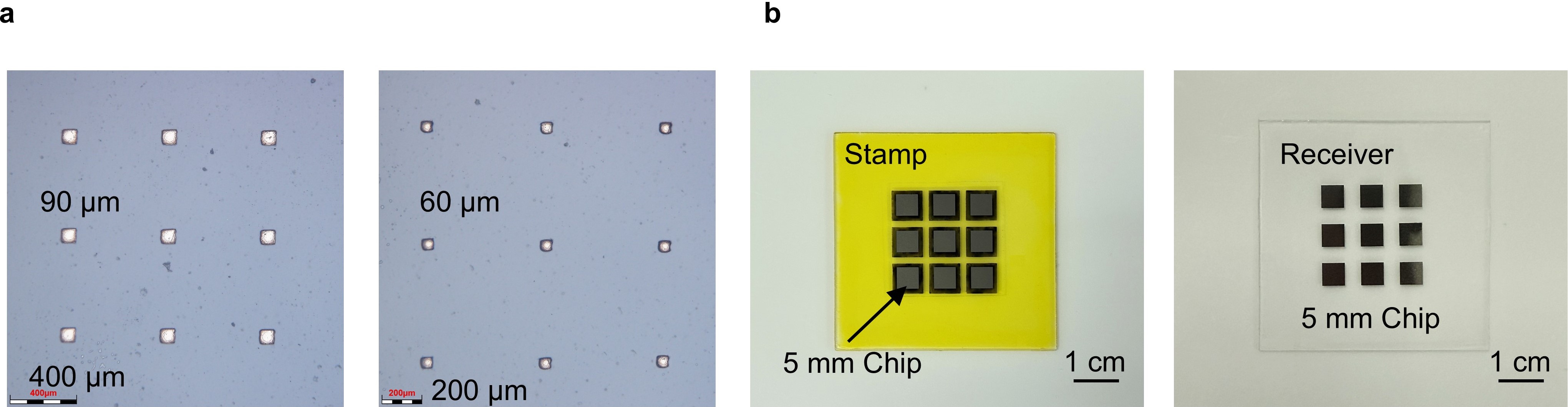


Fig. S37 The investigation of the size limit of the transferred object. a Microchips were transferred by SALT. a Optical images of micro-chips transferred by SALT. For very small chips with a size of only ~60 µm, they can still be transferred. b Optical images of large chips (5 mm × 5 mm) transferred using a stamp embedded a 3×3 array TCGC (6 mm × 6 mm) layer.


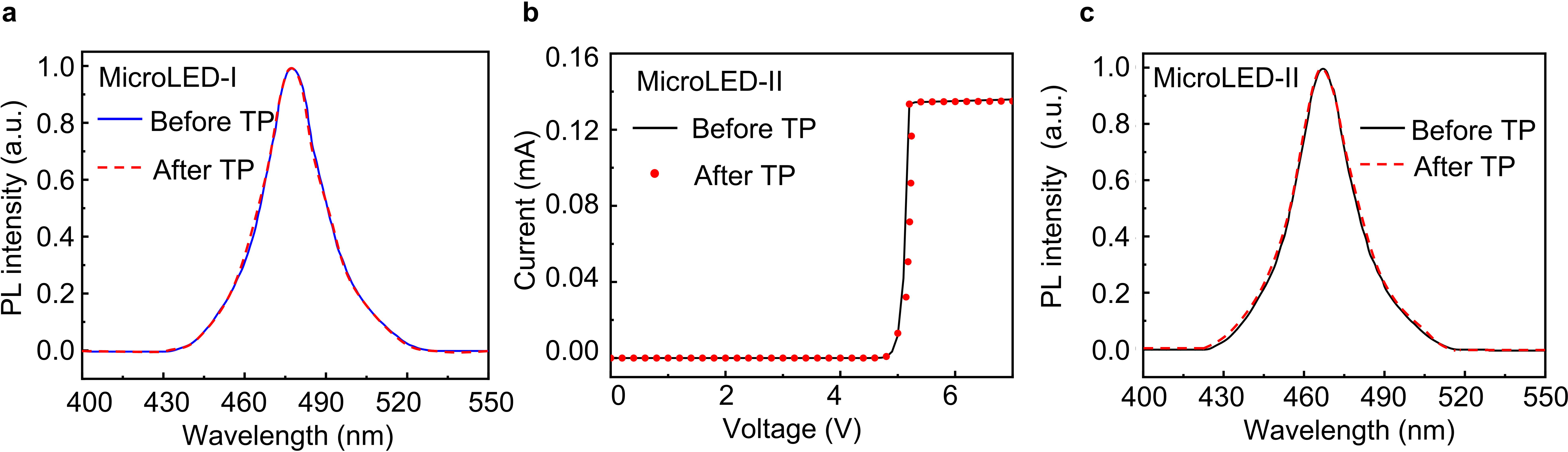


Fig. S38 Electrical and optical characteristics of the MicroLEDs before and after SALT. a Optical characteristics of the MicroLED-I before and after SALT. Electrical b and optical characteristics c of the MicroLED-II before and after SALT.


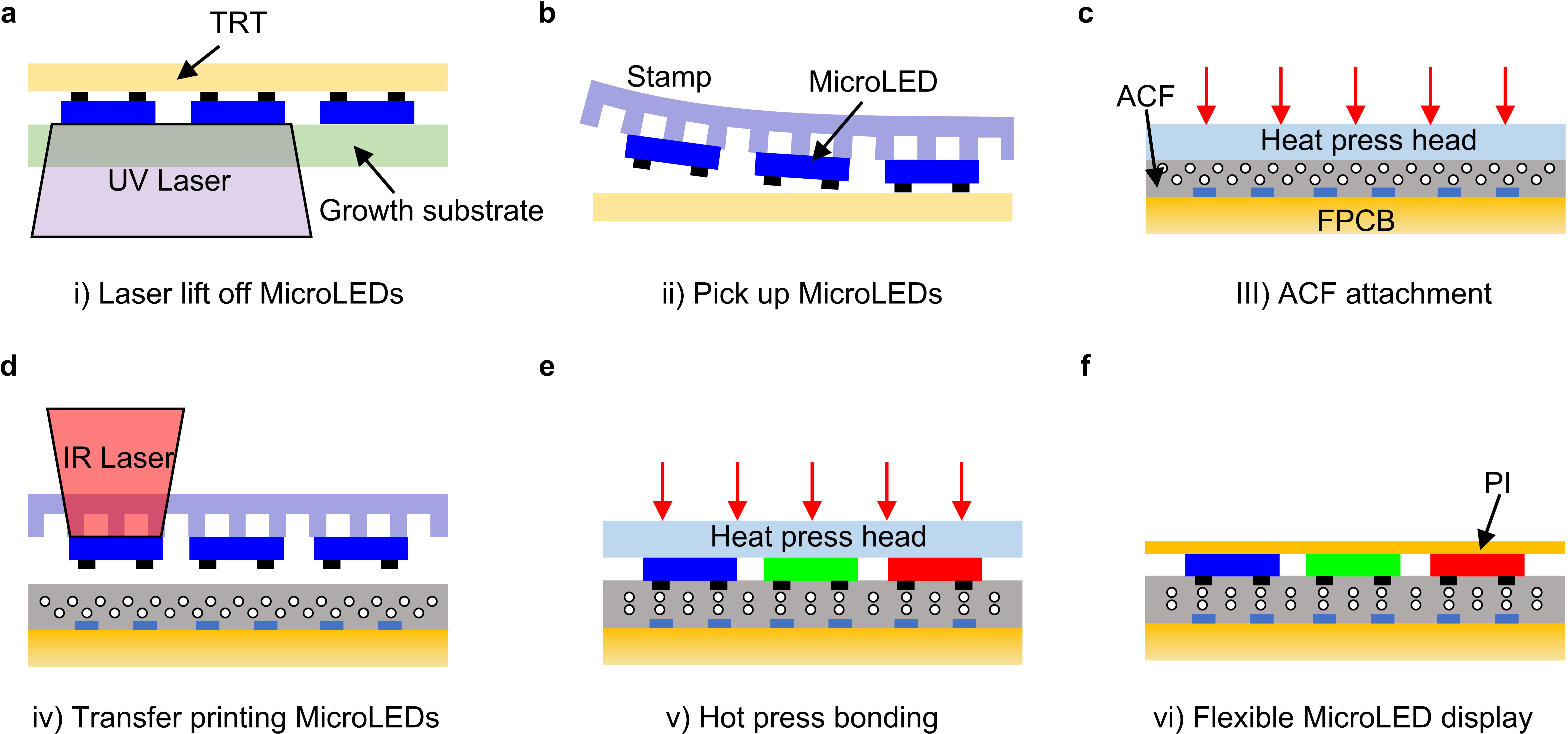


Fig. S39 Fabrication process of the flexible MicroLED display. a MicroLED chips are peeled to a thermal release tape (TRT) from the growth substrate by a UV laser. b A TCGC-embedded adhesive stamp picked up the MicroLEDs from the heated TRT. c Anisotropic conductive films (ACF) are adhered to a flexible printed circuit board (FPCB), followed by heating and pressing. d MicroLEDs are transfer-printed to the FPCB by an IR laser scanning. e The chips and FPCB are subjected to the thermo-compressive bonding. f The device is encapsulated by a PI layer.


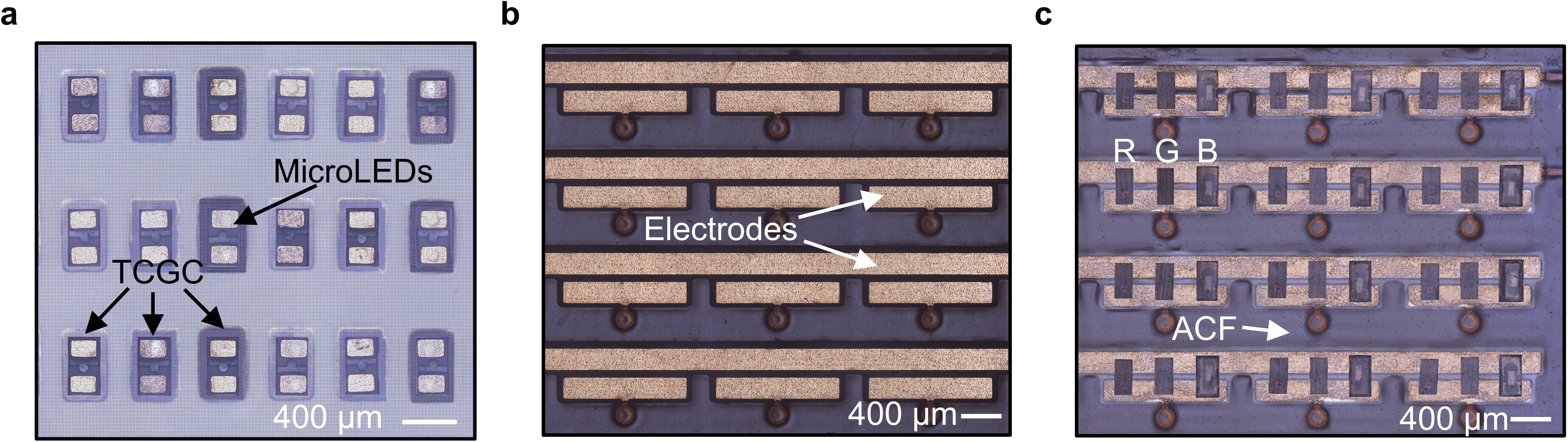


Fig. S40 Optical images of the transfer process of MicroLEDs printed on the FPCB for the flexible display. a MicroLEDs are picked up by a TCGC-embedded adhesive stamp. b Optical images of the FPCB. c The RGB MicroLEDs are printed on the circuit substrate with ACF.


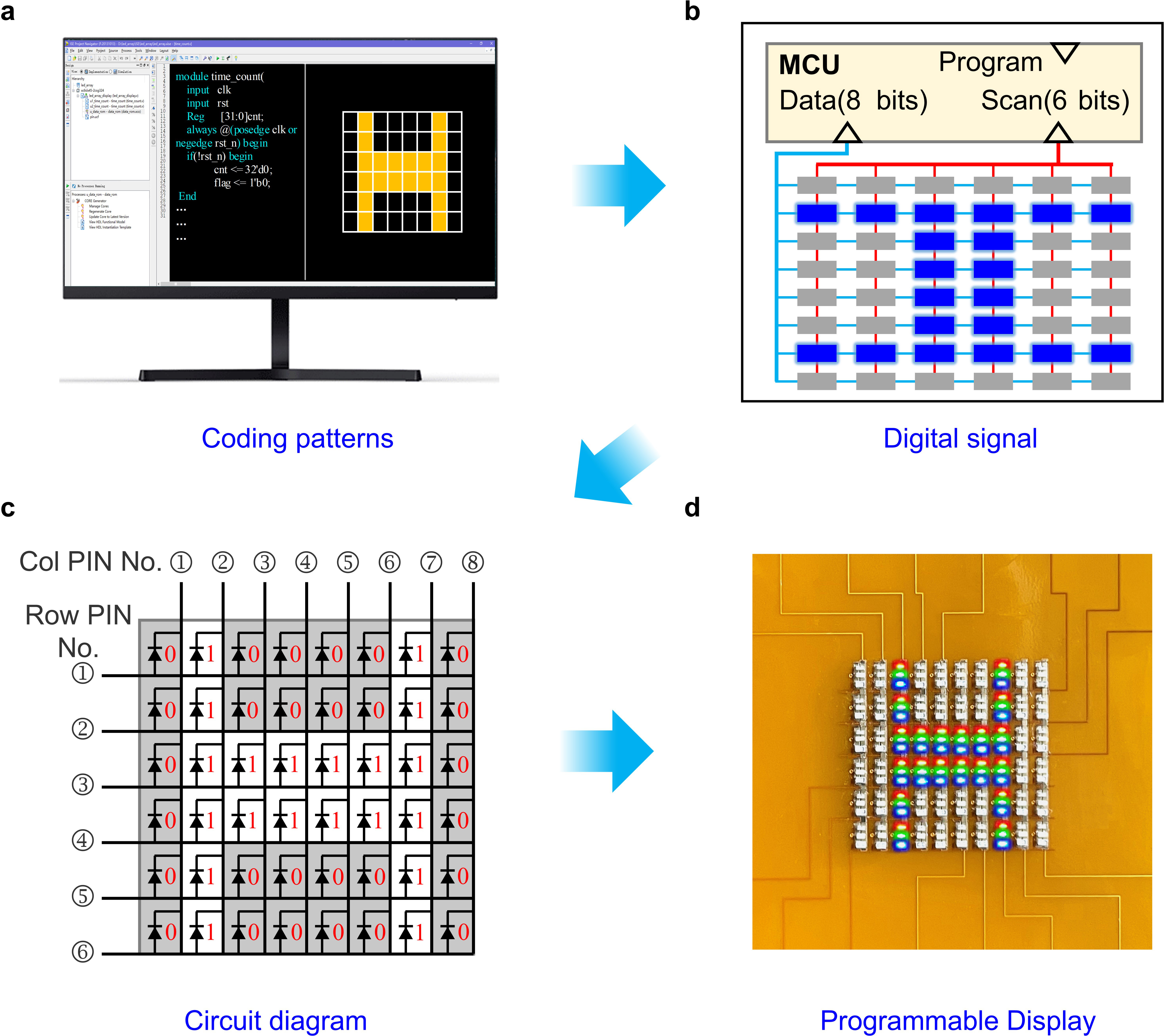


Fig. S41 A programmable MicroLED display is realized by the FPGA hardware. a Create coding patterns on a computer. b The Microcontroller outputs digital signals. c The programmable circuit diagram. d The flexible MicroLED display shows the letter of “H”.


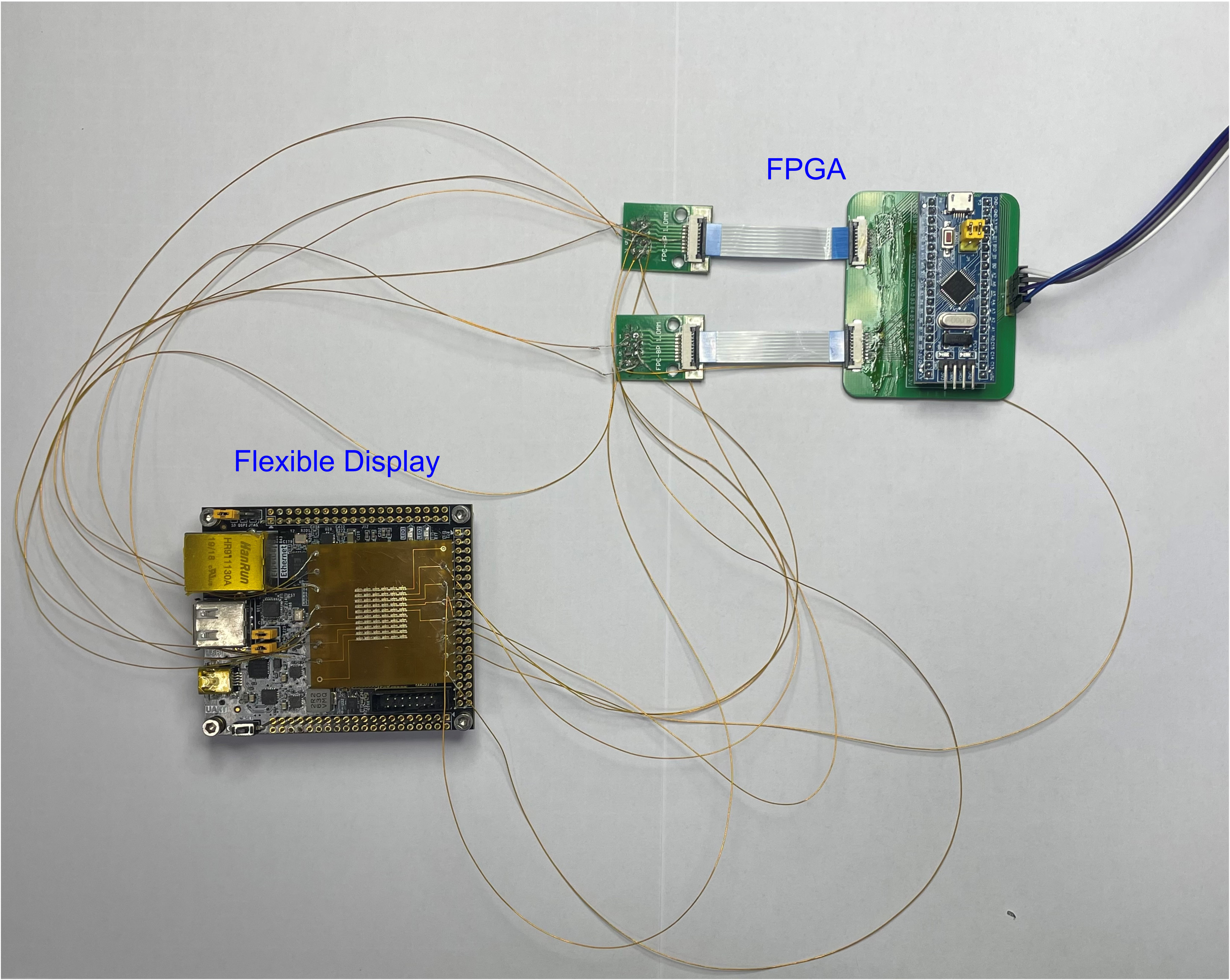


Fig. S42 Optical images of the flexible display with a programmable driver circuit.


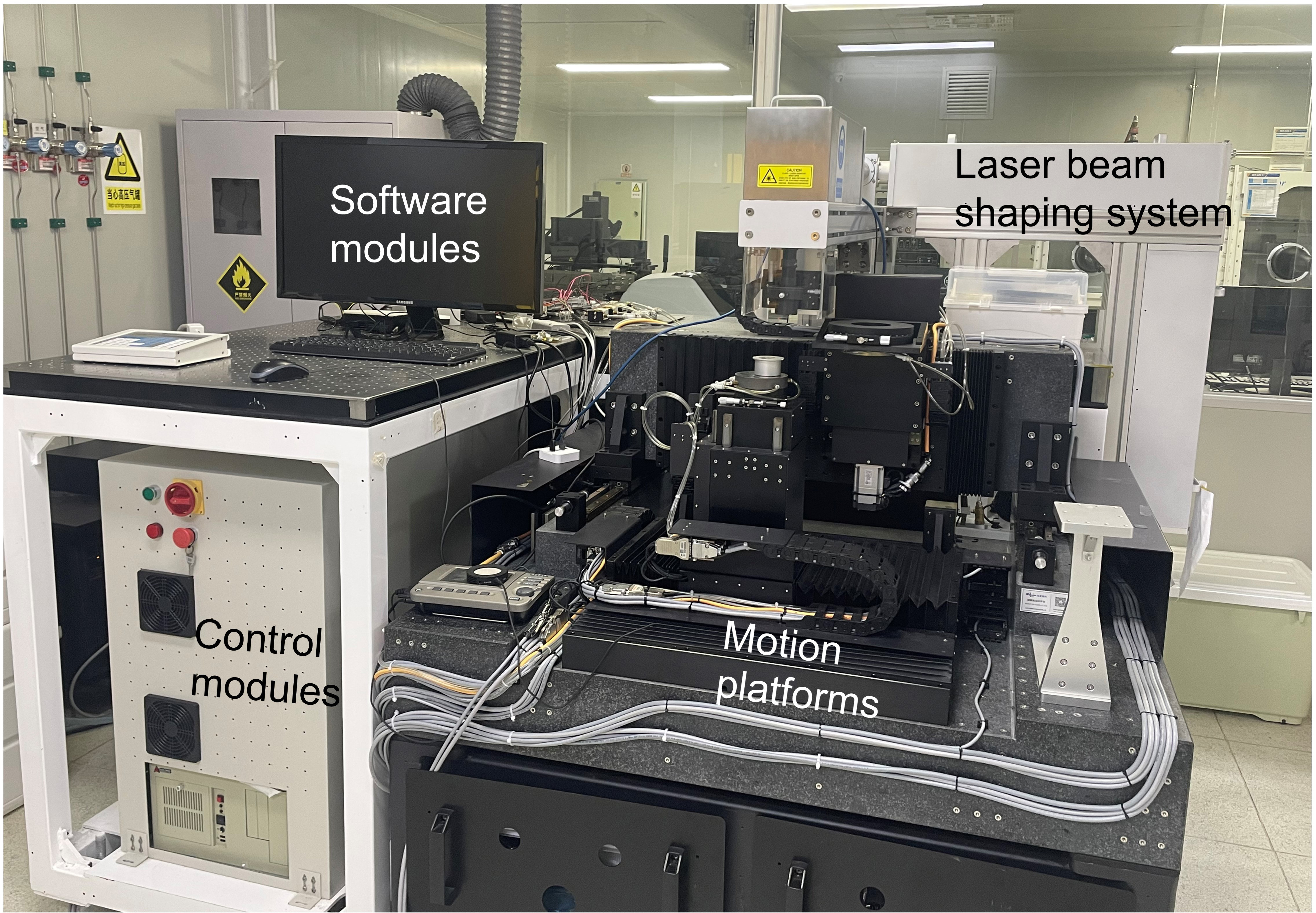


Fig. S43 Optical image of a laser transfer printing platform (iGreatTransfer). The experiment equipment consists of an excimer laser (Coherent Inc. Compex205), a laser shaping system, a software module, a control module and an alignment system.


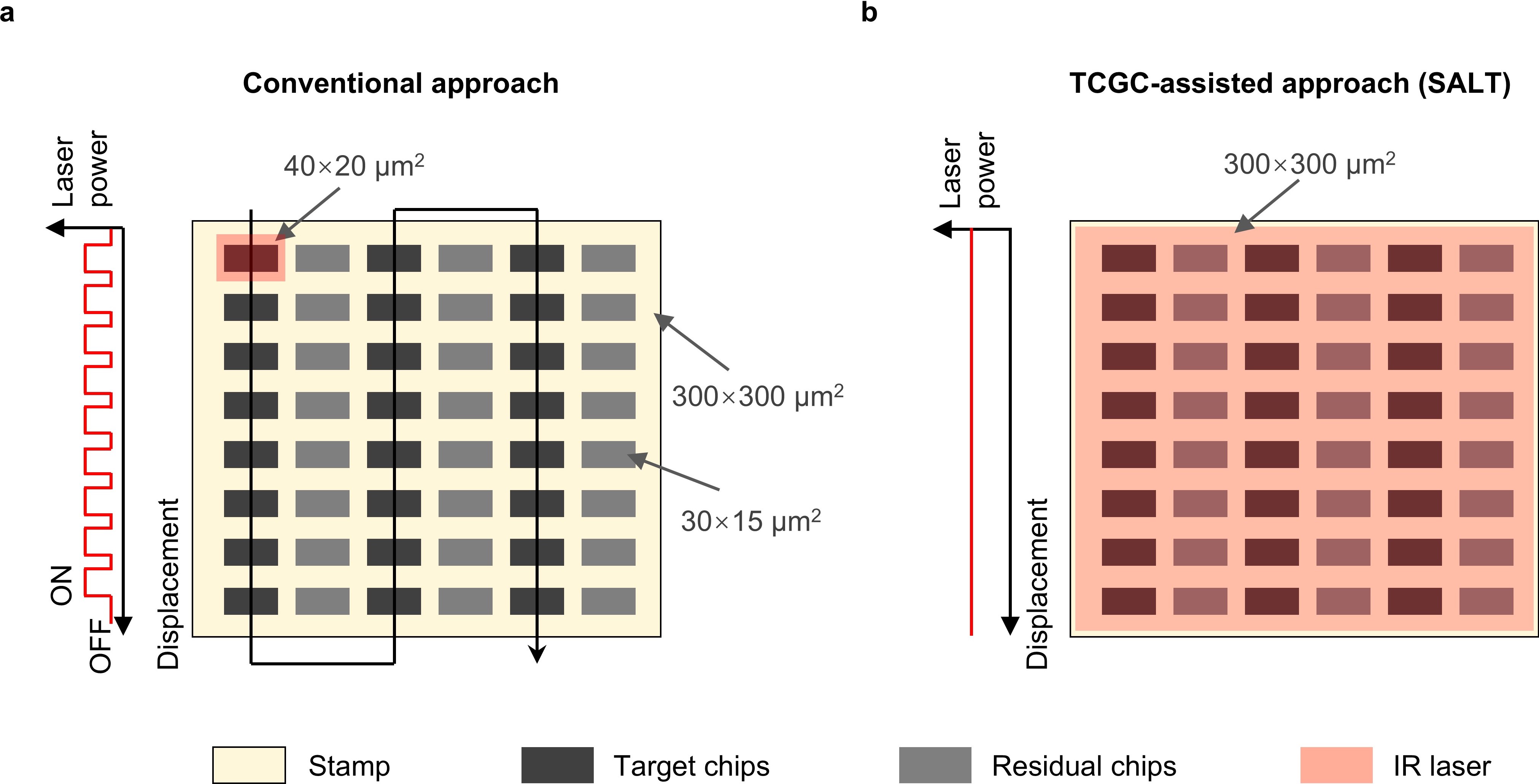


Fig. S44 Comparison of conventional approach (pre-planned scanning paths) and SALT in the batch-selective transfer of MicroLEDs (30 µm× 15 µm). a Schematic illustration of the conventional approach utilizing a small spot (40 µm× 20 µm) for the batch selective transfer of target chips based on a pre-planned scanning path. b Schematic illustration of SALT utilizing a large flat-top spot (300 µm× 300 µm) for the batch selective transfer of target chips based on grayscale-controlled TCGC.


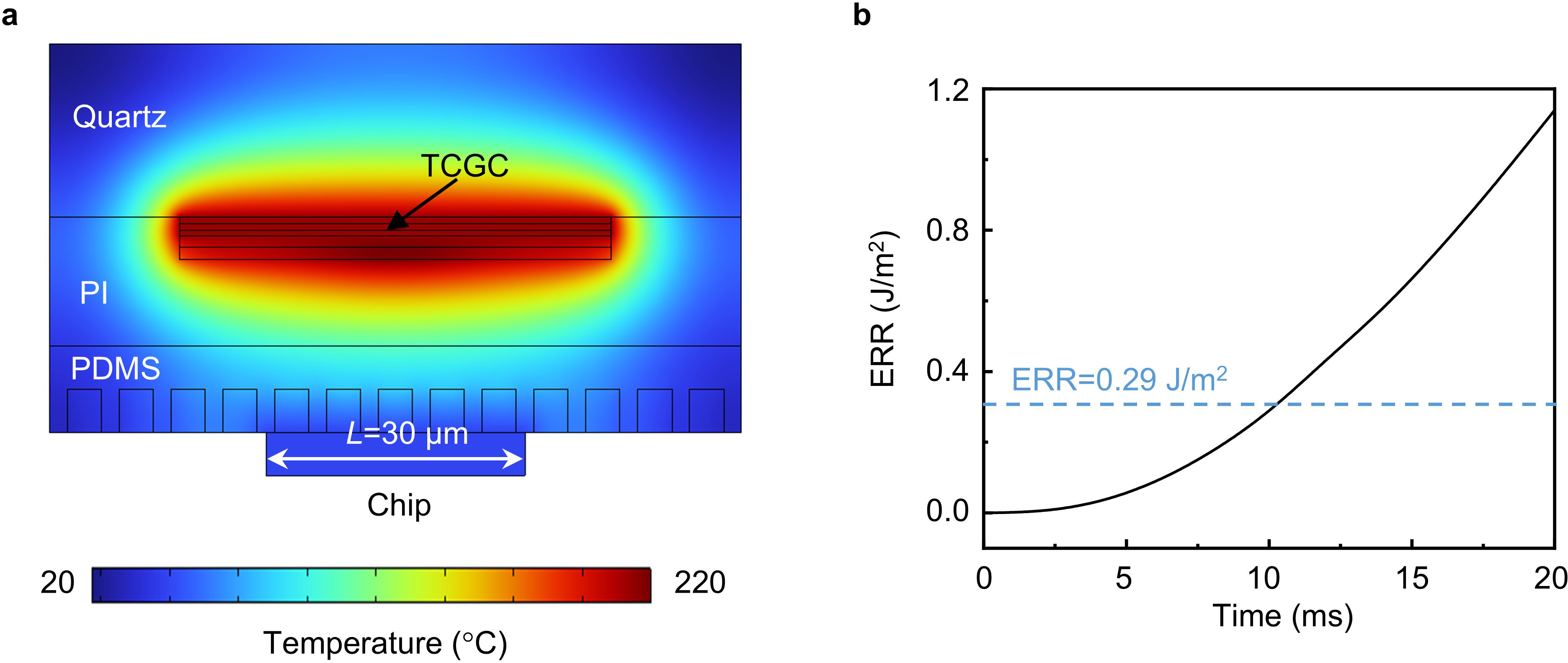


Fig. S45 Finite element analysis of the transfer time for MicroLED under a 5-mW IR laser irradiation. a Simulated temperature distribution of TCGC-embedded adhesive stamp and MicroLED. b Energy release rate of the stamp-chip interface crack tip.


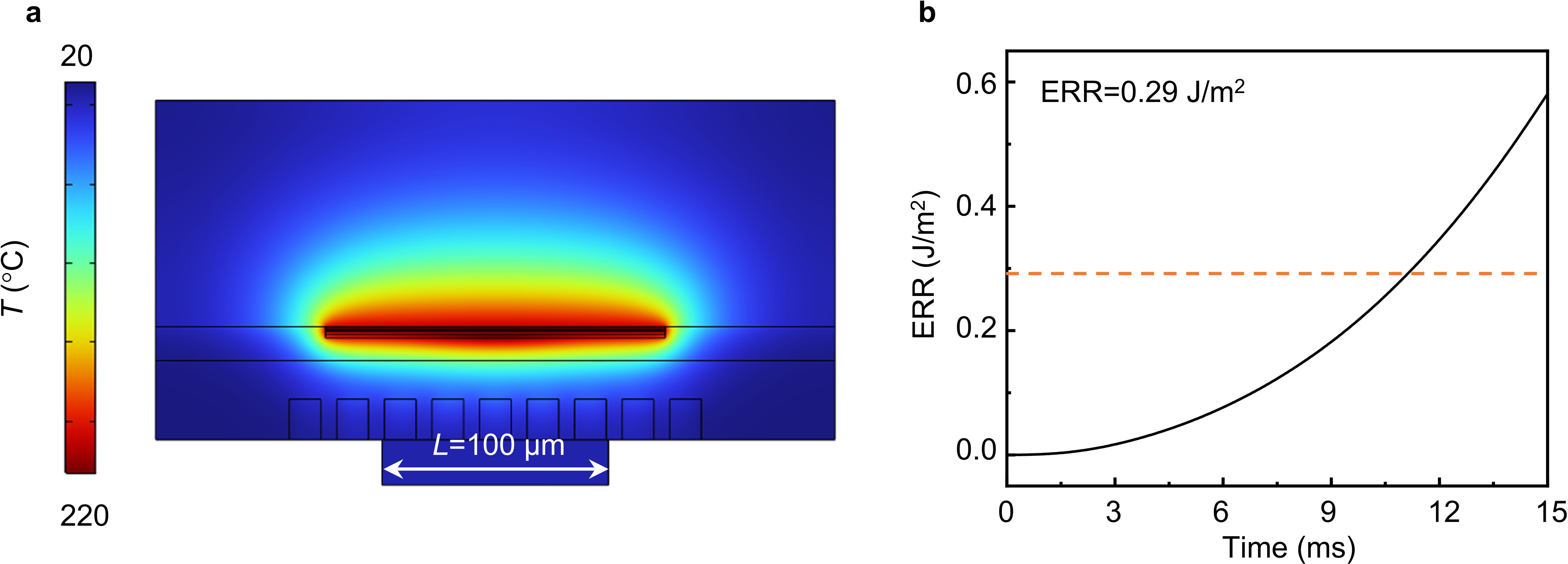


Fig. S46 Effect of IR laser irradiation for a TCGC embedded adhesive stamp/small-size chip (100 µm × 100 µm × 20 µm). a Simulated temperature field of the stamp and chip under a 0.3-W IR irradiation. b Energy release rate of the stamp-chip interface crack tip.

Table S1 Statistics of key performance metrics for different laser-driven non-contact transfer techniques.

| Mechanism | Reversibility | Transfer time (ms) | Energy density (J·cm^-2^) | Adhesive switching ratio | Transfer accuracy (µm) | Limited by alignment accuracy | Refs |
| --- | --- | --- | --- | --- | --- | --- | --- |
| Photothermal | Yes | 100 | 32.653 | 1000 | Visible error | Yes | 3 |
| Photothermal | Yes | 15 | 8.344 | >1000 | Visible error | Yes | 4 |
| Photothermal | Yes | 19 | 43.225 | 17.3 | Visible error | Yes | 5 |
| Photothermal | Yes | 5 | 23.025 | / | / | Yes | 7 |
| Photothermal | Yes | 1000 | 25 | ~1000 | / | Yes | 8 |
| Photothermal | Yes | 3.5 | 350 | / | ~50 | Yes | 9 |
| Photothermal | No | <1 | / | / | 56.75 | Yes | 10 |
| Photochemical | No | <1 | ~0.53 | / | 22.4 | Yes | 11 |
| Photochemical | No | <1 | 0.002 | / | 19.03 | Yes | 12 |
| Photochemical | No | <1 | / | / | 61.2 | Yes | 13 |
| Photothermal | Yes | 32 | 4 | 650 | 4.6 | No | This work |

Table S2 Comparison of transfer time of MicroLED array across a specific area (300 µm × 300 µm) via conventional approach and SALT.

|  | **Conventional approach** | **SALT** |
| --- | --- | --- |
| Laser Spot Size | 40 µm× 20 µm | 300 µm× 300 µm |
| Single laser  irradiation duration | 10 ms | 10 ms |
| Number of irradiations | 24 | 1 |
| Total transfer time | >240 ms | >10 ms |

Table S3 Important parameters used in this study.

| Parameter | Value | Refs. |
| --- | --- | --- |
| Density of PI *ρ* [g·cm^-3^] | 1.42 | 14 |
| Density of quartz *ρ* [g·cm^-3^] | 2.37 | 14 |
| Density of PDMS *ρ*[g·cm^-3^] | 0.97 | 7 |
| Density of graphene *ρ*[g·cm^-3^] | 2.21 | 15 |
| Density of Si chip *ρ*[g·cm^-3^] | 2.329 | 7 |
| Specific heat of PI *C*_p_ [J·g^-1^·K^-1^] | 2.55-1.59 × exp[(*T*_0_-*T*)/460] | 14 |
| Specific heat of quartz *C*_p_ [J·g^-1^·K^-1^] | 1.53-0.79 × exp[(*T*_0_-*T*)/638] | 14 |
| Specific heat of PDMS *C*_p_ [J·g^-1^·K^-1^] | 1460 | 7 |
| Specific heat of graphene *C*_p_ [J·g^-1^·K^-1^] | 0.709 | 15 |
| Specific heat of Si chip *C*_p_ [J·g^-1^·K^-1^] | 0.703 | 7 |
| Thermal conductivity of PI *κ* [W·cm^-1^·K^-1^] | 1.55 × 10^-3^(*T*/*T*_0_)^0.28^ | 14 |
| Thermal conductivity of quartz *κ* [W·cm^-1^·K^-1^] | 7.9 × 10^-4^*T*^0.43^ | 14 |
| Thermal conductivity of PDMS *κ* [W·cm^-1^·K^-1^] | 0.0015 | 7 |
| Thermal conductivity of Gr *κ* [W·cm^-1^·K^-1^] | {26.4 × (*T*/*T*_0_)^-1.38^, 0.06, 26.4 × (*T*/*T*_0_)^-1.38^} | 15 |
| Thermal conductivity of AC *κ* [W·cm^-1^·K^-1^] | 0.0005 | 16 |
| Thermal conductivity of Si chip *κ* [W·cm^-1^·K^-1^] | 1.63 | 7 |
| Young's modulus of PI *E* [GPa] | 5 | 17 |
| Young's modulus of quartz *E* [GPa] | 220 | 17 |
| Young's modulus of PDMS *E* [GPa] | 0.002 | 7 |
| Young's modulus of Si chip *E* [GPa] | 179.4 | 7 |
| Poisson's ratio of PI *μ* | 0.35 | Comsol |
| Poisson's ratio of quartz *μ* | 0.23 | Comsol |
| Poisson's ratio of Si chip *μ* | 0.28 | 7 |
| Poisson's ratio of PDMS chip *μ* | 0.48 | 7 |
| Cohesive Energy release rate [J·m^-2^] | 0.29 | Test |

SI References

1. Nakata, Y., Miyanaga, N. & Osawa, K. Numerical simulation of an adaptive beam-shaping technique using a phase grating overlapped via a spatial light modulator for precision square–flat-top beam. *Applied Physics A* **126**, 317 (2020).

2. Maugis, D. Contact, Adhesion and Rupture of Elastic Solids. (Berlin, Heidelberg: Springer, 2000).

3. Luo, H. Y. et al. Laser-driven programmable non-contact transfer printing of objects onto arbitrary receivers via an active elastomeric microstructured stamp. *National Science Review* **7**, 296-304 (2020).

4. Luo, H. Y. et al. Switchable Adhesive Based on Shape Memory Polymer with Micropillars of Different Heights for Laser-Driven Noncontact Transfer Printing. *ACS Applied Materials & Interfaces* **16**, 9443-9452 (2024).

5. Li, C. L. et al. Laser-driven noncontact bubble transfer printing via a hydrogel composite stamp. *Proceedings of the National Academy of Sciences of the United States of America* **121**, e2318739121 (2024).

6. Li, C. L. et al. Laser-induced adhesives with excellent adhesion enhancement and reduction capabilities for transfer printing of microchips. *Science Advances* **10**, eads9226 (2024).

7. Luo, H. Y. et al. Thermal controlled tunable adhesive for deterministic assembly by transfer printing. *Advanced Functional Materials* **31**, 2010297 (2021).

8. Zhang, S. et al. A thermal actuated switchable dry adhesive with high reversibility for transfer printing. *International Journal of Extreme Manufacturing* **3**, 035103 (2021).

9. Saeidpourazar, R. et al. Laser-driven micro transfer placement of prefabricated microstructures. *Journal of Microelectromechanical Systems* **21**, 1049-1058 (2012).

10. Cao, Y. X. & Zhang, Z. Noncontact selective laser transfer printing and assembly of micro-sized semiconductor devices. Proceedings of 2022 18th IEEE/ASME International Conference on Mechatronic and Embedded Systems and Applications (MESA). Taipei, China: IEEE, 2022, 1-6.

11. Marinov, V. R. et al. Laser-assisted ultrathin die packaging: Insights from a process study. *Microelectronic Engineering* **101**, 23-30 (2013).

12. Overmeyer, L. et al. On-the-fly bare die bonding based on laser induced forward transfer (LIFT). *CIRP Annals* **71**, 41-44 (2022).

13. Miller, R. et al. Noncontact selective laser-assisted placement of thinned semiconductor dice. *IEEE Transactions on Components, Packaging and Manufacturing Technology* **2**, 971-978 (2012).

14. Bian, J. et al. Experimental and modeling study of controllable laser lift-off via low-fluence multiscanning of polyimide-substrate interface. *International Journal of Heat and Mass Transfer* **188**, 122609 (2022).

15. Kang, S. M. et al. Graphene-enabled laser lift-off for ultrathin displays. *Nature Communications* **15**, 8288 (2024).

16. Balandin, A. A. Thermal properties of graphene and nanostructured carbon materials. *Nature Materials* **10**, 569-581 (2011).

17. Chen, F. R. et al. Laser-driven hierarchical “gas-needles” for programmable and high-precision proximity transfer printing of microchips. *Science Advances* **9**, eadk0244 (2023).

Legends for Movies S1 to S2

**Movie S1** Thermal imaging video of the quartz-TCGC-PI sample irradiated by an infrared laser from the front/back (through the quartz/PI substrate).

**Movie S2** High-speed video of the chip transfer process by the stamp with/without TCGC under misaligned infrared laser irradiation.
